# Stem cell–derived CAR T cells traffic to HIV reservoirs in macaques

**DOI:** 10.1172/jci.insight.141502

**Published:** 2021-01-11

**Authors:** Isaac M. Barber-Axthelm, Valerie Barber-Axthelm, Kai Yin Sze, Anjie Zhen, Gajendra W. Suryawanshi, Irvin S.Y. Chen, Jerome A. Zack, Scott G. Kitchen, Hans-Peter Kiem, Christopher W. Peterson

**Affiliations:** 1 Stem Cell and Gene Therapy Program, Fred Hutchinson Cancer Research Center, Seattle, Washington, USA.; 2 Department of Comparative Medicine, University of Washington, Seattle, Washington, USA.; 3 Department of Medicine, Division of Hematology and Oncology, David Geffen School of Medicine at University of California, Los Angeles, California, USA.; 4 UCLA AIDS Institute, Los Angeles, California, USA.; 5 Department of Microbiology, Immunology and Molecular Genetics, David Geffen School of Medicine at University of California, Los Angeles, California, USA.; 6 Department of Medicine and; 7 Department of Pathology, University of Washington, Seattle, Washington, USA.

**Keywords:** AIDS/HIV, Stem cells, Cell migration/adhesion, Gene therapy, Hematopoietic stem cells

## Abstract

Allogeneic hematopoietic stem cell transplantation (allo-HSCT) with CCR5^–^ donor cells is the only treatment known to cure HIV-1 in patients with underlying malignancy. This is likely due to a donor cell–mediated graft-versus-host effect targeting HIV reservoirs. Allo-HSCT would not be an acceptable therapy for most people living with HIV due to the transplant-related side effects. Chimeric antigen receptor (CAR) immunotherapies specifically traffic to malignant lymphoid tissues (lymphomas) and, in some settings, are able to replace allo-HSCT. Here, we quantified the engraftment of HSC-derived, virus-directed CAR T cells within HIV reservoirs in a macaque model of HIV infection, using potentially novel IHC assays. HSC-derived CAR cells trafficked to and displayed multilineage engraftment within tissue-associated viral reservoirs, persisting for nearly 2 years in lymphoid germinal centers, the brain, and the gastrointestinal tract. Our findings demonstrate that HSC-derived CAR^+^ cells reside long-term and proliferate in numerous tissues relevant for HIV infection and cancer.

## Graphical abstract

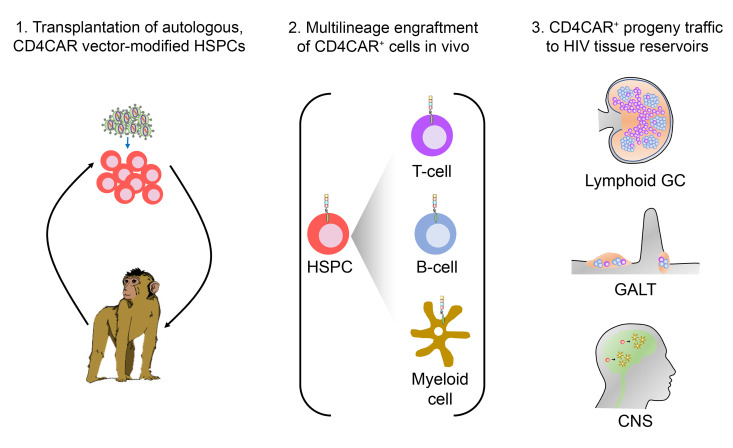

## Introduction

Hematopoietic stem and progenitor cell (HSPC) transplantation has emerged as a promising approach to achieve a functional HIV cure, largely due to the clinical cases of the Berlin Patient and the London Patient (1, 2). Each patient received HSPCs from donors who were homozygous for a naturally occurring 32-base pair deletion in the CCR5 gene (CCR5Δ32), resulting in a truncated protein that is not expressed on the cell surface. The sustained viral remission in these patients following withdrawal of suppressive antiretroviral therapy (ART) is likely due to a combination of: (a) the conditioning regimens that enabled rapid, complete donor chimerism and that may also have cleared a portion of latently infected host cells; (b) graft-versus-host–mediated elimination of latently infected host cells during donor cell engraftment (frequently referred to as graft-versus-virus or graft-versus-reservoir); and (c) near-complete replacement of the host immune system with homozygous CCR5Δ32 donor cells, conferring resistance to CCR5-tropic HIV-1 (3). While the successful treatment of these 2 patients marks a significant milestone in efforts aimed at an HIV cure, there are several limitations that render this approach infeasible for the vast majority of people living with HIV. These include significant risk of morbidity and mortality associated with allo-HSCT, the limited prevalence of CCR5Δ32 donors, and the potential for the virus to circumvent the CCR5Δ32 mutation via a CCR5- to CXCR4-tropism shift (4). We are interested in applying principles from the successful treatment of the Berlin and London patients, using alternative approaches that are comparably potent, less toxic, and applicable to a larger patient population.

Expression of HIV-specific chimeric antigen receptors (CARs) from gene-modified, autologous HSPCs has the potential to overcome each of the limitations associated with allo-HSCT. CARs containing a CD4 extracellular and transmembrane domain fused to the CD3ζ signal transduction domain (CD4CAR) effectively redirect primary T lymphocytes to lyse HIV-infected cells in vitro (5, 6). These CAR molecules, as well as CARs based on anti-HIV broadly neutralizing antibodies (7–9), bind to the HIV Env protein expressed on the surface of infected cells, which permits CAR-mediated target cell lysis independently of MHC presentation and, thus, circumventing HIV-mediated MHC I downregulation. Previously, we reported similar impacts in nonhuman primates (NHPs) infected with simian/human immunodeficiency virus (SHIV) and transplanted with CD4CAR HSPCs, including expansion of CAR^+^ effector cells, reduced tissue viral loads, and reduced rebound plasma viremia following discontinuation of ART (10). These findings demonstrate that HSC-derived CD4CAR cells can exert an antiviral effect in vivo.

We reasoned that genetically modifying HSPCs to express CD4CAR permits continuous production of CAR T cells that undergo normal physiologic development, circumventing the need for significant ex vivo expansion that is currently required for adoptively transferred CAR T cell therapies. However, the ability of these cells to traffic to and persist in HIV reservoir tissues remains unclear. Multiple tissue sites have been identified as potential HIV reservoirs, including lymphoid germinal centers (GCs) (11–21), the CNS (16, 22–27), and the gastrointestinal tract (GIT) (16, 17, 28–33). The physiologic properties that allow these sites to function as HIV reservoirs may include one or more of the following: (a) resident cell populations that support latent viral infection and/or active viral replication after prolonged combination ART; (b) resident cells that retain infectious virions for prolonged periods of time; and (c) the immune privileged nature of certain sites that exclude effector cells necessary for viral clearance. As with any approach designed to control viral replication in the absence of ART (termed functional cure or functional remission), CAR^+^ cells must traffic to HIV tissue reservoir sites, engraft and persist, and recognize and kill infected cells. Here, we extended our previous evaluation of the safety and efficacy of HSC-derived, CD4CAR-expressing T lymphocytes in SHIV-infected NHP (10), focusing on tissues that were collected at study endpoint. Our objective was to quantify the trafficking and persistence of HSC-derived, CAR-expressing cells in HIV tissue reservoir sites, utilizing CD4CAR-specific IHC-based assays. We show that HSC-derived, multilineage CD4CAR^+^ cells traffic to and persist long-term in lymphoid GCs, CNS tissue, and GIT tissue and actively proliferate within these HIV reservoir sites.

## Results

### Overview of study animals and necropsy data.

**Figure 1:**
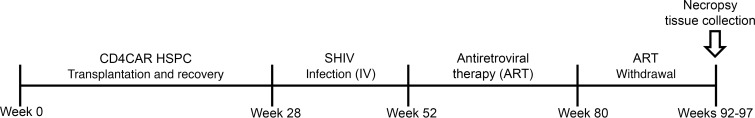
Study schematic for CAR 1, CAR 2, Control 1, and Control 2 animals. A total of 4 pigtail macaques were transplanted with autologous HSPCs modified to express CD4CAR (*n =* 2) or a control CD4CARΔζ (*n =* 2), which lacks intracellular signaling function but retains the extracellular domain for immunolabeling. Following 28 weeks of posttransplant recovery, animals were infected with SHIV-1157ipd3N4 via the i.v. route. Approximately 6 months later, antiretroviral therapy (ART) was initiated, then withdrawn 28 weeks later, in order to compare the persistence of CD4CAR and control-modified cells in low- and high-antigen conditions, respectively. Following ART withdrawal, animals were monitored for approximately 15 weeks prior to necropsy.

The data presented here build upon a previously published experiment focused on 4 pigtail macaques that were transplanted with autologous, lentiviral vector–modified HSPCs. Two animals, referred to as CAR 1 and CAR 2, received CD4CAR-transduced HSPCs, while 2 controls (Control 1 and Control 2) received a signaling defective CAR known as CD4CARΔζ. A study schematic for this cohort is shown in Figure 1. Infused HSPC products contained a range of 4.7%–40.0% gene-marked cells; following infusion into the total body irradiation–conditioned (TBI-conditioned) autologous host, each animal recovered with typical kinetics (Supplemental Table 1; supplemental material available online with this article; https://doi.org/10.1172/jci.insight.141502DS1). Approximately 28 weeks after transplant, each animal was infected with SHIV, at which point gene marking levels were low (0.1%–0.8% by flow cytometry and 1.55%–8.79% by PCR). Integration site analyses demonstrated that gene-marked peripheral blood cells from both CAR 1 and CAR 2 displayed a polyclonal integration pattern (Supplemental Figure 1). All of the samples in the present study were collected at necropsy, following primary SHIV infection, ART suppression, ART release, and viral rebound (Figure 1). Necropsy data from our previously published work are summarized in Supplemental Table 2 and Supplemental Figures 2 and 3 (10). SHIV plasma viral loads at necropsy ranged from 999 to 884,846 copies/mL plasma (Supplemental Table 2). SHIV DNA (Supplemental Figure 2A) and SHIV RNA (Supplemental Figure 2B) were detectable, often at high levels, in a panel of 25 tissues, as well as 4-limb BM and PBMC. CD4CAR gene marking levels in total PBMC ranged from 0.64% to 2.71% by flow cytometry and from 2.72% to 15.63% by PCR (Supplemental Table 2). Higher gene marking levels by PCR are expected, due to (a) the subset of integrated vector proviruses that have been silenced or accrued mutations that prevent transgene expression and (b) the fact that > 1 integration event per cell is detectable by PCR but may not be detected by flow cytometry (34). Among CD4CAR^+^ PBMC, the majority of CAR^+^ cells were CD20^+^ B cells, followed by CD4^+^ and CD8^+^ T cells, CD2^+^NKG2A^+^ NK cells, and CD14^+^ monocytes (Supplemental Figure 3). Our prior studies demonstrated that each of the 4 animals recovered comparably following autologous HSPC transplantation and confirmed that both SHIV-infected target cells and CD4CAR^+^ effector cells were present at end-of-study necropsy. Necropsy samples from this cohort were, hence, well suited to build and validate an assay to quantify the trafficking of CD4CAR^+^ cells, which was the main objective of this work.

### Validation of CD4CAR IHC assay.

**Figure 2:**
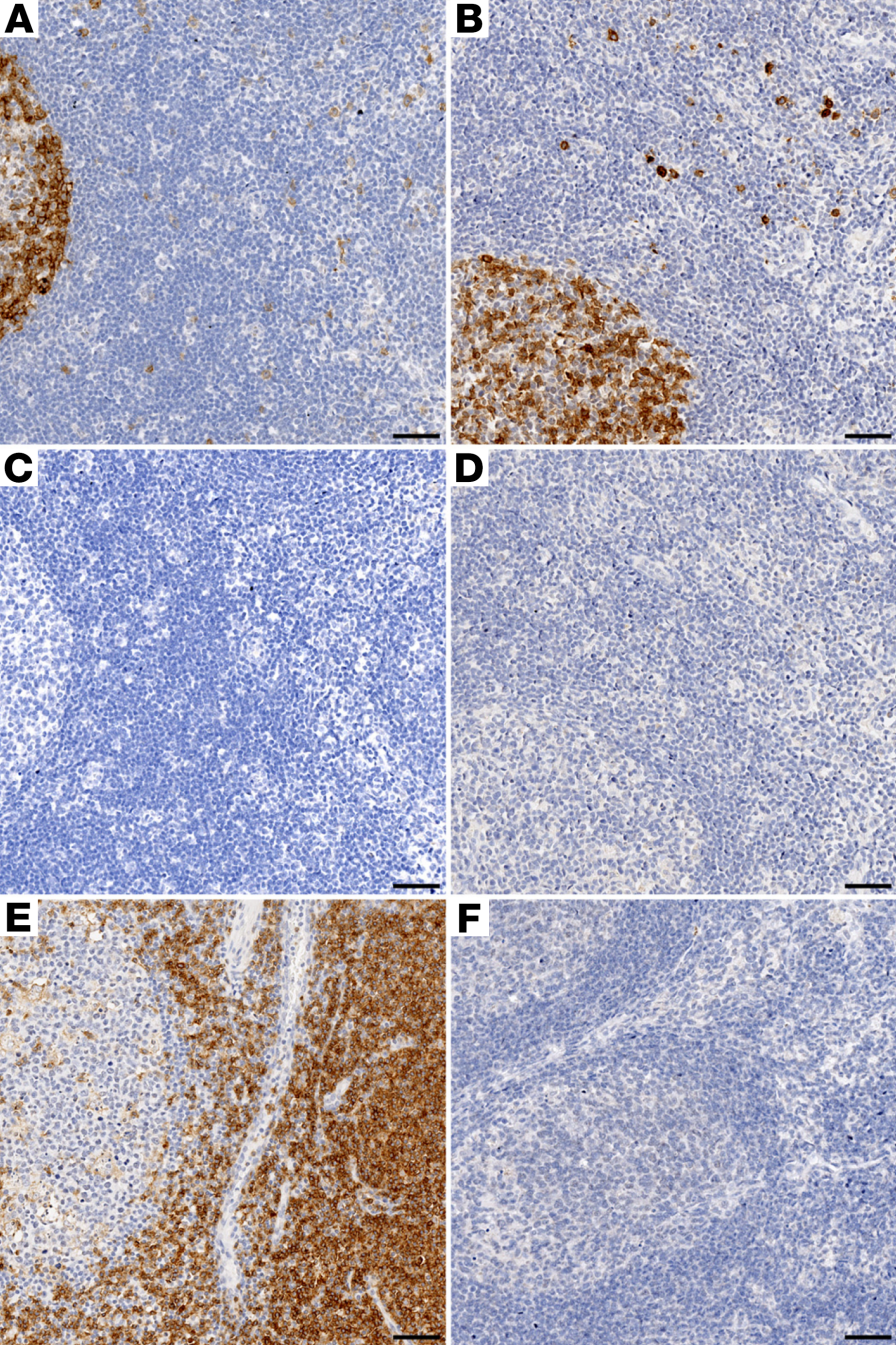
Anti-CD4 antibody clone SP35 specifically marks CAR^+^ cells. (**A** and **B**) Specific CD4 (SP35) immunoreactivity in germinal centers from mesenteric lymph node sections from macaques that received either CD4CAR (**A**) or CD4CARΔζ (**B**); sparse marking in the parafollicular zone was also observed. (**C** and **D**) No immunoreactivity was seen in paired adjacent CD4CAR (**C**) or CD4CARΔζ (**D**) tissue sections labeled with an isotype control. (**E**) Positive control: Labeling of human tonsil shows specific immunoreactivity, which is predominately in the parafollicular zone and consistent with CD4^+^ T cell marking. (**F**) Negative control: no immunoreactivity is seen in a control mesenteric lymph node section from a macaque that did not receive either CD4CAR or CD4CARΔζ, indicating that the CD4 (SP35) antibody clone does not cross-react with the endogenous pigtail macaque CD4 antigen. Brown, immunoreactivity for human CD4CAR; blue, hematoxylin counterstain. The experiment was repeated twice to confirm the specificity of the CD4 (SP35) antibody for the human-derived CDCAR or CD4CARΔζ. Scale bars: 50 μm.

The CD4CAR construct utilized for these studies expresses a cell surface protein consisting of a human CD4 extracellular and transmembrane domain, fused to a human CD3ζ signal transduction domain, which has previously been tested in clinical trials and in NHPs (7, 10). We designed our IHC assay to specifically detect the human CD4 extracellular domain of CD4CAR, while minimizing background from endogenous NHP CD4 molecules. To confirm specific labeling of the human CD4 extracellular domain, we stained lymphoid tissue sections collected at necropsy from CAR 1, CAR 2, Control 1, and Control 2. We applied a monoclonal anti-CD4 antibody (clone SP35) and evaluated for CD4CAR-specific immunoreactivity (10), which was observed in tissues from macaques that received signaling-proficient CD4CAR (CAR 1 and CAR 2) and animals that received the signaling-defective CD4CARΔζ, which retains the extracellular CD4 domain but lacks the CD3ζ signal transduction domain (Control 1 and Control 2) (Figure 2, A and B). Importantly, CD4CARΔζ should still be labeled with our SP35 antibody clone but should not facilitate intracellular signaling in response to antigen binding. No signal was observed in control samples stained with a nonspecific, isotype-matched rabbit antibody (Figure 2, C and D). Positive control sections of human tonsil showed CD4-specific immunoreactivity predominately in the paracortex, consistent with specific antibody binding to the human CD4 antigen in the CAR-modified animals (Figure 2E). Importantly, lymphoid tissues from HSPC-transplanted pigtail macaques that did not receive CD4CAR-transduced cells did not display any antigen-specific immunoreactivity (Figure 2F). These data show that anti-CD4 SP35 immunoreactivity observed in tissues from CD4CAR-transduced animals is due to specific CAR labeling and not to cross-reactivity with the endogenous macaque CD4 antigen or nonspecific binding from the secondary antibody.

### Multilineage engraftment of HSC-derived CAR^+^ cells in lymphoid GCs.

**Figure 3:**
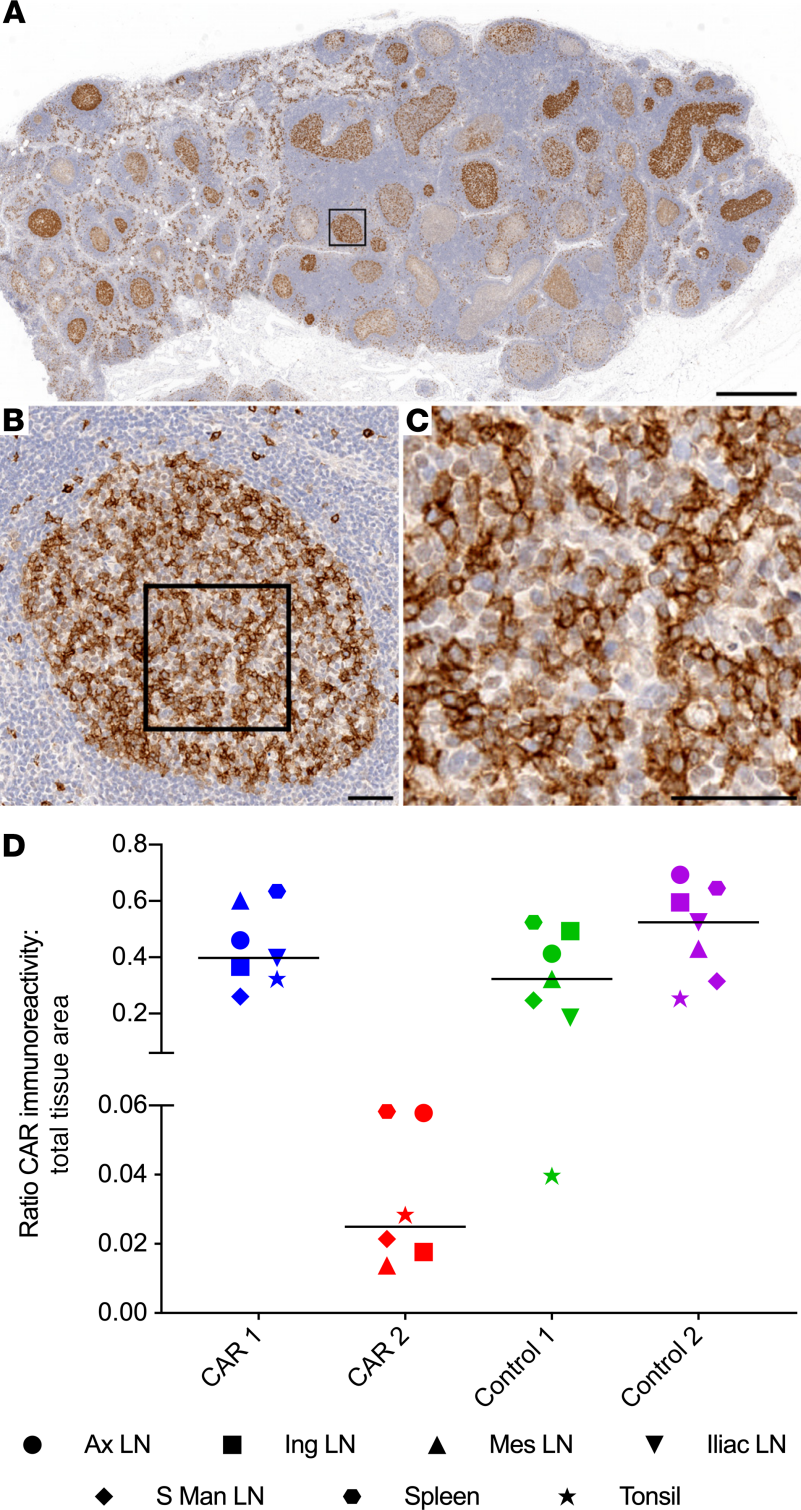
HSPC-derived CD4CAR expressing cells localize to central and peripheral lymphoid tissue germinal centers. (**A**–**C**) Low- (**A**), medium- (**B**), and high-magnification (**C**) photomicrographs of an iliac LN from CAR 1, illustrating CD4CAR^+^ cells localizing in the germinal centers. Brown, immunoreactivity for human CD4CAR; blue, hematoxylin counterstain. (**D**) Quantification of CD4CAR-labeled lymphoid GCs as a percentage of total lymphoid GC tissue area in both CD4CAR and CD4CARΔζ macaques (*n =* 4 macaques; 6–7 lymphoid tissues per macaque). Thresholds for average percentage CD4CAR GC marking were set using representative lymphoid tissues from macaques that did not receive a CAR (0.0031%). The chart shows individual data points with the median. Scale bars: 1 mm (**A**); 50 μm (**B** and **C**).

Next, we applied our CD4CAR-specific IHC assay to quantify trafficking of HSC-derived, CAR^+^ cells to lymphoid GCs. We stained paraffin-embedded sections from secondary lymphoid organs from 2 CD4CAR macaques (CAR 1 and CAR 2), and 2 control CD4CARΔζ macaques (Control 1 and Control 2), using the anti-CD4 SP35 monoclonal antibody to detect CD4CAR^+^ cells. Tissues from macaques that did not receive CD4CAR or CD4CARΔζ were used as a threshold for CAR-specific marking. We observed CD4CAR immunoreactivity in the GCs of all tissues in all 4 macaques (CD4CAR and CD4CARΔζ) (Figure 3, A–C). Within individual tissue sections, the amount of CD4CAR immunoreactivity ranged from absent to occupying almost the entire area of a given GC. We used a previously described brightfield IHC quantification approach (35, 36) to estimate the amount of CAR marking in GCs. This pixel-based area analysis demonstrated that 1.4%–62.6% of the total GC tissue area was positive for CD4CAR protein expression. The most robust CD4CAR marking was observed in tissues from CAR 1 and both control animals (Figure 3D and Supplemental Figure 4A). No differences were observed in the frequency of GC CAR staining between CD4CAR and CD4CARΔζ animals, aside from the overall reduced CAR marking in tissues from CAR 2. However, all 4 animals had CAR marking frequencies above the threshold set by the unmodified control tissues, suggesting that HSC-derived CAR^+^ cells traffic to lymphoid GCs in a manner independent of CD4CAR signaling.

**Figure 4:**
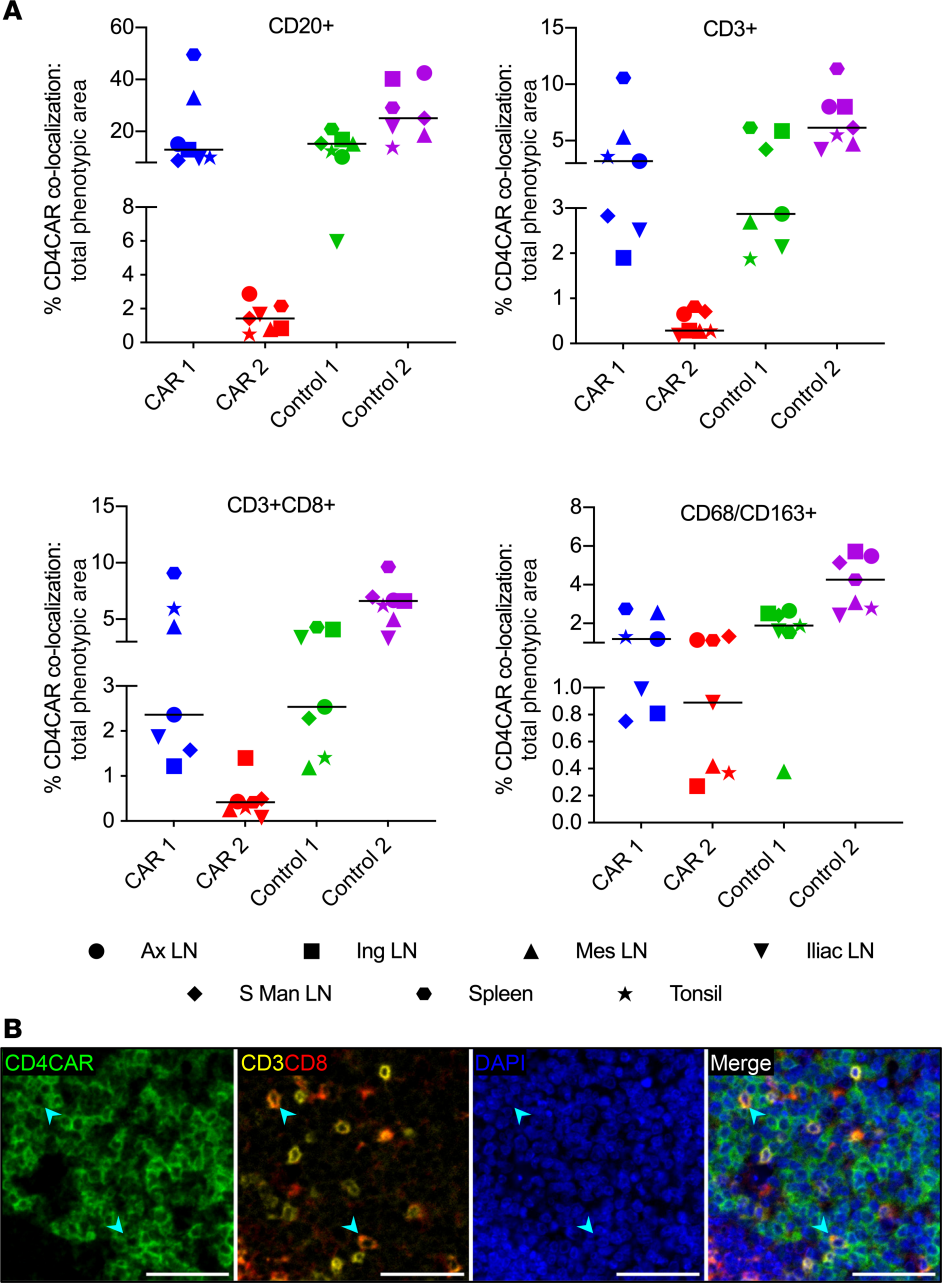
Multilineage engraftment of HSPC-derived CAR^+^ cells in lymphoid germinal centers. (**A**) Percentages of total B cell (CD20^+^), T cell (CD3^+^), CTL (CD3^+^CD8^+^), and monocyte/macrophage (CD68/CD163^+^) immunophenotypic area that colocalized with CD4CAR immunoreactivity in GCs (*n =* 4 macaques; 6–7 lymphoid tissues per macaque). The charts show individual data points with medians. (**B**) Representative fluorescent mIHC photomicrographs of CD4CAR (green), CD3 (yellow), CD8 (red), and DAPI nuclear counterstain (blue), from CAR 1 mesenteric LN. Arrowheads indicate colocalization between CD4CAR and CD3/CD8 markers, showing the presence of CD4CAR^+^ CTLs within the germinal center. Scale bars: 50 μm.

Following quantification of CD4CAR localization in various secondary lymphoid tissues (Figure 3 and Supplemental Figure 4, A and D), we next set out to identify the lymphoid and myeloid subsets that expressed the CAR transgene within each site (Figure 4 and Supplemental Figure 5). We applied a fluorescent multiplex IHC (mIHC) approach with pixel-based quantitation to enumerate CD4CAR^+^ B cells (CD20^+^), total T cells (CD3^+^), cytotoxic T lymphocytes (CTL, CD3^+^CD8^+^), CD4 and double-negative T cells (CD3^+^CD8^–^), and monocytes/macrophages (CD68^+^CD163^+^) (Supplemental Tables 3 and 4). We could not directly stain for NHP CD4, due to cross-reactivity with CD4CAR. Between 26.2% and 66.6% of the CD4CAR^+^ area within the lymphoid GCs did not colocalize with any of the phenotypic markers. Of the remaining CD4CAR^+^ immunoreactivity, the majority colocalized with CD20, consistent with a predominance of B cells in lymphoid GCs (Figure 4A and Supplemental Figures 3, 5, and 6). The CD4CAR marker also colocalized with CD68^+^CD163^+^ monocyte/macrophage subsets and CD3^+^ T cells, which were further delineated into CTL and CD4^+^ and double-negative T cells (Figure 4B). These data demonstrate multilineage engraftment of HSC-derived CAR^+^ cells within lymphoid GCs, most notably CD4CAR^+^ CD3^+^ T cells and CAR^+^CD3^+^CD8^+^ CTLs — the functional HSC-derived subset of greatest therapeutic interest (10).

### HSC-derived CAR^+^ cells actively proliferate within the lymphoid GCs.

The GC reaction is characterized by early antigen-activated B cell proliferation, which displaces naive B cells to the periphery, forming the mantel zone (37). Additionally, chronic HIV and SIV infection is associated with a period of follicular hyperplasia with marked B cell expansion, which is hypothesized to be due to persistent viral antigen stimulation and CD4^+^ T cell priming (reviewed in ref. 38). To assess whether HSC-derived CAR^+^ cells in GCs actively proliferated under conditions of chronic SHIV infection, we employed a fluorescent mIHC assay using Ki-67 as a marker of cellular proliferation (Supplemental Table 3). Ki-67^+^CAR^+^ cells were readily identified in GCs from both CD4CAR and CD4CARΔζ macaques. There was no qualitative difference in the level of Ki-67^+^CAR^+^ cells between CD4CAR and CD4CARΔζ animals, aside from the reduced frequency of CAR marking observed in tissues from CAR 2 (Supplemental Figure 7A and Figure 3D). Consistent with our phenotypic analyses, Ki-67^+^CAR^+^ cells were predominately CD20^+^ B cells (Supplemental Figure 7B). Our results show that CD4CAR modification of GC B cells does not hinder proliferative responses, with the relative abundance of Ki-67^+^ GC B cells likely reflecting lymphoid hyperplasia associated with chronic SHIV infection.

### Multilineage engraftment of HSC-derived CAR^+^ cells in GIT.

**Figure 5:**
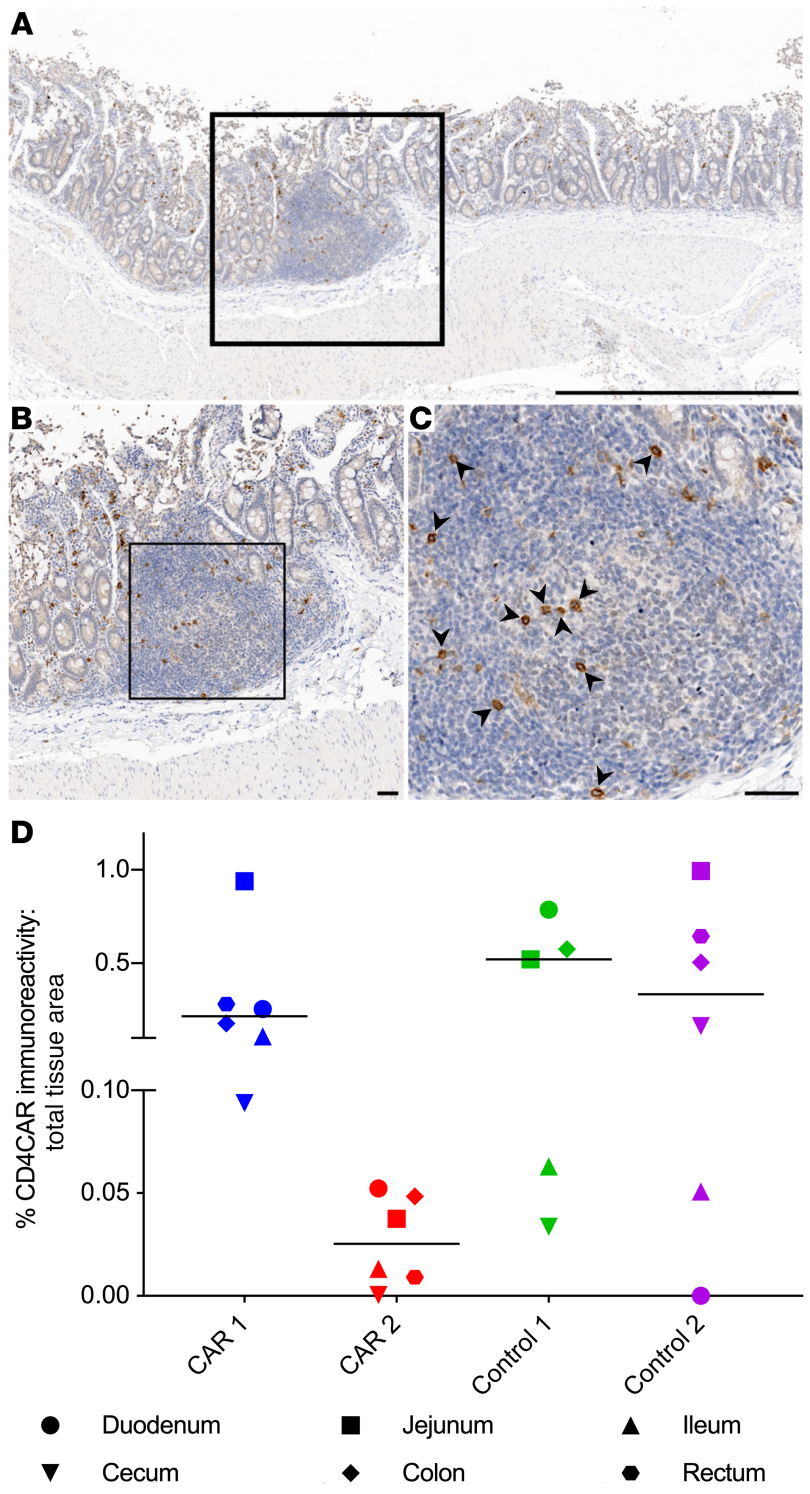
HSPC-derived CD4CAR^+^ cells localize to the GIT. (**A**–**C**) Low- (**A**), medium- (**B**), and high-magnification (**C**) photomicrographs of a jejunum section from CAR 2, illustrating localization of CD4CAR^+^ cells (arrowheads) within gastrointestinal lymphoid tissue. Brown, immunoreactivity for CD4CAR; blue, hematoxylin counterstain. (**D**) The amount of CD4CAR-labeled gastrointestinal lymphoid tissue and adjacent submucosal tissue, as a percentage of the total tissue area in both CAR and control macaques (*n =* 4 macaques; 5–6 GIT per macaque). Threshold levels were set to the average percentage of CD4CAR GIT marking in representative tissues from macaques that did not receive a CAR (0.007%). Chart shows individual data points with the median. Scale bar: 1 mm (**A**); 50 μm (**B** and **C**).

**Figure 6:**
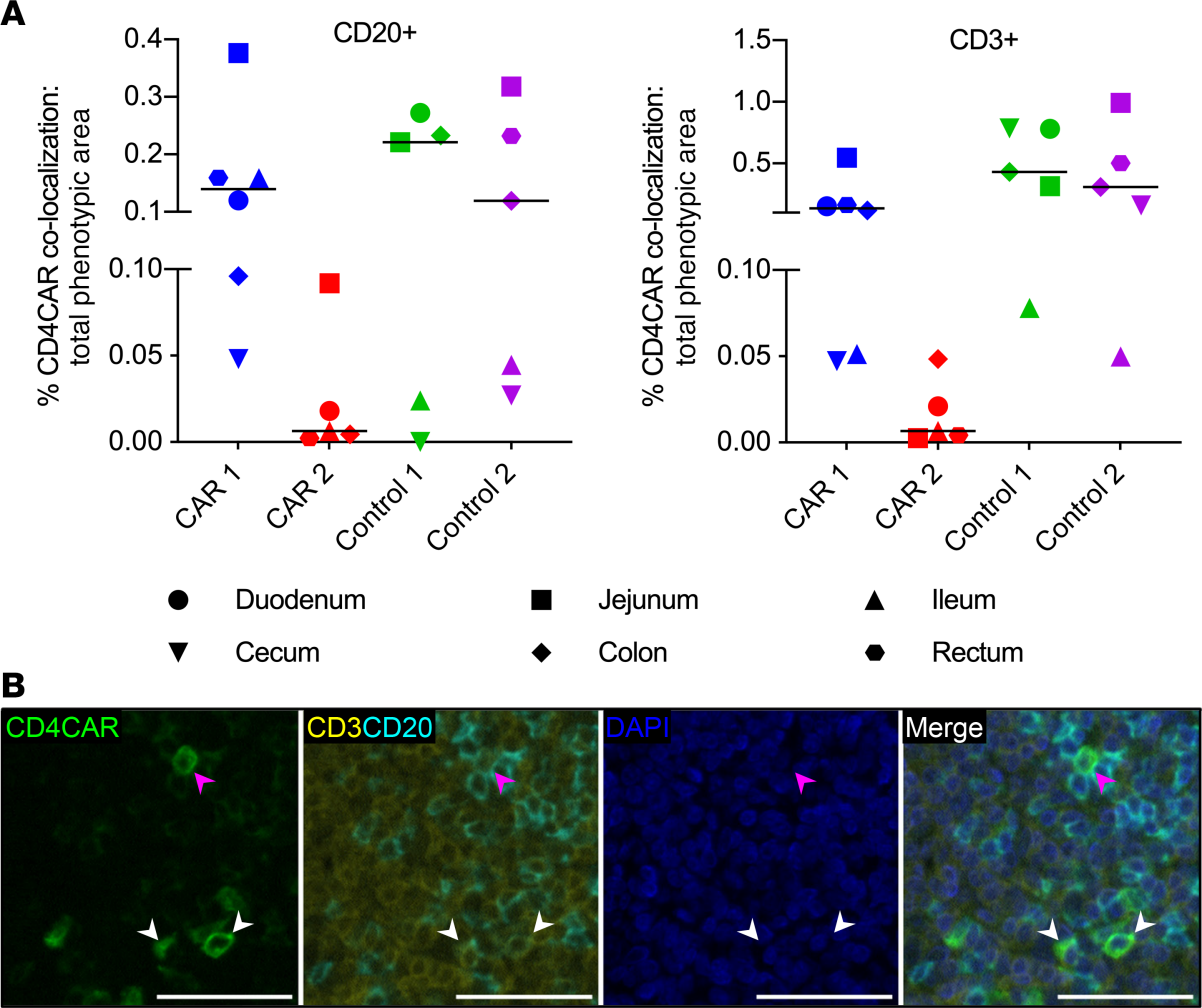
Enriched engraftment of HSPC-derived CAR^+^ T cells in GIT. (**A**) Percentages of total B cell (CD20^+^) and T cell (CD3^+^) immunoreactivity that colocalized with CD4CAR (*n =* 4 macaques; 5–6 GIT tissues per macaque). Charts show individual data points with medians. (**B**) Representative fluorescent mIHC photomicrographs of CD4CAR (green), CD3 (yellow), CD20 (cyan), and DAPI nuclear counterstain (blue), from Control 1 ileum. White arrowheads, CD4CAR^+^ T cells; magenta arrowhead, CD4CAR^+^ B cell. Scale bars: 50 μm.

Next, we evaluated HSC-derived CD4CAR^+^ cell trafficking and differentiation in 6 representative gastrointestinal lymphoid tissues (duodenum, jejunum, ileum, cecum, colon, and rectum)(Supplemental Tables 3 and 4). The ratio of CAR marking to total tissue area was determined by quantifying the amount of CAR marking using threshold fluorescence images and then dividing by the amount of threshold CAR and DAPI signal in the identical images. Tissues from macaques that did not receive a CAR had 0.007% average CAR marking, which we used as the threshold for CAR-specific marking as described above. CAR-specific immunoreactivity above threshold levels was observed in all tissue sites from CAR 1 and Control 1 (Figure 5, A–C) and in all Control 2 tissues except the duodenum. In CAR 2, marking was observed in all tissue sites except the cecum. CAR marking could not be quantified in rectal tissue from Control 1 because of a lack of gut-associated lymphoid tissue (GALT) in the sections that we evaluated. Consistent with our observations in lymphoid GCs (Figure 3D and Figure 4A), CAR 2 consistently had lower frequencies of CAR immunoreactivity compared with the other 3 animals in all GIT tissue sites (Figure 5D and Supplemental Figure 4, B and D). We evaluated the phenotypic distribution of CD4CAR^+^ cells in GIT via fluorescent mIHC, focusing on CD20 and CD3 markers to identify B cells and T cells, respectively. Between 5.4% and 58.4% of the CD4CAR^+^ area did not colocalize with CD3 or CD20. The majority of the remaining CD4CAR immunoreactive area colocalized with CD3, indicating a predominance of CAR^+^ T cells in the GALT and lamina propria, whereas a smaller proportion of CD4CAR signal colocalized with CD20 (Figure 6, A and B, and Supplemental Figures 8 and 9). These data demonstrate multilineage engraftment of HSC-derived CD4CAR^+^ cells within the small and large intestine. Importantly, the majority of phenotypically quantifiable CD4CAR^+^ cells were CD3^+^ T cells, the functional subset of greatest therapeutic interest for CD4CAR-based clearance of persistently HIV/SHIV-infected targets.

### Trafficking of HSC-derived CAR^+^ cells to CNS tissues.

**Figure 7:**
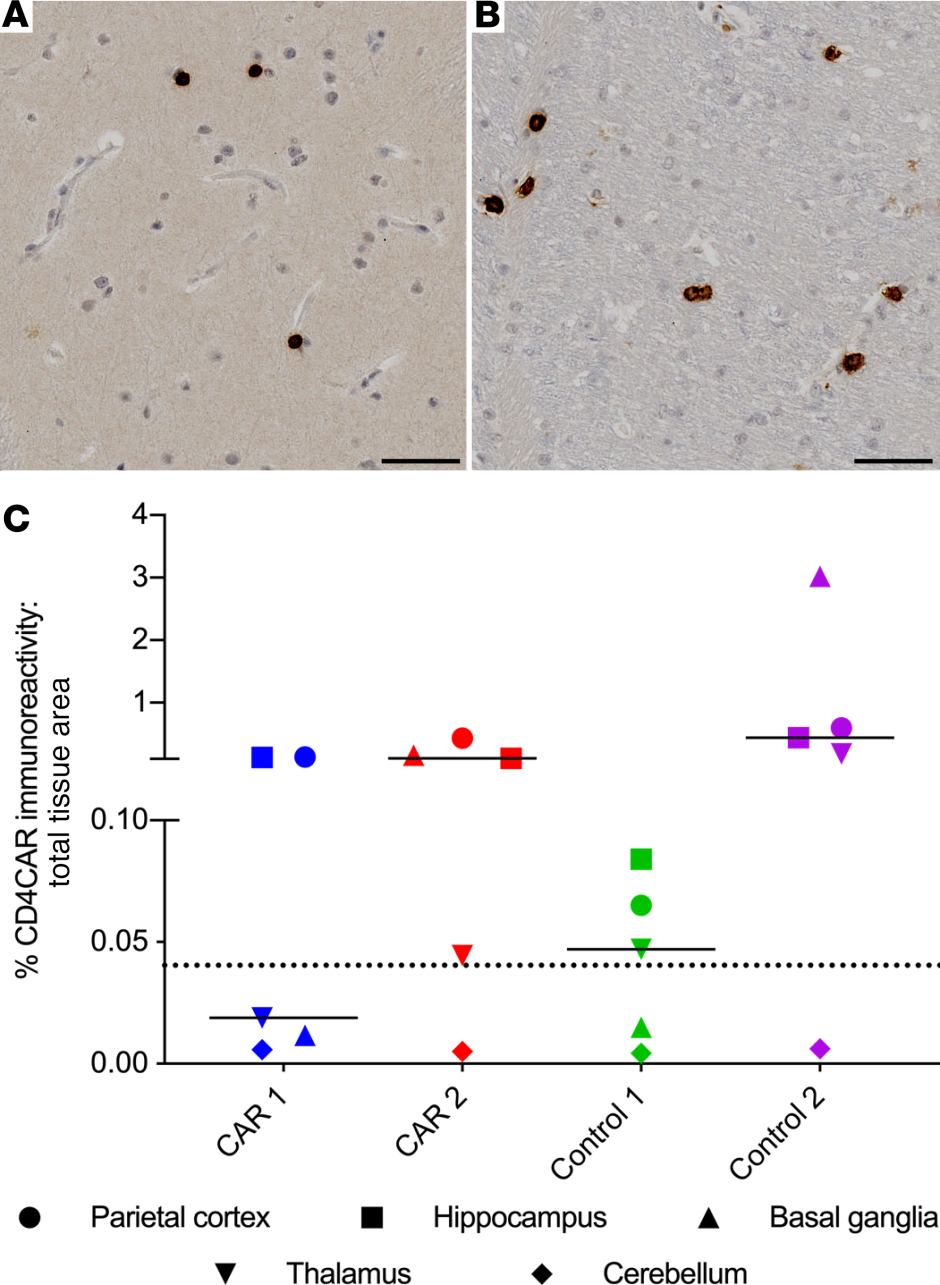
HSPC-derived CD4CAR^+^ cells engraft and persist in the CNS. (**A** and **B**) Representative photomicrographs of a basal ganglia section from Control 2, illustrating CD4CAR^+^ cells localizing to gray (**A**) and white (**B**) matter in the CNS. Brown, immunoreactivity for human CD4CAR; blue, hematoxylin counterstain. (**C**) Quantification of CD4CAR-labeled CNS tissue (gray and white matter) as a percentage of the total CNS tissue area in both CAR and control macaques (*n =* 4 macaques; 5 CNS tissues per macaque). Dotted line, threshold signal set based on average percentage of CD4CAR immunoreactivity in representative CNS sections from CAR^–^ animals (0.04%). Chart shows individual data points with the median. Scale bars: 50 μm.

Among numerous secondary tissue sites that contribute to HIV persistence, the CNS is among the least understood and most difficult to access (reviewed in refs. 39, 40). We next asked whether CD4CAR immunoreactivity was detectable in distinct sites within the CNS, including parietal cortex, hippocampus, basal ganglia, thalamus, and cerebellum (Supplemental Tables 3 and 4; refs. 41, 42). We established CAR-specific staining thresholds using control CNS tissues from macaques that received neither CD4CAR nor CD4CARΔζ (0.040% of total tissue area). We observed CD4CAR immunoreactivity in white and gray matter in both CAR and CD4CARΔζ tissues (Figure 7, A and B), and we noted signal above background in parietal cortex and hippocampus of both CAR and control macaques (Figure 7C and Supplemental Figure 4, C and D). Only CAR 2 and Control 2 contained CAR marking above the threshold in the basal ganglia, and all animals except CAR 1 had CAR marking above the threshold in the thalamus (Figure 7C and Supplemental Figure 4, C and D). Interestingly, in contrast to findings in lymphoid GCs and GIT, Control 2 consistently showed a higher frequency of CAR staining in all 4 of these CNS tissue sites, compared with the other 3 animals. The observation that CAR 2 displayed higher CAR marking frequencies compared with CAR 1 across the CNS is consistent with our previous PCR-based data (10). In the cerebellum, CAR staining frequencies were below the threshold in all 4 macaques, indicating minimal to no trafficking of gene-modified cells to this site. In agreement with our previous studies, these findings strongly suggest that HSC-derived progeny engraft and persist long-term in the brain (43).

**Figure 8:**
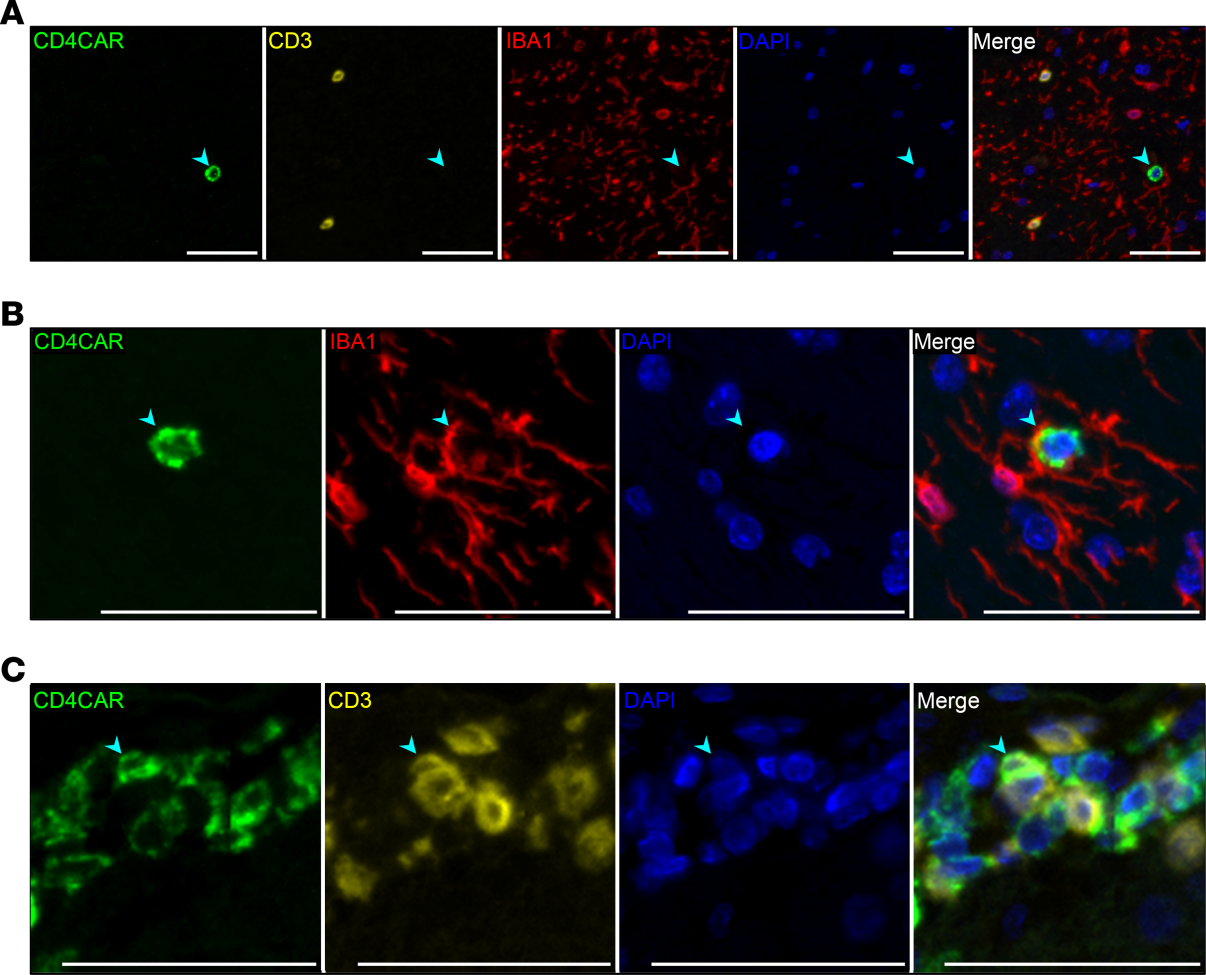
Characterization of HSPC-derived CAR^+^ cells in the CNS. Representative fluorescent mIHC photomicrographs from Control 2, illustrating the presence of HSPC-derived CD4CAR^+^ cells (arrowheads) (*n =* 4 macaques; 5 CNS tissues per macaque). (**A**) Basal ganglia: Lack of colocalization between CD4CAR (green) and CD3 (yellow) or IBA-1 (red). (**B**) Thalamus: Colocalization of CD4CAR with IBA-1, indicating a CD4CAR^+^ myeloid cell. (**C**) Hippocampus: Colocalization of CD4CAR with CD3, indicating a CD4CAR^+^ T cell. Scale bars: 50 μm.

CNS-localized CD4CAR^+^ signal was contained in phenotypically indistinct, predominantly small, round cells, ranging from 5.0 to 15.7 μm in diameter. Although CD4CAR immunoreactivity frequently did not colocalize with phenotypic markers for T cells (CD3), or resident microglia and infiltrating macrophages (IBA-1) by fluorescent mIHC (Figure 8A), we observed quantifiable colocalization in the Control 2 animal. CD4CAR colocalized with IBA-1 in 6.8%–15.8% of the total CD4CAR^+^ area across CNS tissues in this animal (Figure 8B), while CD4CAR colocalization with CD3 was observed in the hippocampus, basal ganglia, and thalamus, accounting for 0.11%–0.12% of the total CD4CAR^+^ area (Figure 8C). CD4CAR-CD20 colocalization was not observed in any section of the CNS parenchyma in either CAR or control animals, indicating minimal to no engraftment of CD4CAR^+^ B cells in the CNS. The ability of HSC-derived CAR^+^ cells to engraft in multiple CNS tissue sites raises exciting possibilities for HSC-based therapies directed at this particularly difficult-to-access viral reservoir compartment.

## Discussion

We demonstrate trafficking of HSC-derived CD4CAR^+^ cells to tissue sites known to harbor persistently HIV-1–infected cells. In particular, we observed robust engraftment of CD4CAR-modified cells within lymphoid GCs, GIT, and CNS. In addition, our data show that CAR^+^ cells actively proliferate in GCs. This systemic in vivo view of CAR localization and trafficking is unprecedented; to our knowledge, our HSC-derived CAR approach is the most efficient strategy described to target enhanced anti-HIV immune cells to these otherwise privileged sites.

All tissues evaluated in this study were collected at necropsy, nearly 2 years after HSPC transplantation and 12–17 weeks following withdrawal of suppressive ART and SHIV viral rebound. Each of the 4 animals reached study endpoint without any clinical signs of CD4CAR toxicity. Our SHIV/macaque model of HIV persistence in tissues is well established (41, 42) and served as an extremely useful platform for CD4CAR localization studies: preliminary RNAscope and DNAscope data further show that SHIV RNA^+^ and SHIV DNA^+^ cells persisted at necropsy in CAR 2 colon and Control 1 mesenteric lymph node (LN) (Supplemental Figure 10). The multilineage engraftment of HSC-derived CD4CAR^+^ cells in secondary tissue sites that we observed by IHC is consistent with our previous data (10). Of note, our previous PCR-based data show higher gene marking levels in some tissues, especially GIT and CNS, relative to our IHC data. As previously described, this is likely due to the fact that our PCR-based assays detect all lentiviral vector integrants, while IHC (and flow cytometry) methods only detect the proportion of those integrants that express detectable levels of transgene — in this case, CD4CAR (34).

Our IHC-based approach shows multilineage engraftment within distinct microanatomical structures that are relevant to HIV persistence, including lymphoid GC, CNS, and GIT. In addition to the well-established virus-directed function of CD4CAR T cells, other HSC-derived subsets, including NK cells and myeloid cells, have been shown to exert CAR-directed cytolytic activity in vitro and in vivo (44–51). CAR^+^ B cells were most abundant in lymphoid GCs of our animals. Notably, we have no evidence of CAR function in B cells in this study, and it is unknown whether CAR^+^ B cells are capable of exerting CAR-directed cytotoxic activity. B cells expressing granzyme B have been postulated to possess antiviral and early tumor immunosurveillance functions, with granzyme B^+^ B cells exerting granzyme-mediated cytolytic activity against tumor cell lines in vitro (52–56). Additionally, ZAP-70 and Lck, protein tyrosine kinases associated with TCR signal transduction through the CD3ζ domain, can be expressed in B cells, with increased ZAP-70 expression associated with BCR signaling (57, 58). Nevertheless, we have no data to suggest that CD4CAR^+^ B cells possessed cytolytic function in our study. As we have previously described (59), the predominance of gene-modified, HSC-derived B cells that we observed in lymphoid GCs could alternatively be used to deliver more B cell–relevant anti-HIV cargoes to these reservoir sites, such as broadly neutralizing antibodies. In short, the ability to generate multiple functional CAR^+^ subsets (T, NK, and myeloid cells), along with the potential to apply B cells as antibody delivery vehicles, is yet another advantage of utilizing gene-modified HSPCs for anti-HIV immunotherapy.

Our previous study characterized the function of HSPC-based CD4CAR molecules in CAR 1 and CAR 2, relative to Control 1 and Control 2, using virological and flow cytometry–based methods. We found that both CD4CAR expression in peripheral blood and control of post-ART SHIV rebound were more pronounced in CAR 2 relative to CAR 1 (10). In the present IHC-based study, we observed higher levels of tissue-localized CAR cells in CAR 1, relative to CAR 2. Our supplemental data and previous study show higher levels of infected cells in CAR 1 versus CAR 2 at these same tissue sites, as well. We and others have previously shown that the impacts of various HIV cure approaches on peripheral versus tissue sites of virus persistence may be distinct (42, 60). In this case, decreased antigen burden in secondary tissues in CAR 2 may not have supported expansion of virus-reactive CD4CAR T cells at these sites, whereas virus replication in the periphery was sufficient to support ongoing expansion of CD4CAR T cells in blood. Alternatively, the disconnect in these data sets could be related to the number of CAR-modified B cells, a subset that we presume to be immunologically inert. Although our data clearly show trafficking of gene-modified T cells to key sites of HIV persistence in both CAR 1 and CAR 2 (e.g., up to 10% localization in lymphoid GCs), these cells are vastly outnumbered by CD4CAR-expressing B cells, which constitute up to 50% of B cells in lymphoid GCs. The fact that a large/majority fraction of gene-modified cells presumably lacks antiviral function obfuscates the correlation between CAR marking levels and antiviral function. Put differently, the substantially higher level of CAR marking in CAR 1 versus CAR 2 may be due to a higher number of nonfunctional CAR-marked B cells, whereas the lower levels of CAR-marked T cells in CAR 2 may possess greater antiviral function.

We could not phenotypically identify the majority of CD4CAR^+^ cells in the CNS of our animals using our mIHC panel. This is consistent with previous murine HSPC transplantation studies, wherein subsets of HSC-derived cells in the CNS displayed variable CD45 and no IBA-1 immunoreactivity (61, 62). In contrast to our study, the majority of the HSC-derived cells in the murine CNS study were IBA-1^+^, reflecting potential species-specific differences. However, the presence of robust CD4CAR immunoreactivity with sparse IBA-1 colocalization, and the fact that CD4CAR^+^ cells in the CNS displayed a predominately round-to-ameboid morphology (as opposed to the predominately ramified cell morphology observed with IBA-1^+^ myeloid cells) (63), suggests that CD4CAR^+^ and IBA-1^+^ cells are indeed distinct. Therefore, the HSC-derived CD4CAR^+^ cell types we observed in the CNS remain to be fully characterized. Nevertheless, our findings in the CNS are consistent with previous studies from our group and others (43, 64), demonstrating that HSC-derived therapies enable trafficking of cell-based therapies to the brain, and in close proximity to potential CNS-associated viral reservoirs (65).

Surprisingly, the frequency of CD4CAR immunoreactivity in lymphoid GCs, CNS, and GIT were comparable between CD4CAR and CD4CARΔζ control animals. This finding clearly demonstrates that the localization patterns we observe are independent of CD4CAR signaling function, which is absent in the CD4CARΔζ version expressed by Control 1 and Control 2. There are 2 potential models that may explain this finding: CD3ζ-independent CAR signaling or postconditioning compartmentalization. CD3ζ-independent CAR signaling may have mediated CD4CAR^+^ cell trafficking via a glycine motif in the CD4 transmembrane domain, which is conserved in both the CD4CAR and CD4CARΔζ constructs and has been shown to play an important role in T cell activation, independent of Lck-mediated signal transduction (66). The mechanism for how this CD4CAR glycine motif or other non-CD3ζ domains may mediate T cell activation is currently unknown but may occur through dimerization with other membrane-bound proteins or to aid in proper membrane localization.

An alternative model holds that myeloablative and lymphodepleting conditioning with TBI, which was used to promote HSC engraftment in this study, leads to disruption and regeneration of various tissue sites and sequestration of HSCs and their progeny. For example, disruption of the blood brain barrier (BBB) permits influx of HSPCs, which become compartmentalized following BBB repair (43). Trafficking and reconstitution of hematopoietic cell subsets following TBI is likely to be a generalized phenomenon that is independent of a given gene modification strategy (i.e., CD4CAR). This model is consistent with previous small animal studies from our group and others, demonstrating that donor HSC–derived cells traffic to lymphoid GCs (59, 67), CNS tissues (61, 68–71), and GIT (59, 72). Lower-dose lymphodepleting chemotherapy regimens for CAR T cells are designed to activate cytokine expression in vivo to facilitate maintenance and expansion of the T cell compartment (including CAR T cells) (73, 74). Likewise, the strong proliferative pressure induced by highly lymphodepleting TBI-based conditioning likely drove expansion and trafficking of these same immune cell populations. However, to increase the safety, feasibility, and applicability of our HSC-CAR approach for otherwise healthy people living with HIV, TBI-based conditioning should be replaced with less toxic chemotherapeutic conditioning regimens (75–77), or nongenotoxic, antibody-based conditioning regimens designed to establish a robust niche for HSC engraftment, while minimizing collateral damage to other hematopoietic cells (78–81). This strategy will enable substantial retention of endogenous HIV-specific immune cell subsets, whose function in tandem with HSC-derived CAR T cells will likely be essential for any approach designed to support long-term ART-free HIV-1 remission (41, 82).

We found that between 26.2% and 66.6% of the assessed area within lymphoid GCs stained positive using our anti-CD4CAR immunoassay but did not colocalize with either lymphoid lineage markers (e.g., CD3, CD20) or myeloid markers (CD68/CD163). This likely reflects either limitations in the area analysis approach that we employed to identify colocalized proteins and/or potential downregulation of key phenotypic lineage markers, especially CD20 and CD3. Although our area analysis strategy allowed for quantitative colocalization analyses with robust specificity, this approach may be limited in identifying surface marker coexpression on the same cells. For example, if 2 markers are present close together or are physically interacting on the plasma membrane, we would expect them to be scored as colocalized. However, if CD4CAR and a given lineage marker are coexpressed but exhibit polarized expression on a single cell, area analysis could interpret this as separate lineage^+^ and CD4CAR^+^ cells, depending on the orientation of each region relative to that of the imaged section. CD4CAR could also be expressed on cells with downregulated expression of lineage markers. In addition to the well-characterized virus-dependent downregulation of T cell markers, including CD3 and CD4 (83–85), CD20 expression in B cells has been reported to be downregulated in a CD40-dependent manner (86). As CD40/CD40L binding to T follicular helper cells is critical for B cell affinity maturation within the lymphoid GC (reviewed in refs. 87, 88), CD20 may be downregulated on a subset of GC-localized CD4CAR^+^ B cells. This could explain the majority of lineage^–^ CD4CAR staining, since the majority of the CD4CAR^+^ cells in GCs are CD20^+^ B cells.

One important consideration for the introduction of CD4-based CARs into HSPCs is the potential for interactions between the CAR and endogenous MHC II, which could affect differentiation and maturation of HSC-derived CD4CAR^+^ T cells. Early developmental arrest of these cells has been reported in mice (89) but is not observed in controls transplanted with CD4CARΔζ HSPCs or in an MHC II^–/–^ background, raising the possibility that this developmental arrest is mediated by CD4CAR binding to MHC II and signaling through the CAR (89). Importantly, our findings demonstrate that CD4CAR^+^ T cells persist in both the peripheral blood and in secondary tissue sites, indicating that CD4CAR-mediated T cell developmental arrest is not occurring in our autologous, immunocompetent large animal model.

We conclude that HSPC-derived CD4CAR^+^ cells can persist long-term in diverse physiological sites without observable toxicities, underscoring a key advantage of HSPC-mediated immunotherapeutic delivery. Our data show long-term, multilineage engraftment of HSPC-derived CD4CAR^+^ cells in HIV tissue reservoirs. Implementing second-, third-, and fourth-generation CARs to improve effector function, adding latency reversing agents to reveal latently infected cells to CAR-mediated cytotoxicity, and incorporating genes encoding broadly neutralizing antibodies as a method of additive immunotherapy to block ongoing viral infection are among the numerous strategies that could be combined with HSC-derived CD4CAR cells to enable ART-free remission of HIV-1, as observed in Berlin and London. Delivery and maintenance of CAR-modified cells in these tissue sites has the potential for additional therapies outside the scope of HIV, including cancer and autoimmune disorders.

## Methods

### NHP study outline.

NHP studies were conducted as described previously (10) and are summarized in Figure 1. Briefly, juvenile male pigtail macaques were primed with granulocyte CSF and stem cell factor prior to collection of BM aspirates and enrichment of CD34^+^ HSPCs. HSPCs were transduced twice with lentiviral vectors at a multiplicity of infection of 5–10. Each animal was transplanted with autologous, CD4CAR-modified HSPCs following myeloablative conditioning (1020 cGy TBI). Two experimental animals (CAR 1 and CAR 2) were transplanted with HSPCs transduced with lentiviral vectors expressing CD4CAR. Control animals (Control 1 and Control 2) were transplanted with HSPCs that expressed CD4CARΔζ, which lacks the cytoplasmic signal transduction domain, rendering the CAR incapable of signaling when bound to antigen. Both vectors also expressed the membrane fusion inhibitor mC46 (90, 91). At least 200 days following HSPC transplantation, NHPs were infected with SHIV-1157ipd3N4 (92). ART consisted of Tenofovir (20 mg/kg, s.c.), Emtricitabine (40 mg/kg, s.c.), and Raltegravir (150 mg oral) and was initiated approximately 24 weeks after infection (Gilead Sciences, Merck). ART was withdrawn 28 weeks later, and viral rebound was monitored in each animal for approximately 15 weeks prior to necropsy. All tissue samples in the present study were collected at necropsy, following ART withdrawal and SHIV rebound. Representative tissues from macaques that did not receive either a CD4CAR or CD4CARΔζ transgene (CAR^–^) were utilized as controls. CAR^–^ animals were transplanted with HSPCs transduced with lentiviral vectors expressing only the membrane fusion inhibitor mC46 (93).

### Tissue collection and embedding.

At time of necropsy, representative lymphoid, CNS, and GIT tissues were preserved in freshly prepared 4% paraformaldehyde diluted from 32% stock solutions (Electron Microscopy Science) in DPBS (Thermo Fisher Scientific). Fixation proceeded for 24 hours at room temperature at a minimum ratio of 1:10 tissue/fixative. Tissues were then transferred to 80% ethanol and stored at 4°C for approximately 48–96 hours prior to processing and paraffin embedding.

### Brightfield IHC.

We used single label immunohistochemical staining against human CD4 to identify CD4CAR-expressing cells in NHP tissues. Paraformaldehyde-fixed, paraffin-embedded tissues were sectioned at 4 μm onto positively charged slides and baked for 1 hour at 60°C. Sections were deparaffinized in xylene and rehydrated through a graded ethanol series, followed by deionized water. Heat-induced epitope retrieval was accomplished by incubating the section in Tris-EDTA buffer (0.01M Trizma base, 0.001M EDTA, 0.05% Tween-20 [pH 9.0]) for 20 minutes at approximately 95°C–97°C in a commercial rice cooker, with an additional 20 minutes to allow the buffer to cool prior to rinsing the sections. Endogenous peroxidase activity was quenched with a 15-minute incubation of 3% hydrogen peroxide at ambient temperature. Next, we incubated the sections for 1 hour at ambient temperature in a humidified chamber with rabbit anti-CD4 antibody (clone SP35, MA1-39582, Thermo Fisher Scientific) following a 20-minute protein block at ambient temperature (Dako). The primary antibody was diluted to an appropriate working concentration with a commercial antibody diluent (Becton Dickinson). A horse anti–rabbit IgG poly-HRP antibody (MP-7401, Vector Laboratories) was utilized for secondary antibody labeling, and antigen-antibody complexes were visualized with DAB (Becton Dickinson). We counterstained the slides with hematoxylin (Dako), dehydrated through a graded ethanol series followed by xylene, and mounted with Permount mounting media (Thermo Fisher Scientific). Sections were washed for 5 minutes twice in TBST (0.02M Trizma base, 0.15M NaCl, 0.1% Tween-20 [pH 7.6]) at ambient temperature after being incubated with the 3% hydrogen peroxide, the primary antibody, and the secondary antibody. Controls consisted of sections of human tonsil and lymphoid tissues from CAR^–^ pigtail macaques, as well as sections labeled with nonspecific, isotype-matched rabbit antibody (08-6299, Invitrogen). Brightfield quantification could not be performed on tissue sections from the Control 1 Iliac LN, due to accumulations of endogenous brown pigment in the section, which interfered with the analysis. Although we sought to validate staining patterns observed with our anti-CD4 SP35 antibody clone using an independent anti-CD4 clone, our review of the literature (94, 95) suggested that the number of anti-CD4 clones that recognize the human CD4 epitope of our CAR but not endogenous NHP CD4 would be exceedingly low. Consistent with this, clone SP35 was the only antibody we tested that specifically labeled CD4CAR in NHP tissues.

### Multiplex fluorescent IHC.

Fluorescent mIHC of lymphoid, CNS, and GIT sections utilized the OPAL labeling method (Supplemental Table 3 and ref. 96). Paraformaldehyde-fixed, paraffin-embedded tissues were sectioned at 4 μm onto positively charged slides and baked for 1 hour at 60°C. We then deparaffinized the sections and stained them on a Leica BOND Rx stainer (Leica Biosystems). We used Leica Bond reagents for dewaxing (Dewax Solution, AR9222), antigen retrieval and antibody stripping (Epitope Retrieval Solution 2, AR9640), and rinsing after each step (Bond Wash Solution, AR9590). A high stringency wash was performed after the secondary and tertiary applications using high-salt TBST solution (0.05M Trizma base, 0.3M NaCl, 0.1% Tween-20 [pH 7.2-7.6]). OPAL Polymer HRP Mouse plus Rabbit (ARH1001EA, PerkinElmer) or Vector ImmPRESS HRP anti-goat polymer detection kit (MP-7405, Vector Laboratories) were used for all secondary antibody applications.

Antigen retrieval and antibody elution steps were performed at 100°C, with all other steps at ambient temperature. Endogenous peroxidase was blocked with 3% H_2_O_2_ for 8 minutes, followed by protein blocking with TCT buffer (0.05M Tris, 0.15M NaCl, 0.25% Casein, 0.1% Tween 20 [pH 7.5–7.7]) for 30 minutes. The first primary antibody (position 1, Supplemental Table 3) was applied for 60 minutes, followed by the secondary antibody application for 10 minutes and the application of the tertiary TSA-amplification reagent (OPAL fluorophores, PerkinElmer) for 10 minutes. The primary and secondary antibodies were eluted by incubation with the retrieval solution for 20 minutes before repeating the process with the second primary antibody (position 2), starting with a new application of 3% H_2_O_2_. We repeated the process until all positions for the respective panel were completed. There was no stripping step after the last position. Slides were removed from the stainer and stained with Spectral DAPI (PerkinElmer) for 3 minutes, rinsed for 5 minutes, and coverslipped with Prolong Gold Antifade reagent (Invitrogen). Slides were cured for 24 hours at room temperature prior to digital image acquisition.

### Image analysis.

Bright-field sections were scanned using an Aperio ScanScope AT Imaging System (Leica Biosystems) at 20× objective. We imported digital images into HALO software (Indica Labs) for analysis. Regions of interest (ROIs) were manually drawn around relevant areas, and the Indica Labs’ Area Quantification module (Version 1.0) was utilized to determine the percentage area of CD4CAR marking in each ROI. The software was trained to differentiate DAB^+^ staining versus DAB^–^hematoxylin^+^ staining based on threshold values for individual pixels. We batch-processed the images using these configurations. The output values (total area, DAB^+^ area, DAB^–^hematoxylin^+^ area), were used to calculate a ratio of CAR marking/tissue area within the ROIs for lymphoid and CNS tissues, as has been previously described (36). Aperio ScanScope digital images were imported into QuPath (version 0.2.0-m2), and ROI were annotated and exported to Fiji (ImageJ; NIH) (97–100). Figures were subsequently generated using FigureJ (101). We acquired representative fluorescent lymphoid and CNS images on a Vectra 3.0 Automated Imaging System at a 20× objective (PerkinElmer). Images were spectrally unmixed using PerkinElmer inForm software (PerkinElmer) and exported to Fiji (ImageJ) as multi-image tiffs for colocalization analysis (98–100). Fluorescent GIT sections were scanned at a 20× objective using an Aperio ScanScope FL Imaging System (Leica Biosystems). Aperio ScanScope digital images were imported into QuPath (version 0.2.0-m2), and regions containing GALT were annotated and exported to Fiji (ImageJ) for colocalization analysis (97–100). Individual regions were appended together to facilitate image binarization and analysis using a custom-built, Eclipse-compiled plugin compatible with ImageJ (Eclipse Foundation Inc.; plugin name: Append Images). We analyzed all fluorescent mIHC images for colocalization using a pixel-based area analysis approach. Binary images of each fluorescent channel were generated using the Ostu automatic thresholding algorithm (102). These images were reviewed by an observer, and adjustments to the thresholds were made as needed. The image binarization protocol is based on binarization methods described for automated quantitative analysis (AQUA) (103). Colocalization analysis of binary mIHC photomicrographs was completed using a custom-built plugin compatible with ImageJ (Eclipse Foundation Inc.; plugin name: Multiplex Pixel Colocalization; Supplemental Figure 11). The output comma-separated values (CSV) file contained a pixel area count for each phenotypic marker and the amount of area overlap between markers. The pixel counts for each potential outcome were used to calculate a ratio of CD4CAR marking/tissue area, as well as the percentage of CD4CAR colocalization with different phenotypic markers, where overlapping fluorescent markers in the same pixel area were considered colocalized. We determined the percentage of CAR colocalization with different phenotypic markers using the percentage of different phenotypic marker combinations within the respective tissue sites that colocalized with CD4CAR.

### SHIV RNAscope and DNAscope.

In situ hybridization of SHIV viral RNA and integrated proviral DNA was performed using the RNAscope 2.5 HD Brown Assay (Advanced Cell Diagnostics) with the SIVmac239 probe (catalog 312811) and the SIVmac239-sense probe (catalog 314071), respectively. Photomicrograph images were taken with a Nikon E800 at 20× and 40× objectives.

### Lentiviral VIS assay.

Characterization of vector integration sites (VIS) in CAR 1 and CAR 2 followed our previously published methods (104, 105) and focused on analyzing only the right LTR junctions. We captured vector-host junctions for PCR amplification and sequencing. Amplicon libraries were sequenced with an Illumina MiSeq. Sequences with a virus-host junction with the 3′ end LTR, including both the 3′-end U5 LTR DNA and ≥ 25 base host DNA (with ≥ 95% homology to the rhesus macaque genome version rheMac8), were considered true VIS read-outs. The sequence mapping and counting method was performed as described previously (104).

### Code availability.

Our custom-built, Eclipse-compiled plugins compatible with ImageJ are available at the following web addresses: https://github.com/BarberAxthelm/Append_Images (plugin name: Append Images; a0dc1d0) and https://github.com/BarberAxthelm/Multiplex_Pixel_Colocalization (plugin name: Multiplex Pixel Colocalization; 7ca8457).

### Statistics.

Data are presented as the median ± IQR. Comparison of the ratio of CD4CAR immunoreactivity to tissue area, as well as the percentage of CD4CAR colocalization with different phenotypic markers, between CAR and control animals was completed utilizing the unpaired, 2-tailed Mann-Whitney *U* test. *P* < 0.05 was considered statistically significant. All statistical analysis and data presentation were performed using GraphPad Prism version 8 (GraphPad Software).

### Study approval.

All animal studies were conducted in accordance with the *Guide for the Care and Use of Laboratory Animals* (National Academies Press, 2011) and the Public Health Assurance Policy, and they were approved by the IACUCs of the Fred Hutchinson Cancer Research Center and the University of Washington (protocol no. 3235-01). The Fred Hutchinson Cancer Research Center and the University of Washington are full AAALAC accredited institutions.

## Author contributions

IMBA, HPK, and CWP designed the study and wrote the manuscript. AZ, JAZ, SGK, HPK, and CWP designed the initial NHP study. IMBA performed the singleplex IHC staining and data analysis. IMBA and KYS performed the mIHC data analysis. VBA wrote the plugins for mIHC colocalization analysis. GWS and ISYC generated the VIS data.

## Supplementary Material

Supplemental data

## Version 1 01/11/2021

Electronic publication
