# Supplementary material for: Stem cell–derived CAR T cells traffic to HIV reservoirs in macaques
Source: JCI Insight. 2021 Jan 11;6(1):e141502. doi: 10.1172/jci.insight.141502 (PMC7821595; doi:10.1172/jci.insight.141502)
Supplement: Supplemental data [file jciinsight-6-141502-s140.pdf]

Stem cell-derived CAR-T cells traffic to HIV reservoirs in macaques

Isaac Barber-Axthelm[1,2], Valerie Barber-Axthelm[1], Kai Yin Sze[1], Anjie Zhen[3,4], Gajendra W Suryawanshi[4,5], Irvin SY Chen[3,4,5], Jerome A. Zack[3,4,5], Scott G. Kitchen[3,4], Hans-Peter Kiem[1,6,7*], Christopher W. Peterson[1,6*]

[1] Stem Cell and Gene Therapy Program, Fred Hutchinson Cancer Research Center, Seattle, WA, USA

[2] Department of Comparative Medicine, University of Washington, Seattle, Washington, USA

[3] Department of Medicine, Division of Hematology and Oncology, David Geffen School of Medicine at University of California, Los Angeles, CA, USA

[4] UCLA AIDS Institute, Los Angeles, CA, USA

[5] Department of Microbiology, Immunology and Molecular Genetics, David Geffen School of Medicine at University of California, Los Angeles, CA, USA

[6] Departments of Medicine, University of Washington, Seattle, Washington, USA

[7] Departments of Pathology, University of Washington, Seattle, Washington, USA

*Correspondence should be addressed to H.-P.K (hkiem@fhcrc.org) or C.W.P. (cwpeters@fhcrc.org).

Corresponding Authors: Hans-Peter Kiem or Christopher W. Peterson
Fred Hutchinson Cancer Research Center
1100 Fairview Ave N, Mail Stop D1-100
P.O. Box 19024
Seattle, WA 98109-1024
Phone: 206-667-4425 or 206-667-6646
Fax: 206-667-6124

ABSTRACT

Allogeneic hematopoietic stem cell transplantation (allo-HSCT) with CCR5-negative donor cells thus far is the only treatment known to cure HIV-1 in patients with underlying malignancy. This is likely due to a donor cell-mediated graft-versus-host effect targeting HIV reservoirs. Allo-HSCT would not be an acceptable therapy for most persons living with HIV due to the transplant-related side effects. Chimeric antigen receptor (CAR) immunotherapies specifically traffic to malignant lymphoid tissues (lymphomas) and in some settings are able to replace allo-HSCT. Here we quantified the engraftment of HSC-derived, virus-directed CAR T-cells within HIV reservoirs in a macaque model of HIV infection, using novel immunohistochemistry assays. HSC-derived CAR cells trafficked to and displayed multilineage engraftment within tissue-associated viral reservoirs, persisting for nearly 2 years in lymphoid germinal centers, the brain, and the gastrointestinal tract. Our findings demonstrate that HSC-derived CAR^{+} cells reside long-term and proliferate in numerous tissues relevant for HIV infection and cancer.

INTRODUCTION

Hematopoietic stem and progenitor cell (HSPC) transplantation has emerged as a promising approach to achieve a functional HIV cure, largely due to the clinical cases of the "Berlin Patient" and the "London Patient"(1, 2). Each patient received HSPCs from donors who were homozygous for a naturally occurring 32-base pair deletion in the CCR5 gene (CCR5Δ32), resulting in a truncated protein that is not expressed on the cell surface. The sustained viral remission in these patients following withdrawal of suppressive antiretroviral therapy is likely due to a combination of: i) the conditioning regimens which enabled rapid, complete donor chimerism and which may also have cleared a portion of latently infected host cells; ii) graft-versus host-mediated elimination of latently infected host cells during donor cell engraftment (frequently referred to as "Graft-versus-Virus" or "Graft-versus-Reservoir"); and, iii) near-complete replacement of the host immune system with homozygous CCR5Δ32 donor cells, conferring resistance to CCR5-tropic HIV-1 (3). While the successful treatment of these 2 patients marks a significant milestone in efforts aimed at an HIV cure, there are several limitations that render this approach infeasible for the vast majority of people living with HIV. These include significant risk of morbidity and mortality associated with allo-HSCT, the limited prevalence of CCR5Δ32 donors, and the potential for the virus to circumvent the CCR5Δ32 mutation via a CCR5- to CXCR4-tropism shift (4). We are interested in applying principles from the successful treatment of the Berlin and London patients, using alternative approaches which are comparably potent, less toxic, and applicable to a larger patient population.

Expression of HIV-specific chimeric antigen receptors (CARs) from gene-modified, autologous HSPCs has the potential to overcome each of the limitations associated with allo-HSCT. CARs containing a CD4 extracellular and transmembrane domain fused to the CD3ζ signal transduction domain ("CD4CAR") effectively redirect primary T-lymphocytes to lyse HIV infected cells in vitro (5, 6). These CAR molecules, as well as CARs based on anti-HIV broadly neutralizing antibodies (7-9) bind to the HIV Env protein expressed on the surface of infected

cells, which permits CAR-mediated target cell lysis, independent of MHC presentation and thus circumventing HIV-mediated MHC I downregulation. Previously, we reported similar impacts in nonhuman primates (NHPs) infected with simian/human immunodeficiency virus (SHIV) and transplanted with CD4CAR HSPCs, including expansion of CAR^{+} effector cells, reduced tissue viral loads, and reduced rebound plasma viremia following discontinuation of ART (10). These findings demonstrated that HSC-derived CD4CAR cells can exert an antiviral effect in vivo.

We reasoned that genetically modifying HSPCs to express CD4CAR permits continuous production of CAR T-cells that undergo normal physiologic development, circumventing the need for significant *ex vivo* expansion that is currently required for adoptively transferred CAR T-cell therapies. However, the ability of these cells to traffic to and persist in HIV reservoir tissues remains unclear. Multiple tissue sites have been identified as potential HIV reservoirs, including lymphoid germinal centers (GCs) (11-21), the CNS (16, 22-27), and the gastrointestinal tract (GIT) (16, 17, 28-33). The physiologic properties that allow these sites to function as HIV reservoirs may include one or more of the following: 1) resident cell populations that support latent viral infection and/or active viral replication after prolonged combination antiretroviral therapy; 2) resident cells that retain infectious virions for prolonged periods of time; and 3) the immune privileged nature of certain sites which exclude effector cells necessary for viral clearance. As with any approach designed to control viral replication in the absence of antiretroviral therapy (termed “functional cure” or “functional remission”), CAR^{+} cells must traffic to HIV tissue reservoir sites, engraft and persist, and recognize and kill infected cells. Here, we extended our previous evaluation of the safety and efficacy of HSC-derived, CD4CAR expressing T-lymphocytes in SHIV-infected NHP (10), focusing on tissues that were collected at study endpoint. Our objective was to quantify the trafficking and persistence of HSC-derived, CAR-expressing cells in HIV tissue reservoir sites, utilizing CD4CAR-specific IHC-based assays. We show that HSC-derived, multilineage $CD4CAR^{+}$ cells traffic to and persist long term

in lymphoid GCs, CNS tissue, and GIT tissue, and actively proliferate within these HIV reservoir sites.

RESULTS

Overview of Study Animals and Necropsy Data. The data presented here build upon a previously published experiment focused on 4 pigtail macaques that were transplanted with autologous, lentiviral vector-modified HSPCs. Two animals, referred to as CAR 1 and CAR 2, received CD4CAR-transduced HSPCs, while 2 controls (Control 1 and Control 2) received a signaling defective CAR known as CD4CARΔζ. A study schematic for this cohort is shown in **Figure 1**. Infused HSPC products contained a range of 4.7-40.0% gene-marked cells; following infusion into the total body irradiation (TBI)-conditioned autologous host, each animal recovered with typical kinetics (**Supplemental Table 1**). Approximately 28 weeks post-transplant, each animal was infected with SHIV, at which point gene marking levels were low (0.1-0.8% by flow cytometry, and 1.55%-8.79% by PCR). Integration site analyses demonstrated that gene-marked peripheral blood cells from both CAR 1 and CAR 2 displayed a polyclonal integration pattern (**Supplemental Figure 1**). All of the samples in the present study were collected at necropsy, following primary SHIV infection, ART suppression, ART release, and viral rebound (**Figure 1**). Necropsy data from our previously published work is summarized in **Supplemental Table 2** and **Supplemental Figures 2-3** (10). SHIV plasma viral loads at necropsy ranged from 999–884,846 copies/mL plasma (**Supplemental Table 2**). SHIV DNA (**Supplemental Figure 2A**) and SHIV RNA (**Supplemental Figure 2B**) were detectable, often at high levels, in a panel of 25 tissues, as well as 4-limb bone marrow and PBMC. CD4CAR gene marking levels in total PBMC ranged from 0.64-2.71% by flow cytometry and 2.72-15.63% by PCR (**Supplemental Table 2**). Higher gene marking levels by PCR are expected, due to i) the subset of integrated vector proviruses which have been silenced or accrued mutations that prevent transgene expression, and ii) the fact that >1 integration event per cell is detectable by PCR but may not be detected by flow cytometry (34). Among CD4CAR^{+} PBMC, the majority of CAR+ cells were CD20^{+} B cells, followed by CD4^{+} and CD8^{+} T cells, CD2^{+}NKG2A^{+} NK cells, and CD14^{+} monocytes (**Supplemental Figure S3**). Our prior studies demonstrated that each of the 4

animals recovered comparably following autologous HSPC transplantation, and confirmed that both SHIV-infected target cells and CD4CAR⁺ effector cells were present at end-of-study necropsy. Necropsy samples from this cohort were hence well suited to build and validate an assay to quantify the trafficking of CD4CAR⁺ cells, which was the main objective of this work.

Validation of CD4CAR immunohistochemistry assay. The CD4CAR construct utilized for these studies expresses a cell surface protein consisting of a human CD4 extracellular and transmembrane domain, fused to a human CD3ζ signal transduction domain, which has previously been tested in clinical trials and in NHPs (7, 10). We designed our IHC assay to specifically detect the human CD4 extracellular domain of CD4CAR, while minimizing background from endogenous NHP CD4 molecules. To confirm specific labeling of the human CD4 extracellular domain, we stained lymphoid tissue sections collected at necropsy from CAR 1, CAR 2, Control 1, and Control 2. We applied a monoclonal anti-CD4 antibody (clone: SP35), and evaluated for CD4CAR-specific immunoreactivity (10), which was observed in tissues from macaques that received signaling-proficient CD4CAR (CAR1 and CAR 2), and animals that received the signaling-defective CD4CARΔζ which retains the extracellular CD4 domain but lacks the CD3ζ signal transduction domain (Control 1 and Control 2) (**Figure 2A and B**). Importantly, CD4CARΔζ should still be labeled with our SP35 antibody clone, but should not facilitate intracellular signaling in response to antigen binding. No signal was observed in control samples stained with a non-specific, isotype-matched rabbit antibody (**Figure 2C and D**), Positive control sections of human tonsil showed CD4-specific immunoreactivity predominately in the paracortex, consistent with specific antibody binding to the human CD4 antigen in the CAR modified animals (**Figure 2E**). Importantly, lymphoid tissues from HSPC-transplanted pigtail macaques that did not receive CD4CAR transduced cells did not display any antigen-specific immunoreactivity (**Figure 2F**). This data shows that anti-CD4 SP35 immunoreactivity observed in tissues from CD4CAR transduced animals is due to specific CAR labeling, and not

to cross-reactivity with the endogenous macaque CD4 antigen or non-specific binding from the secondary antibody.

Multilineage engraftment of HSC-derived CAR$^+$ cells in lymphoid germinal centers. Next we applied our CD4CAR-specific IHC assay to quantify trafficking of HSC-derived, CAR$^+$ cells to lymphoid germinal centers. We stained paraffin-embedded sections from secondary lymphoid organs from 2 CD4CAR macaques ("CAR 1" and "CAR 2"), and 2 control CD4CAR$\Delta\zeta$ macaques ("Control 1" and "Control 2"), using the anti-CD4 SP35 monoclonal antibody to detect CD4CAR$^+$ cells. Tissues from macaques that did not receive CD4CAR or CD4CAR$\Delta\zeta$ were used as a threshold for CAR specific marking. We observed CD4CAR immunoreactivity in the germinal centers of all tissues in all 4 macaques (CD4CAR and CD4CAR$\Delta\zeta$) (**Figure 3A-C**). Within individual tissue sections, the amount of CD4CAR immunoreactivity ranged from absent to occupying almost the entire area of a given germinal center**.** We used a previously described brightfield IHC quantification approach (35, 36) to estimate the amount of CAR marking in germinal centers. This pixel-based area analysis demonstrated that 1.4-62.6% of the total germinal center tissue area was positive for CD4CAR protein expression. The most robust CD4CAR marking was observed in tissues from CAR 1 and both control animals (**Figure 3D and Supplemental Figure 4A**). No differences were observed in the frequency of germinal center CAR staining between CD4CAR and CD4CAR$\Delta\zeta$ animals, aside from the overall reduced CAR marking in tissues from CAR 2. However, all 4 animals had CAR marking frequencies above the threshold set by the unmodified control tissues, suggesting that HSC-derived CAR$^+$ cells traffic to lymphoid germinal centers in a manner independent of CD4CAR signaling.

Following quantification of CD4CAR localization in various secondary lymphoid tissues (**Figure 3 and Supplemental Figure 4A and 4D**), we next set out to identify the lymphoid and myeloid subsets that expressed the CAR transgene within each site (**Figure 4 and Supplemental Figure 5**). We applied a fluorescent multiplex immunohistochemistry (mIHC)

approach with pixel-based quantitation to enumerate CD4CAR$^+$ B-cells (CD20$^+$), total T-cells (CD3$^+$) Cytotoxic T Lymphocytes (CTL, CD3$^+$CD8$^+$), CD4 and double negative T-cells (CD3$^+$CD8$^-$), and monocytes/macrophages (CD68$^+$CD163$^+$) (**Supplemental Tables 3 and 4**). We could not directly stain for NHP CD4, due to cross-reactivity with CD4CAR. Between 26.2 and 66.6% of the CD4CAR$^+$ area within the lymphoid germinal centers did not colocalize with any of the phenotypic markers. Of the remaining CD4CAR$^+$ immunoreactivity, the majority colocalized with CD20, consistent with a predominance of B-cells in lymphoid germinal centers (**Figure 4A and Supplemental Figures 3, 5, and 6**). The CD4CAR marker also colocalized with CD68$^+$CD163$^+$ monocyte/macrophage subsets and CD3+ T-cells, which were further delineated into CTL and CD4$^+$/double negative T-cells (**Figure 4B**). These data demonstrate multilineage engraftment of HSC-derived CAR$^+$ cells within lymphoid GC's, most notably CD4CAR$^+$ CD3$^+$ T-cells and CAR$^+$CD3$^+$CD8$^+$ CTLs-the functional HSC-derived subset of greatest therapeutic interest (10).

HSC-derived CAR$^+$ cells actively proliferate within the lymphoid GCs. The germinal center reaction is characterized by early antigen-activated B-cell proliferation, which displaces naïve B-cells to the periphery, forming the mantel zone (37). Additionally, chronic HIV and SIV infection is associated with a period of follicular hyperplasia with marked B-cell expansion, which is hypothesized to be due to persistent viral antigen stimulation and CD4$^+$ T-cell priming (reviewed in (38)). To assess whether HSC-derived CAR$^+$ cells in GCs actively proliferated under conditions of chronic SHIV infection, we employed a fluorescent mIHC assay using Ki-67 as a marker of cellular proliferation (**Supplementary Table 3**). Ki-67$^+$CAR$^+$ cells were readily identified in germinal centers from both CD4CAR and CD4CAR$\Delta\zeta$ macaques. There was no qualitative difference in the level of Ki-67$^+$CAR$^+$ cells between CD4CAR and CD4CAR$\Delta\zeta$ animals, aside from the reduced frequency of CAR marking observed in tissues from CAR 2 (**Supplemental Figure 7A and Figure 3B**). Consistent with our phenotypic analyses, Ki-

67$^+$CAR$^+$ cells were predominately CD20$^+$ B-cells (**Supplemental Figure 7B**). Our results show that CD4CAR modification of GC B-cells does not hinder proliferative responses, with the relative abundance of Ki-67$^+$ GC B-cells likely reflecting lymphoid hyperplasia associated with chronic SHIV infection.

Multilineage engraftment of HSC-derived CAR$^+$ cells in GIT. Next, we evaluated HSC-derived CD4CAR$^+$ cell trafficking and differentiation in 6 representative gastrointestinal lymphoid tissues (duodenum, jejunum, ileum, cecum, colon, rectum)(**Supplementary Tables 3 and 4**). The ratio of CAR marking to total tissue area was determined by quantifying the amount of CAR marking using threshold fluorescent images, then dividing by the amount of threshold CAR and DAPI signal in the identical images. Tissues from macaques that did not receive a CAR had 0.007% average CAR marking, which we used as the threshold for CAR specific marking as described above. CAR specific immunoreactivity above threshold levels was observed in all tissue sites from CAR 1 and Control 1 (**Figure 5A-C**), and in all Control 2 tissues except the duodenum. In CAR 2, marking was observed in all tissue sites except the cecum. CAR marking could not be quantified in rectal tissue from Control 1, because of a lack of gut-associated lymphoid tissue (GALT) in the sections that we evaluated. Consistent with our observations in lymphoid GCs (**Figures 3B and 4A**), CAR 2 consistently had lower frequencies of CAR immunoreactivity compared to the other 3 animals in all GIT tissue sites (**Figure 5D and Supplemental Figure 4B and 4D**). We evaluated the phenotypic distribution of CD4CAR$^+$ cells in GIT via fluorescent mIHC, focusing on CD20 and CD3 markers to identify B-cells and T-cells, respectively. Between 5.4-58.4% of the CD4CAR$^+$ area did not colocalize with CD3 or CD20. The majority of the remaining CD4CAR immunoreactive area colocalized with CD3, indicating a predominance of CAR$^+$ T-cells in the GALT and lamina propria, whereas a smaller proportion of CD4CAR signal colocalized with CD20 (**Figure 6A and B, Supplemental Figure 8, and Supplemental Figure 9**). These data demonstrate multilineage engraftment of HSC-derived

CD4CAR⁺ cells within the small and large intestine. Importantly, the majority of phenotypically quantifiable CD4CAR⁺ cells were CD3⁺ T-cells, the functional subset of greatest therapeutic interest for CD4CAR-based clearance of persistently HIV/SHIV-infected targets.

Trafficking of HSC-derived CAR⁺ cells to CNS tissues. Among numerous secondary tissue sites that contribute to HIV persistence, the CNS is among the least understood and most difficult to access (reviewed in (39, 40)). We next asked whether CD4CAR immunoreactivity was detectable in distinct sites within the CNS, including parietal cortex, hippocampus, basal ganglia, thalamus, and cerebellum (**Supplementary Tables 3 and 4**) (41, 42). We established CAR specific staining thresholds using control CNS tissues from macaques that received neither CD4CAR nor CD4CARΔζ (0.040% of total tissue area). We observed CD4CAR immunoreactivity in white and gray matter in both CAR and CD4CARΔζ tissues (**Figure 7A and B**), and noted signal above background in parietal cortex and hippocampus of both CAR and control macaques (**Figure 7C and Supplemental Figure 4C-4D**). Only CAR2 and Control 2 contained CAR marking above the threshold in the basal ganglia, and all animals except CAR 1 had CAR marking above the threshold in the thalamus (**Figure 7C and Supplemental Figure 4C-4D**). Interestingly, in contrast to findings in lymphoid GCs and GIT, Control 2 consistently showed a higher frequency of CAR staining in all 4 of these CNS tissue sites, compared to the other 3 animals. The observation that CAR 2 displayed higher CAR marking frequencies compared to CAR 1 across the CNS is consistent with our previous PCR-based data (10). In the cerebellum, CAR staining frequencies were below the threshold in all 4 macaques, indicating minimal to no trafficking of gene modified cells to this site. In agreement with our previous studies, these findings strongly suggest that HSC-derived progeny engraft and persist long term in the brain (43).

CNS-localized CD4CAR⁺ signal was contained in phenotypically indistinct, predominantly small, round cells, ranging from 5.0-15.7 μm in diameter. Although CD4CAR

immunoreactivity frequently did not colocalize with phenotypic markers for T-cells (CD3), or resident microglia and infiltrating macrophages (IBA1) by fluorescent mIHC (**Figure 8A**), we observed quantifiable colocalization in the Control 2 animal. CD4CAR colocalized with IBA1 in 6.8-15.8% of the total CD4CAR$^+$ area across CNS tissues in this animal (**Figure 8B**), while CD4CAR colocalization with CD3 was observed in the hippocampus, basal ganglia, and thalamus, accounting for 0.11-0.12% of the total CD4CAR$^+$ area (**Figure 8C**). CD4CAR-CD20 colocalization was not observed in any section of the CNS parenchyma in either CAR or control animals, indicating minimal to no engraftment of CD4CAR$^+$ B-cells in the CNS. The ability of HSC-derived CAR$^+$ cells to engraft in multiple CNS tissue sites raises exciting possibilities for HSC-based therapies directed at this particularly difficult-to-access viral reservoir compartment.

DISCUSSION

We demonstrate trafficking of HSC-derived CD4CAR⁺ cells to tissue sites known to harbor persistently HIV-1 infected cells. In particular, we observed robust engraftment of CD4CAR-modified cells within lymphoid GCs, GIT and CNS. In addition, our data show that CAR⁺ cells actively proliferate in germinal centers. This systemic in vivo view of CAR localization and trafficking is unprecedented; to our knowledge, our HSC-derived CAR approach is the most efficient strategy yet described to target enhanced anti-HIV immune cells to these otherwise privileged sites.

All tissues evaluated in this study were collected at necropsy, nearly 2 years post-HSPC transplantation and 12-17 weeks following withdrawal of suppressive ART and SHIV viral rebound. Each of the 4 animals reached study endpoint without any clinical signs of CD4CAR toxicity. Our SHIV/macaque model of HIV persistence in tissues is well established (41, 42), and served as an extremely useful platform for CD4CAR localization studies: preliminary RNAscope and DNAscope data further show that SHIV RNA+ and SHIV DNA+ cells persisted at necropsy in CAR 2 colon and Control 1 mesenteric lymph node (**Supplemental Figure 10**). The multilineage engraftment of HSC-derived CD4CAR⁺ cells in secondary tissue sites that we observed by IHC is consistent with our previous data (10). Of note, our previous PCR-based data showed higher gene marking levels in some tissues, especially GIT and CNS, relative to our IHC data. As previously described, this is likely due to the fact that our PCR-based assays detect all lentiviral vector integrants, while IHC (and flow cytometry) methods only detect the proportion of those integrants that express detectable levels of transgene, in this case CD4CAR (34).

Our IHC-based approach shows multilineage engraftment within distinct microanatomical structures that are relevant to HIV persistence, including lymphoid GC, CNS and GIT. In addition to the well-established virus-directed function of CD4CAR T-cells, other HSC-derived subsets, including natural killer cells and myeloid cells have been shown to exert

CAR-directed cytolytic activity in vitro and in vivo (44-51). CAR$^+$ B-cells were most abundant in lymphoid GCs of our animals. Notably, we have no evidence of CAR function in B-cells in this study, and it is unknown whether CAR$^+$ B-cells are capable of exerting CAR-directed cytotoxic activity. B-cells expressing granzyme B have been postulated to possess antiviral and early tumor immunosurveillance functions, with granzyme B$^+$ B-cells exerting granzyme-mediated cytolytic activity against tumor cell lines in vitro (52-56). Additionally, ZAP-70 and Lck, protein tyrosine kinases associated with TCR signal transduction through the CD3ζ domain, can be expressed in B-cells, with increased ZAP-70 expression associated with BCR signaling (57, 58). Nevertheless, we have no data to suggest that CD4CAR$^+$ B-cells possessed cytolytic function in our study. As we have previously described (59), the predominance of gene-modified, HSC-derived B-cells that we observed in lymphoid GCs could alternatively be used to deliver more B-cell relevant anti-HIV cargoes to these reservoir sites, such as broadly neutralizing antibodies. In short, the ability to generate multiple functional CAR$^+$ subsets (T-, NK, and myeloid cells) along with the potential to apply B-cells as antibody delivery vehicles is yet another advantage of utilizing gene modified HSPCs for anti-HIV immunotherapy.

Our previous study characterized the function of HSPC-based CD4CAR molecules in CAR 1 and CAR 2, relative to Control 1 and Control 2, using virological and flow cytometry-based methods. We found that both CD4CAR expression in peripheral blood and control of post-ART SHIV rebound were more pronounced in CAR 2 relative to CAR 1 (10). In the present IHC-based study, we observed higher levels of tissue-localized CAR cells in CAR 1, relative to CAR 2. Our supplemental data and previous study show higher levels of infected cells in CAR 1 vs. CAR 2 at these same tissue sites as well. We and others have previously shown that the impacts of various HIV cure approaches on peripheral vs. tissue sites of virus persistence may be distinct (42, 60). In this case, decreased antigen burden in secondary tissues in CAR 2 may not have supported expansion of virus-reactive CD4CAR T cells at these sites, whereas virus replication in the periphery was sufficient to support ongoing expansion of CD4CAR T cells in

blood. Alternatively, the disconnect in these data sets could be related to the number of CAR-modified B cells, a subset which we presume to be immunologically inert. Although our data clearly show trafficking of gene-modified T cells to key sites of HIV persistence in both CAR 1 and CAR 2 (e.g. up to 10% localization in lymphoid GCs), these cells are vastly outnumbered by CD4CAR-expressing B cells, which constitute up to 50% of B cells in lymphoid GCs. The fact that a large/majority fraction of gene-modified cells presumably lacks antiviral function obfuscates the correlation between CAR marking levels and antiviral function. Put differently, the substantially higher level of CAR marking in CAR 1 vs. CAR 2 may be due to a higher number of nonfunctional CAR-marked B cells, whereas the lower levels of CAR-marked T cells in CAR2 may possess greater antiviral function.

We could not phenotypically identify the majority of $CD4CAR^+$ cells in the CNS of our animals using our mIHC panel. This is consistent with previous murine HSPC transplantation studies, wherein subsets of HSC-derived cells in the CNS displayed variable CD45 and no IBA-1 immunoreactivity (61, 62). In contrast to our study, the majority of the HSC-derived cells in the murine CNS study were $IBA\text{-}1^+$, reflecting potential species-specific differences. However, the presence of robust CD4CAR immunoreactivity with sparse IBA-1 colocalization, and the fact that $CD4CAR^+$ cells in the CNS displayed a predominately round to ameboid morphology (as opposed to the predominately ramified cell morphology observed with $IBA\text{-}1^+$ myeloid cells) (63), suggests that $CD4CAR^+$ and $IBA\text{-}1^+$ cells are indeed distinct. As such, the HSC-derived $CD4CAR^+$ cell type(s) we observed in the CNS remain to be fully characterized. Nevertheless, our findings in the CNS are consistent with previous studies from our group and others (43, 64), demonstrating that HSC-derived therapies enable trafficking of cell-based therapies to the brain, and in close proximity to potential CNS-associated viral reservoirs (65).

Surprisingly, the frequency of CD4CAR immunoreactivity in lymphoid GCs, CNS, and GIT were comparable between CD4CAR and CD4CARΔζ control animals. This finding clearly demonstrates that the localization patterns we observe are independent of CD4CAR signaling

function, which is absent in the CD4CARΔζ version expressed by Control 1 and Control 2. There are two potential models that may explain this finding: CD3ζ-independent CAR signaling or post-conditioning compartmentalization. CD3ζ-independent CAR signaling may have mediated $CD4CAR^+$ cell trafficking via a glycine motif in the CD4 transmembrane domain, which is conserved in both the CD4CAR and CD4CARΔζ constructs, and has been shown to play an important role in T-cell activation, independent of Lck-mediated signal transduction (66). The mechanism for how this CD4CAR glycine motif or other non-CD3ζ domains may mediate T-cell activation is currently unknown but may occur through dimerization with other membrane-bound proteins, or to aid in proper membrane localization.

An alternative model holds that myeloablative and lymphodepleting conditioning with total body irradiation (TBI), which was used to promote HSC engraftment in this study, leads to disruption and regeneration of various tissue sites and sequestration of HSCs and their progeny. For example, disruption of the blood brain barrier (BBB) permits influx of HSPCs, which become compartmentalized following BBB repair (43). Trafficking and reconstitution of hematopoietic cell subsets following TBI is likely to be a generalized phenomenon that is independent of a given gene modification strategy (i.e. CD4CAR). This model is consistent with previous small animal studies from our group and others, demonstrating that donor HSC-derived cells traffic to lymphoid GCs (59, 67), CNS tissues (61, 68-71), and GIT (59, 72). Lower dose lymphodepleting chemotherapy regimens for CAR T cells are designed to activate cytokine expression in vivo to facilitate maintenance and expansion of the T cell compartment (including CAR T cells) (73, 74). Likewise, the strong proliferative pressure induced by highly lymphodepleting TBI-based conditioning likely drove expansion and trafficking of these same immune cell populations. However, to increase the safety, feasibility, and applicability of our HSC-CAR approach for otherwise healthy persons living with HIV, TBI-based conditioning should be replaced with less toxic chemotherapeutic conditioning regimens (75-77), or nongenotoxic, antibody-based conditioning regimens designed to establish a robust niche for

HSC engraftment while minimizing collateral damage to other hematopoietic cells (78-81). This strategy will enable substantial retention of endogenous HIV-specific immune cell subsets, whose function in tandem with HSC-derived CAR T cells will likely be essential for any approach designed to support long-term ART-free HIV-1 remission (41, 82).

We found that between 26.2 and 66.6% of the assessed area within lymphoid germinal centers stained positive using our anti-CD4CAR immunoassay, but did not colocalize with either lymphoid lineage markers (e.g. CD3, CD20) or myeloid markers (CD68/CD163). This likely reflects either limitations in the area analysis approach that we employed to identify colocalized proteins, and/or potential downregulation of key phenotypic lineage markers, especially CD20 and CD3. Although our area analysis strategy allowed for quantitative colocalization analyses with robust specificity, this approach may be limited in identifying surface marker co-expression on the same cells. For example, if two markers are present close together or are physically interacting on the plasma membrane, we would expect them to be scored as colocalized. However, if CD4CAR and a given lineage marker are co-expressed but exhibit polarized expression on a single cell, area analysis could interpret this as separate lineage$^+$ and CD4CAR$^+$ cells, depending on the orientation of each region relative to that of the imaged section. CD4CAR could also be expressed on cells with downregulated expression of lineage markers. In addition to the well-characterized virus-dependent downregulation of T cell markers including CD3 and CD4 (83-85), CD20 expression in B-cells has been reported to be downregulated in a CD40-dependent manner (86). As CD40/CD40L binding to T follicular helper cells is critical for B-cell affinity maturation within the lymphoid GC (Reviewed in (87, 88)), CD20 may be downregulated on a subset of GC-localized CD4CAR$^+$ B cells. This could explain the majority of lineage-negative CD4CAR staining, since the majority of the CD4CAR$^+$ cells in GCs are CD20$^+$ B-cells.

One important consideration for the introduction of CD4-based CARs into HSPCs is the potential for interactions between the CAR and endogenous MHC II, which could affect

differentiation and maturation of HSC-derived CD4CAR$^+$ T-cells. Early developmental arrest of these cells has been reported in mice (89), but is not observed in controls transplanted with CD4CARΔζ HSPCs or in an MHC II$^{-/-}$ background, raising the possibility that this developmental arrest is mediated by CD4CAR binding to MHC II and signaling through the CAR (89). Importantly, our findings demonstrate that CD4CAR$^+$ T-cells persist in both the peripheral blood and in secondary tissue sites, indicating that CD4CAR mediated T-cell developmental arrest is not occurring in our autologous, immunocompetent large animal model.

We conclude that HSPC-derived CD4CAR$^+$ cells can persist long-term in diverse physiological sites without observable toxicities, underscoring a key advantage of HSPC-mediated immunotherapeutic delivery. Our data show long-term, multilineage engraftment of HSPC-derived CD4CAR$^+$ cells in HIV tissue reservoirs. Implementing 2nd, 3rd, and 4th generation CARs to improve effector function, adding latency reversing agents to reveal latently infected cells to CAR-mediated cytotoxicity, and incorporating genes encoding broadly-neutralizing antibodies as a method of additive immunotherapy to block ongoing viral infection are among the numerous strategies that could be combined with HSC-derived CD4CAR cells to enable ART-free remission of HIV-1 as observed in Berlin and London. Delivery and maintenance of CAR-modified cells in these tissue sites has the potential for additional therapies outside the scope of HIV, including cancer and autoimmune disorders.

MATERIALS AND METHODS

Nonhuman primate study outline. NHP studies were conducted as described previously (10), and are summarized in **Figure 1**. Briefly, juvenile male pigtail macaques were primed with granulocyte colony stimulating factor and stem cell factor prior to collection of bone marrow aspirates and enrichment of $CD34^+$ HSPCs. HSPCs were transduced twice with lentiviral vectors at a multiplicity of infection of 5-10. Each animal was transplanted with autologous, CD4CAR-modified HSPCs following myeloablative conditioning (1020 cGy total body irradiation). Two experimental animals (CAR 1 and CAR 2) were transplanted with HSPCs transduced with lentiviral vectors expressing CD4CAR. Control animals (Control 1 and Control 2) were transplanted with HSPCs that expressed CD4CARΔζ, which lacks the cytoplasmic signal transduction domain, rendering the CAR incapable of signaling when bound to antigen. Both vectors also expressed the membrane fusion inhibitor mC46 (90, 91). At least 200 days following HSPC transplantation, NHPs were infected with SHIV-1157ipd3N4 (92). ART consisted of Tenofovir (20mg/kg subcutaneous), Emtricitabine (40mg/kg subcutaneous), and Raltegravir (150mg oral), and was initiated approximately 24 weeks post-infection. ART was withdrawn 28 weeks later, and viral rebound was monitored in each animal for approximately 15 weeks prior to necropsy. All tissue samples in the present study were collected at necropsy, following ART withdrawal and SHIV rebound. Representative tissues from macaques that did not receive either a CD4CAR or CD4CARΔζ transgene ("CAR negative") were utilized as controls. CAR negative animals were transplanted with HSPCs transduced with lentiviral vectors expressing only the membrane fusion inhibitor mC46 (93).

Tissue collection and embedding. At time of necropsy, representative lymphoid, CNS, and GIT tissues were preserved in freshly-prepared 4% paraformaldehyde diluted from 32% stock solutions (Electron Microscopy Science, Hatfield, PA) in DPBS (Thermo Fisher Scientific, Waltham, MA). Fixation proceeded for 24 hours at room temperature at a minimum ratio of 1:10

tissue: fixative. Tissues were then transferred to 80% ethanol and stored at 4°C for approximately 48-96 hours prior to processing and paraffin embedding.

Brightfield immunohistochemistry. We used single label immunohistochemical staining against human CD4 to identify CD4CAR expressing cells in NHP tissues. Paraformaldehyde-fixed, paraffin-embedded tissues were sectioned at 4μm onto positively charged slides and baked for 1 hour at 60°C. Sections were deparaffinized in xylene and rehydrated through a graded ethanol series followed by deionized water. Heat-induced epitope retrieval was accomplished by incubating the section in Tris-EDTA buffer (0.01M Trizma base, 0.001M EDTA, 0.05% Tween-20 [pH 9.0]) for 20 minutes at approximately 95°C- 97°C in a commercial rice cooker, with an additional 20 minutes to allow the buffer to cool prior to rinsing the sections. Endogenous peroxidase activity was quenched with a 15-minute incubation of 3% hydrogen peroxide at ambient temperature. Next, we incubated the sections for 1 hour at ambient temperature in a humidified chamber with rabbit anti-CD4 antibody (clone: SP35, MA1-39582, Thermo Fisher Scientific, Waltham, MA) following a 20-minute protein block at ambient temperature (Dako, Carpinteria, CA). The primary antibody was diluted to an appropriate working concentration with a commercial antibody diluent (Becton Dickinson, Franklin Lakes, NJ). A horse anti-rabbit IgG poly-HRP antibody (MP-7401, Vector Laboratories, Burlingame, CA) was utilized for secondary antibody labeling, and antigen-antibody complexes were visualized with 3,3′-Diaminobenzidine (DAB) (Becton Dickinson, Franklin Lakes, NJ). We counterstained the slides with hematoxylin (Dako, Carpinteria, CA), dehydrated through a graded ethanol series followed by xylene, and mounted with Permount mounting media (Fisher Scientific, Hampton, NH). Sections were washed for 5 minutes twice in TBST (0.02M Trizma base, 0.15M NaCl, 0.1% Tween-20 [pH 7.6]) at ambient temperature after being incubated with the 3% hydrogen peroxide, the primary antibody, and the secondary antibody. Controls consisted of sections of human tonsil and lymphoid tissues from CAR negative pigtail

macaques, as well as sections labeled with non-specific, isotype-matched rabbit antibody (08-6299, Invitrogen, Carlsbad, CA). Brightfield quantification could not be performed on tissue sections from the Control 1 Iliac LN, due to accumulations of endogenous brown pigment in the section, which interfered with the analysis. Although we sought to validate staining patterns observed with our anti-CD4 SP35 antibody clone using an independent anti-CD4 clone, our review of the literature (94, 95) suggested that the number of anti-CD4 clones that recognize the human CD4 epitope of our CAR but not endogenous NHP CD4 would be exceedingly low. Consistent with this, clone SP35 was the only antibody we tested that specifically labeled CD4CAR in NHP tissues.

Multiplex fluorescent immunohistochemistry. Fluorescent multiplex IHC (mIHC) of lymphoid, CNS, and GIT sections utilized the OPAL labeling method (**Supplementary Table 3**) (96). Paraformaldehyde-fixed, paraffin-embedded tissues were sectioned at 4μm onto positively charged slides and baked for 1 hour at 60°C. We then deparaffinized the sections and stained them on a Leica BOND Rx stainer (Leica Biosystems, Wetzlar, Germany). We used Leica Bond reagents for dewaxing (Dewax Solution; AR9222), antigen retrieval and antibody stripping (Epitope Retrieval Solution 2; AR9640), and rinsing after each step (Bond Wash Solution; AR9590). A high stringency wash was performed after the secondary and tertiary applications using high-salt TBST solution (0.05M Trizma base, 0.3M NaCl, 0.1% Tween-20 [pH 7.2-7.6]). OPAL Polymer HRP Mouse plus Rabbit (ARH1001EA, PerkinElmer, Hopkington, MA) or Vector Immpress HRP anti-goat polymer detection kit (MP-7405, Vector Laboratories, Burlingame, CA) were used for all secondary antibody applications.

Antigen retrieval and antibody elution steps were performed at 100°C, with all other steps at ambient temperature. Endogenous peroxidase was blocked with 3% H_2O_2 for 8 minutes, followed by protein blocking with TCT buffer (0.05M Tris, 0.15M NaCl, 0.25% Casein, 0.1% Tween 20 [pH 7.5-7.7]) for 30 minutes. The first primary antibody (Position 1,

Supplementary Table 3) was applied for 60 minutes, followed by the secondary antibody application for 10 minutes and the application of the tertiary TSA-amplification reagent (OPAL fluorophores; PerkinElmer, Hopkington, MA) for 10 minutes. The primary and secondary antibodies were eluted by incubation with the retrieval solution for 20 minutes before repeating the process with the second primary antibody (Position 2), starting with a new application of 3% H_2O_2. We repeated the process until all positions for the respective panel were completed. There was no stripping step after the last position. Slides were removed from the stainer and stained with Spectral DAPI (PerkinElmer, Hopkington, MA) for 3 minutes, rinsed for 5 minutes, and coverslipped with Prolong Gold Antifade reagent (Invitrogen/Life Technologies, Grand Island, NY). Slides were cured for 24 hours at room temperature prior to digital image acquisition.

Image analysis. Brightfield sections were scanned using an Aperio ScanScope AT Imaging System (Leica Biosystems, Wetzlar, Germany) at 20x objective. We imported digital images into HALO software (Indica Labs, Albuquerque, NM) for analysis. Regions of interest (ROIs) were manually drawn around relevant areas, and the Indica Labs' Area Quantification module (Version 1.0) was utilized to determine the percentage area of CD4CAR marking in each ROI. The software was trained to differentiate DAB-positive staining versus DAB-negative hematoxylin-positive staining based on threshold values for individual pixels. We batch-processed the images using these configurations. The output values (Total area, DAB-positive area, DAB-negative hematoxylin-positive area), were used to calculate a ratio of CAR marking-to-tissue area within the ROIs for lymphoid and CNS tissues, as has been previously described (36). Aperio ScanScope digital images were imported into QuPath (version 0.2.0-m2), and regions of interest were annotated and exported to Fiji (ImageJ) (97-100). Figures were subsequently generated using FigureJ (101). We acquired representative fluorescent lymphoid and CNS images on a Vectra 3.0 Automated Imaging System at a 20x objective (PerkinElmer,

Hopkington, MA). Images were spectrally unmixed using PerkinElmer inForm software (PerkinElmer, Hopkington, MA) and exported to Fiji (ImageJ) as multi-image TIFFs for colocalization analysis (98-100). Fluorescent GIT sections were scanned at a 20x objective using an Aperio ScanScope FL Imaging System (Leica Biosystems, Wetzlar, Germany). Aperio ScanScope digital images were imported into QuPath (version 0.2.0-m2), and regions containing GALT were annotated and exported to Fiji (ImageJ) for colocalization analysis (97-100). Individual regions were appended together to facilitate image binarization and analysis using a custom-built, Eclipse-compiled plugin compatible with ImageJ (Eclipse Foundation, Inc. Ottawa, Ontario, Canada), plugin name: Append Images. We analyzed all fluorescent mIHC images for colocalization using a pixel-based area analysis approach. Binary images of each fluorescent channel were generated using the Ostu automatic thresholding algorithm (102). These images were reviewed by an observer, and adjustments to the thresholds were made as needed. The image binarization protocol is based on binarization methods described for Automated Quantitative Analysis (AQUA) (103). Colocalization analysis of binary mIHC photomicrographs was completed using a custom-built plugin compatible with ImageJ (Eclipse Foundation, Inc. Ottawa, Ontario, Canada), plugin name: Multiplex Pixel Colocalization (**Supplemental Figure 11**). The output comma-separated values (CSV) file contained a pixel area count for each phenotypic marker and the amount of area overlap between markers. The pixel counts for each potential outcome were used to calculate a ratio of CD4CAR marking-to-tissue area, as well as the percentage of CD4CAR colocalization with different phenotypic markers, where overlapping fluorescent markers in the same pixel area were considered colocalized. We determined the percentage of CAR colocalization with different phenotypic markers using the percentage of different phenotypic marker combinations within the respective tissue sites that colocalized with CD4CAR.

SHIV RNAscope and DNAscope. *In situ* hybridisation of SHIV viral RNA and integrated proviral DNA was performed using the RNAscope 2.5 HD Brown Assay (Advanced Cell Diagnostics) with the SIVmac239 probe (Cat# 312811) and the SIVmac239-sense probe (Cat# 314071) respectively. Photomicrograph images were taken with a Nikon E800 at 20x and 40x objectives.

Lentiviral Vector Integration Site Assay. Characterization of vector integration sites in CAR 1 and CAR 2 followed our previously published methods (104, 105) and focused on analyzing only the right LTR junctions. We captured vector-host junctions for PCR amplification and sequencing. Amplicon libraries were sequenced with an Illumina MiSeq. Sequences with a virus-host junction with the 3’ end LTR, including both the 3’-end U5 LTR DNA and ≥ 25 base host DNA (with ≥ 95% homology to the rhesus macaque genome version rheMac8), were considered true VIS read-outs. The sequence mapping and counting method was performed as described previously (104).

Statistical analysis. Data is presented as the median +/- the interquartile range. Comparison of the ratio of CD4CAR immunoreactivity to tissue area, as well as the percentage of CD4CAR co-localization with different phenotypic markers, between CAR and Control animals was completed utilizing the unpaired, two-tailed Mann-Whitney test. P value <0.05 was considered statistically significant. All statistical analysis and data presentation was performed using GraphPad Prism version 8 (GraphPad Software, La Jolla, CA).

Data availability statement. The data that support the findings of this study are available from the corresponding author upon reasonable request.

Code availability statement. Our custom-built, Eclipse-compiled plugins compatible with ImageJ are available at the following web addresses: https://github.com/BarberAxthelm/Append_Images (Plugin Name: Append Images) and https://github.com/BarberAxthelm/Multiplex_Pixel_Colocalization (Plugin Name: Multiplex Pixel Colocalization). Further information is included in the Methods section under "Image Analysis."

Study Approval. All animal studies were conducted in accordance with the Guide for the Care and Use of Laboratory Animals and the Public Health Assurance Policy, and were approved by the Institutional Animal Care and Use Committees of the Fred Hutchinson Cancer Research Center and the University of Washington (Protocol # 3235-01). The Fred Hutchinson Cancer Research Center and the University of Washington are full AAALAC accredited institutions.

AUTHOR CONTRIBUTIONS

I.B.A., H.-P.K., and C.W.P. designed the study and wrote the manuscript. A.Z., J.A.Z., S.G.K., H.-P.K., and C.W.P. designed the initial NHP study. I.B.A. performed the singleplex IHC staining and data analysis. I.B.A. and K.S. performed the mIHC data analysis. V.B.A. wrote the plugins for mIHC colocalization analysis. GWS and ISYC generated the vector integration site (IS) data.

ACKNOWLEDGEMENTS

The authors thank Helen Crawford and Joan Vermeulen for assistance in preparing this manuscript; Veronica Nelson and Erica Curry for outstanding support in our pigtail macaque studies; Robert Murnane, Donald Sodora, Brian Iritani, and Michael Axthelm for their advice on multiplex immunohistochemistry panel design, and Charles Frevert and Stephen Hewitt for their

advice on identifying a CD4-CAR specific IHC antibody. This study was supported by funds from amfAR ARCHE Awards (108688-54-RGRL, 108929-56-RGRL to SGK), NIH U19 and UM1 grants (AI117941 to SGK and AI126623 and HL129902 and U19 HL156247 to HPK), and a California Institute of Regenerative Medicine (CIRM) Quest Discovery grant (DISC2-10748 to SGK). AZ, GWS, ISYC, JAZ, and SGK were supported by grants from the James B. Pendleton Charitable Trust and the McCarthy Family Foundation. All primate work was completed at the Washington National Primate Research Center (WaNPRC), which is supported by U42 (U42OD011123) and P51 (P51OD010425) grants through the NIH Office of Research Infrastructure Programs (ORIP). All multiplex immunohistochemistry staining, slide scanning, and brightfield quantitative analysis were completed by the Experimental Histopathology Shared Resource of the Fred Hutch/University of Washington Cancer Consortium. This consortium is supported by a P30 Cancer Center Support Grant through NCI (P30 CA015704).

CONFLICTS OF INTEREST

SK and JAZ are co-founders of CDR3 therapeutics

REFERENCES

as of 09-11-2020

1. Hutter G, Nowak D, Mossner M, Ganepola S, Mussig A, Allers K, Schneider T, Hofmann J, Kucherer C, Blau O, et al. Long-term control of HIV by CCR5 Delta32/Delta32 stem-cell transplantation. *N Engl J Med.* 2009;360(7):692-8.
2. Gupta RK, Abdul-Jawad S, McCoy LE, Mok HP, Peppa D, Salgado M, Martinez-Picado J, Nijhuis M, Wensing AMJ, Lee H, et al. HIV-1 remission following CCR5Delta32/Delta32 haematopoietic stem-cell transplantation. *Nature.* 2019.
3. Peterson CW, and Kiem HP. Lessons from London and Berlin: Designing a scalable gene therapy approach for HIV cure (Invited Commentary). *Cell Stem Cell.* 2019;24(5):685-7.
4. Kordelas L, Verheyen J, Beelen DW, Horn PA, Heinold A, Kaiser R, Trenschel R, Schadendorf D, Dittmer U, Esser S, et al. Shift of HIV tropism in stem-cell transplantation with CCR5 Delta32 mutation. *N Engl J Med.* 2014;371(9):880-2.
5. Roberts MR, Qin L, Zhang D, Smith DH, Tran AC, Dull TJ, Groopman JE, Capon DJ, Byrn RA, and Finer MH. Targeting of human immunodeficiency virus-infected cells by CD8+ T lymphocytes armed with universal T-cell receptors. *Blood.* 1994;84(9):2878-89.
6. Yang OO, Tran AC, Kalams SA, Johnson RP, Roberts MR, and Walker BD. Lysis of HIV-1-infected cells and inhibition of viral replication by universal receptor T cells. *Proc Natl Acad Sci U S A.* 1997;94(21):11478-83.
7. Scholler J, Brady TL, Binder-Scholl G, Hwang WT, Plesa G, Hege KM, Vogel AN, Kalos M, Riley JL, Deeks SG, et al. Decade-long safety and function of retroviral-modified chimeric antigen receptor T cells. *Sci Transl Med.* 2012;4(132):132ra53.

8. Hale M, Lee B, Honaker Y, Leung WH, Grier AE, Jacobs HM, Sommer K, Sahni J, Jackson SW, Scharenberg AM, et al. Homology-directed recombination for enhanced engineering of chimeric antigen receptor T cells. *Mol Ther Methods Clin Dev.* 2017;4(192-203.

9. Anthony-Gonda K, Bardhi A, Ray A, Flerin N, Li M, Chen W, Ochsenbauer C, Kappes JC, Krueger W, Worden A, et al. Multispecific anti-HIV duoCAR-T cells display broad in vitro antiviral activity and potent in vivo elimination of HIV-infected cells in a humanized mouse model. *Sci Transl Med.* 2019;11(504):doi: 10.1126/scitranslmed.aav5685.

10. Zhen A, Peterson CW, Carrillo MA, Reddy SS, Youn CS, Lam BB, Chang NY, Martin HA, Rick JW, Kim J, et al. Long-term persistence and function of hematopoietic stem cell-derived chimeric antigen receptor T cells in a nonhuman primate model of HIV/AIDS. *PLoS Pathog.* 2017;13(12):e1006753.

11. Fox CH, Tenner-Racz K, Racz P, Firpo A, Pizzo PA, and Fauci AS. Lymphoid germinal centers are reservoirs of human immunodeficiency virus type 1 RNA. *J Infect Dis.* 1991;164(6):1051-7.

12. Miles B, and Connick E. TFH in HIV latency and as sources of replication-competent virus. *Trends Microbiol.* 2016;24(5):338-44.

13. Fukazawa Y, Lum R, Okoye AA, Park H, Matsuda K, Bae JY, Hagen SI, Shoemaker R, Deleage C, Lucero C, et al. B cell follicle sanctuary permits persistent productive simian immunodeficiency virus infection in elite controllers. *Nature Medicine.* 2015;21(2):132-9.

14. Aid M, Dupuy FP, Moysi E, Moir S, Haddad EK, Estes JD, Sekaly RP, Petrovas C, and Ribeiro SP. Follicular CD4 T helper cells as a major HIV reservoir compartment: A molecular perspective. *Front Immunol.* 2018;9(895.

15. Bronnimann MP, Skinner PJ, and Connick E. The B-cell follicle in HIV infection: Barrier to a cure. *Front Immunol.* 2018;9(20.

16. Barton K, Winckelmann A, and Palmer S. HIV-1 reservoirs during suppressive therapy. *Trends Microbiol.* 2016;24(5):345-55.

17. Smith MZ, Wightman F, and Lewin SR. HIV reservoirs and strategies for eradication. *Curr HIV/AIDS Rep.* 2012;9(1):5-15.

18. Hlavacek WS, Stilianakis NI, and Perelson AS. Influence of follicular dendritic cells on HIV dynamics. *Philos Trans R Soc Lond B Biol Sci.* 2000;355(1400):1051-8.

19. Smith BA, Gartner S, Liu Y, Perelson AS, Stilianakis NI, Keele BF, Kerkering TM, Ferreira-Gonzalez A, Szakal AK, Tew JG, et al. Persistence of infectious HIV on follicular dendritic cells. *J Immunol.* 2001;166(1):690-6.

20. Heesters BA, Lindqvist M, Vagefi PA, Scully EP, Schildberg FA, Altfeld M, Walker BD, Kaufmann DE, and Carroll MC. Follicular dendritic cells retain infectious HIV in cycling endosomes. *PLoS Pathog.* 2015;11(12):e1005285.

21. Zhang J, and Perelson AS. Contribution of follicular dendritic cells to persistent HIV viremia. *J Virol.* 2013;87(14):7893-901.

22. Hellmuth J, Valcour V, and Spudich S. CNS reservoirs for HIV: implications for eradication. *J Virus Erad.* 2015;1(2):67-71.

23. Thompson KA, Cherry CL, Bell JE, and McLean CA. Brain cell reservoirs of latent virus in presymptomatic HIV-infected individuals. *Am J Pathol.* 2011;179(4):1623-9.

24. Gama L, Abreu CM, Shirk EN, Price SL, Li M, Laird GM, Pate KA, Wietgrefe SW, O'Connor SL, Pianowski L, et al. Reactivation of simian immunodeficiency virus reservoirs in the brain of virally suppressed macaques. *Aids.* 2017;31(1):5-14.

25. Bednar MM, Sturdevant CB, Tompkins LA, Arrildt KT, Dukhovlinova E, Kincer LP, and Swanstrom R. Compartmentalization, viral evolution, and viral latency of HIV in the CNS. *Curr HIV/AIDS Rep.* 2015;12(2):262-71.

26. Petito CK, Chen H, Mastri AR, Torres-Munoz J, Roberts B, and Wood C. HIV infection of choroid plexus in AIDS and asymptomatic HIV-infected patients suggests that the choroid plexus may be a reservoir of productive infection. *J Neurovirol.* 1999;5(6):670-7.
27. Chaillon A, Gianella S, Wertheim JO, Richman DD, Mehta SR, and Smith DM. HIV migration between blood and cerebrospinal fluid or semen over time. *J Infect Dis.* 2014;209(10):1642-52.
28. Chun TW, Nickle DC, Justement JS, Meyers JH, Roby G, Hallahan CW, Kottilil S, Moir S, Mican JM, Mullins JI, et al. Persistence of HIV in gut-associated lymphoid tissue despite long-term antiretroviral therapy. *J Infect Dis.* 2008;197(5):714-20.
29. Lampinen TM, Critchlow CW, Kuypers JM, Hurt CS, Nelson PJ, Hawes SE, Coombs RW, Holmes KK, and Kiviat NB. Association of antiretroviral therapy with detection of HIV-1 RNA and DNA in the anorectal mucosa of homosexual men. *Aids.* 2000;14(5):F69-75.
30. Poles MA, Boscardin WJ, Elliott J, Taing P, Fuerst MM, McGowan I, Brown S, and Anton PA. Lack of decay of HIV-1 in gut-associated lymphoid tissue reservoirs in maximally suppressed individuals. *Journal of Acquired Immune Deficiency Syndromes.* 2006;43(1):65-8.
31. Di Stefano M, Favia A, Monno L, Lopalco P, Caputi O, Scardigno AC, Pastore G, Fiore JR, and Angarano G. Intracellular and cell-free (infectious) HIV-1 in rectal mucosa. *J Med Virol.* 2001;65(4):637-43.
32. Belmonte L, Olmos M, Fanin A, Parodi C, Bare P, Concetti H, Perez H, de Bracco MM, and Cahn P. The intestinal mucosa as a reservoir of HIV-1 infection after successful HAART. *Aids.* 2007;21(15):2106-8.
33. Brown D, and Mattapallil JJ. Gastrointestinal tract and the mucosal macrophage reservoir in HIV infection. *Clin Vaccine Immunol.* 2014;21(11):1469-73.

34. Beard BC, Trobridge GD, Ironside C, McCune JS, Adair JE, and Kiem HP. Efficient and stable MGMT-mediated selection of long-term repopulating stem cells in nonhuman primates. *Journal of Clinical Investigation.* 2010;120(7):2345-54.

35. Estes JD, Harris LD, Klatt NR, Tabb B, Pittaluga S, Paiardini M, Barclay GR, Smedley J, Pung R, Oliveira KM, et al. Damaged intestinal epithelial integrity linked to microbial translocation in pathogenic simian immunodeficiency virus infections. *PLoS Pathog.* 2010;6(8):e1001052.

36. Snyder JM, Washington IM, Birkland T, Chang MY, and Frevert CW. Correlation of versican expression, accumulation, and degradation during embryonic development by quantitative immunohistochemistry. *J Histochem Cytochem.* 2015;63(12):952-67.

37. Camacho SA, Kosco-Vilbois MH, and Berek C. The dynamic structure of the germinal center. *Immunol Today.* 1998;19(11):511-4.

38. Hong JJ, Chang KT, and Villinger F. The dynamics of T and B cells in lymph node during chronic HIV infection: TFH and HIV, unhappy dance partners? *Front Immunol.* 2016;7(522.

39. Subra C, and Trautmann L. Role of T lymphocytes in HIV neuropathogenesis. *Curr HIV/AIDS Rep.* 2019;16(3):236-43.

40. Veenhuis RT, Clements JE, and Gama L. HIV eradication strategies: Implications for the central nervous system. *Curr HIV/AIDS Rep.* 2019;16(1):96-104.

41. Peterson CW, Benne C, Polacino P, Kaur J, McAllister CE, Filali-Mouhim A, Obenza W, Pecor TA, Huang ML, Baldessari A, et al. Loss of immune homeostasis dictates SHIV rebound after stem-cell transplantation. *JCI Insight.* 2017;2(4):e91230.

42. Peterson CW, Wang J, Deleage C, Reddy S, Kaur J, Polacino P, Reik A, Huang ML, Jerome KR, Hu SL, et al. Differential impact of transplantation on peripheral and tissue-associated viral reservoirs: Implications for HIV gene therapy. *PLoS Pathog.* 2018;14(4):e1006956.

43. Peterson CW, Adair JE, Wohlfahrt ME, Deleage C, Radtke S, Rust B, Norman KK, Norgaard ZK, Schefter LE, Sghia-Hughes GM, et al. Autologous, gene-modified hematopoietic stem and progenitor cells repopulate the central nervous system with distinct clonal variants. *Stem Cell Reports.* 2019;13(1):91-104.

44. De Oliveira SN, Ryan C, Giannoni F, Hardee CL, Tremcinska I, Katebian B, Wherley J, Sahaghian A, Tu A, Grogan T, et al. Modification of hematopoietic stem/progenitor cells with CD19-specific chimeric antigen receptors as a novel approach for cancer immunotherapy. *Hum Gene Ther.* 2013;24(10):824-39.

45. Tran AC, Zhang D, Byrn R, and Roberts MR. Chimeric zeta-receptors direct human natural killer (NK) effector function to permit killing of NK-resistant tumor cells and HIV-infected T lymphocytes. *J Immunol.* 1995;155(2):1000-9.

46. Schirrmann T, and Pecher G. Specific targeting of CD33(+) leukemia cells by a natural killer cell line modified with a chimeric receptor. *Leuk Res.* 2005;29(3):301-6.

47. Li L, Liu LN, Feller S, Allen C, Shivakumar R, Fratantoni J, Wolfraim LA, Fujisaki H, Campana D, Chopas N, et al. Expression of chimeric antigen receptors in natural killer cells with a regulatory-compliant non-viral method. *Cancer Gene Ther.* 2010;17(3):147-54.

48. Hege KM, Cooke KS, Finer MH, Zsebo KM, and Roberts MR. Systemic T cell-independent tumor immunity after transplantation of universal receptor-modified bone marrow into SCID mice. *J Exp Med.* 1996;184(6):2261-9.

49. Han J, Chu J, Keung Chan W, Zhang J, Wang Y, Cohen JB, Victor A, Meisen WH, Kim SH, Grandi P, et al. CAR-engineered NK cells targeting wild-type EGFR and EGFRvIII enhance killing of glioblastoma and patient-derived glioblastoma stem cells. *Sci Rep.* 2015;5(11483.

50. Roberts MR, Cooke KS, Tran AC, Smith KA, Lin WY, Wang M, Dull TJ, Farson D, Zsebo KM, and Finer MH. Antigen-specific cytolysis by neutrophils and NK cells expressing chimeric immune receptors bearing zeta or gamma signaling domains. *J Immunol.* 1998;161(1):375-84.

51. Mehta RS, and Rezvani K. Chimeric antigen receptor expressing natural killer dells for the immunotherapy of cancer. *Front Immunol.* 2018;9(283.

52. Hagn M, and Jahrsdorfer B. Why do human B cells secrete granzyme B? Insights into a novel B-cell differentiation pathway. *Oncoimmunology.* 2012;1(8):1368-75.

53. Hagn M, Schwesinger E, Ebel V, Sontheimer K, Maier J, Beyer T, Syrovets T, Laumonnier Y, Fabricius D, Simmet T, et al. Human B cells secrete granzyme B when recognizing viral antigens in the context of the acute phase cytokine IL-21. *J Immunol.* 2009;183(3):1838-45.

54. Lindner S, Dahlke K, Sontheimer K, Hagn M, Kaltenmeier C, Barth TF, Beyer T, Reister F, Fabricius D, Lotfi R, et al. Interleukin 21-induced granzyme B-expressing B cells infiltrate tumors and regulate T cells. *Cancer Res.* 2013;73(8):2468-79.

55. Hagn M, Panikkar A, Smith C, Balfour HH, Jr., Khanna R, Voskoboinik I, and Trapani JA. B cell-derived circulating granzyme B is a feature of acute infectious mononucleosis. *Clin Transl Immunology.* 2015;4(6):e38.

56. Hagn M, Sontheimer K, Dahlke K, Brueggemann S, Kaltenmeier C, Beyer T, Hofmann S, Lunov O, Barth TF, Fabricius D, et al. Human B cells differentiate into granzyme B-secreting cytotoxic B lymphocytes upon incomplete T-cell help. *Immunol Cell Biol.* 2012;90(4):457-67.

57. Frances R, Tumang JR, and Rothstein TL. B-1 cells are deficient in Lck: defective B cell receptor signal transduction in B-1 cells occurs in the absence of elevated Lck expression. *J Immunol.* 2005;175(1):27-31.

58. Scielzo C, Camporeale A, Geuna M, Alessio M, Poggi A, Zocchi MR, Chilosi M, Caligaris-Cappio F, and Ghia P. ZAP-70 is expressed by normal and malignant human B-cell subsets of different maturational stage. *Leukemia.* 2006;20(4):689-95.

59. Kuhlmann AS, Haworth KG, Barber-Axthelm IM, Ironside C, Giese MA, Peterson CW, and Kiem HP. Long-term persistence of anti-HIV broadly neutralizing antibody-secreting hematopoietic cells in humanized mice. *Mol Ther.* 2019;27(1):164-77.

60. Telwatte S, Kim P, Chen TH, Milush JM, Somsouk M, Deeks SG, Hunt PW, Wong JK, and Yukl SA. Mechanistic differences underlying HIV latency in the gut and blood contribute to differential responses to latency-reversing agents. *AIDS.* 2020:[Online ahead of print; doi: 10.1097/qad.0000000000002684].

61. Massengale M, Wagers AJ, Vogel H, and Weissman IL. Hematopoietic cells maintain hematopoietic fates upon entering the brain. *J Exp Med.* 2005;201(10):1579-89.

62. Muller A, Brandenburg S, Turkowski K, Muller S, and Vajkoczy P. Resident microglia, and not peripheral macrophages, are the main source of brain tumor mononuclear cells. *Int J Cancer.* 2015;137(2):278-88.

63. Karperien A, Ahammer H, and Jelinek HF. Quantitating the subtleties of microglial morphology with fractal analysis. *Front Cell Neurosci.* 2013;7(3.

64. Biffi A, Montini E, Lorioli L, Cesani M, Fumagalli F, Plati T, Baldoli C, Martino S, Calabria A, Canale S, et al. Lentiviral hematopoietic stem cell gene therapy benefits metachromatic leukodystrophy. *Science.* 2013;341(6148):1233158.

65. Wallet C, De Rovere M, Van Assche J, Daouad F, De Wit S, Gautier V, Mallon PWG, Marcello A, Van Lint C, Rohr O, et al. Microglial cells: The main HIV-1 reservoir in the brain. *Front Cell Infect Microbiol.* 2019;9(362.

66. Parrish HL, Glassman CR, Keenen MM, Deshpande NR, Bronnimann MP, and Kuhns MS. A transmembrane domain GGxxG motif in CD4 contributes to its Lck-independent function but does not mediate CD4 dimerization. *PLoS One.* 2015;10(7):e0132333.

67. Kroese FG, Wubbena AS, Seijen HG, and Nieuwenhuis P. Germinal centers develop oligoclonally. *Eur J Immunol.* 1987;17(7):1069-72.

68. Eglitis MA, and Mezey E. Hematopoietic cells differentiate into both microglia and macroglia in the brains of adult mice. *Proc Natl Acad Sci U S A.* 1997;94(8):4080-5.

69. Prinz M, Erny D, and Hagemeyer N. Ontogeny and homeostasis of CNS myeloid cells. *Nat Immunol.* 2017;18(4):385-92.

70. Kierdorf K, Katzmarski N, Haas CA, and Prinz M. Bone marrow cell recruitment to the brain in the absence of irradiation or parabiosis bias. *PLoS One.* 2013;8(3):e58544.

71. Priller J, Flugel A, Wehner T, Boentert M, Haas CA, Prinz M, Fernandez-Klett F, Prass K, Bechmann I, de Boer BA, et al. Targeting gene-modified hematopoietic cells to the central nervous system: use of green fluorescent protein uncovers microglial engraftment. *Nature Medicine.* 2001;7(12):1356-61.

72. Filip S, Mokry J, Vavrova J, Sinkorova Z, Micuda S, Sponer P, Filipova A, Hrebikova H, and Dayanithi G. The peripheral chimerism of bone marrow-derived stem cells after transplantation: regeneration of gastrointestinal tissues in lethally irradiated mice. *J Cell Mol Med.* 2014;18(5):832-43.

73. Hirayama AV, Gauthier J, Hay KA, Voutsinas JM, Wu Q, Gooley T, Li D, Cherian S, Chen X, Pender BS, et al. The response to lymphodepletion impacts PFS in patients with aggressive non-Hodgkin lymphoma treated with CD19 CAR T cells. *Blood.* 2019;133(17):1876-87.

74. Kochenderfer JN, Somerville RPT, Lu T, Shi V, Bot A, Rossi J, Xue A, Goff SL, Yang JC, Sherry RM, et al. Lymphoma remissions caused by anti-CD19 chimeric antigen receptor T cells are associated with high serum interleukin-15 levels. *J Clin Oncol.* 2017;35(16):1803-13.

75. Shaw PJ, Nath CE, and Lazarus HM. Not too little, not too much-just right! (Better ways to give high dose melphalan). *Bone Marrow Transplant.* 2014;49(12):1457-65.

76. Mamcarz E, Zhou S, Lockey T, Abdelsamed H, Cross SJ, Kang G, Ma Z, Condori J, Dowdy J, Triplett B, et al. Lentiviral gene therapy combined with low-dose busulfan in infants with SCID-X1. *N Engl J Med.* 2019;380(16):1525-34.

77. Chandrasekaran D, Nakamoto B, Watts KL, Kiem HP, and Papayannopoulou T. Modeling promising nonmyeloablative conditioning regimens in nonhuman primates. *Hum Gene Ther.* 2014;25(12):1013-22.

78. Czechowicz A, Palchaudhuri R, Scheck A, Hu Y, Hoggatt J, Saez B, Pang WW, Mansour MK, Tate TA, Chan YY, et al. Selective hematopoietic stem cell ablation using CD117-antibody-drug-conjugates enables safe and effective transplantation with immunity preservation. *Nat Commun.* 2019;10(1):617.

79. Arai Y, Choi U, Corsino CI, Koontz SM, Tajima M, Sweeney CL, Black MA, Feldman SA, Dinauer MC, and Malech HL. Myeloid conditioning with c-kit targeted CAR-T cells enables donor stem cell engraftment. *Mol Ther.* 2018;26(5):1181-97.

80. Gao C, Schroeder JA, Xue F, Jing W, Cai Y, Scheck A, Subramaniam S, Rao S, Weiler H, Czechowicz A, et al. Nongenotoxic antibody-drug conjugate conditioning enables safe and effective platelet gene therapy of hemophilia A mice. *Blood Adv.* 2019;3(18):2700-11.

81. Palchaudhuri R, Saez B, Hoggatt J, Schajnovitz A, Sykes DB, Tate TA, Czechowicz A, Kfoury Y, Ruchika F, Rossi DJ, et al. Non-genotoxic conditioning for hematopoietic stem cell transplantation using a hematopoietic-cell-specific internalizing immunotoxin. *Nat Biotechnol.* 2016;34(7):738-45.

82. Reeves DB, Peterson CW, Kiem HP, and Schiffer JT. Autologous stem cell transplantation disrupts adaptive immune responses during rebound simian/human immunodeficiency virus viremia. *J Virol.* 2017;91(13).

83. Howe AY, Jung JU, and Desrosiers RC. Zeta chain of the T-cell receptor interacts with nef of simian immunodeficiency virus and human immunodeficiency virus type 2. *J Virol.* 1998;72(12):9827-34.

84. Iafrate AJ, Bronson S, and Skowronski J. Separable functions of Nef disrupt two aspects of T cell receptor machinery: CD4 expression and CD3 signaling. *EMBO J.* 1997;16(4):673-84.

85. Munch J, Janardhan A, Stolte N, Stahl-Hennig C, Ten Haaft P, Heeney JL, Swigut T, Kirchhoff F, and Skowronski J. T-cell receptor:CD3 down-regulation is a selected in vivo function of simian immunodeficiency virus Nef but is not sufficient for effective viral replication in rhesus macaques. *J Virol.* 2002;76(23):12360-4.

86. Anolik J, Looney RJ, Bottaro A, Sanz I, and Young F. Down-regulation of CD20 on B cells upon CD40 activation. *Eur J Immunol.* 2003;33(9):2398-409.

87. Victora GD. SnapShot: the germinal center reaction. *Cell.* 2014;159(3):700- e1.

88. Vinuesa CG, Linterman MA, Goodnow CC, and Randall KL. T cells and follicular dendritic cells in germinal center B-cell formation and selection. *Immunol Rev.* 2010;237(1):72-89.

89. Lin WY, and Roberts MR. Developmental dissociation of T cells from B, NK, and myeloid cells revealed by MHC class II-specific chimeric immune receptors bearing TCR-zeta or FcR-gamma chain signaling domains. *Blood.* 2002;100(8):3045-8.

90. Egelhofer M, Brandenburg G, Martinius H, Schult-Dietrich P, Melikyan G, Kunert R, Baum C, Choi I, Alexandrov A, and von Laer D. Inhibition of human immunodeficiency virus type 1 entry in cells expressing gp41-derived peptides. *Journal of Virology.* 2004;78(2):568-75.

91. Brauer F, Schmidt K, Zahn RC, Richter C, Radeke HH, Schmitz JE, von Laer D, and Egerer L. A rationally engineered anti-HIV peptide fusion inhibitor with greatly reduced immunogenicity. *Antimicrobial Agents & Chemotherapy.* 2013;57(2):679-88.

92. Song RJ, Chenine AL, Rasmussen RA, Ruprecht CR, Mirshahidi S, Grisson RD, Xu W, Whitney JB, Goins LM, Ong H, et al. Molecularly cloned SHIV-1157ipd3N4: a highly replication- competent, mucosally transmissible R5 simian-human immunodeficiency virus encoding HIV clade C Env. *Journal of Virology.* 2006;80(17):8729-38.

93. Peterson CW, Haworth KG, Burke BP, Polacino P, Norman KK, Adair JE, Hu SL, Bartlett JS, Symonds GP, and Kiem HP. Multilineage polyclonal engraftment of Cal-1 gene-modified cells and in vivo selection after SHIV infection in a nonhuman primate model of AIDS. *Mol Ther Methods Clin Dev.* 2016;3(16007.

94. Ormerod LD, Osborn KG, Lowenstine LJ, Meyer PR, Parker JW, Smith RE, and Taylor CR. Lymphoid immunohistochemistry of macaque primates. *Clin Exp Immunol.* 1988;74(3):435-42.

95. Kap YS, van Meurs M, van Driel N, Koopman G, Melief MJ, Brok HP, Laman JD, and t Hart BA. A monoclonal antibody selection for immunohistochemical examination of lymphoid tissues from non-human primates. *J Histochem Cytochem.* 2009;57(12):1159-67.

96. Stack EC, Wang C, Roman KA, and Hoyt CC. Multiplexed immunohistochemistry, imaging, and quantitation: a review, with an assessment of Tyramide signal amplification, multispectral imaging and multiplex analysis. *Methods.* 2014;70(1):46-58.

97. Bankhead P, Loughrey MB, Fernandez JA, Dombrowski Y, McArt DG, Dunne PD, McQuaid S, Gray RT, Murray LJ, Coleman HG, et al. QuPath: Open source software for digital pathology image analysis. *Sci Rep.* 2017;7(1):16878.

98. Rueden CT, Schindelin J, Hiner MC, DeZonia BE, Walter AE, Arena ET, and Eliceiri KW. ImageJ2: ImageJ for the next generation of scientific image data. *BMC Bioinformatics.* 2017;18(1):529.

99. Schneider CA, Rasband WS, and Eliceiri KW. NIH Image to ImageJ: 25 years of image analysis. *Nat Methods.* 2012;9(7):671-5.

100. Schindelin J, Arganda-Carreras I, Frise E, Kaynig V, Longair M, Pietzsch T, Preibisch S, Rueden C, Saalfeld S, Schmid B, et al. Fiji: an open-source platform for biological-image analysis. *Nat Methods.* 2012;9(7):676-82.
101. Mutterer J, and Zinck E. Quick-and-clean article figures with FigureJ. *J Microsc.* 2013;252(1):89-91.
102. Otsu N. A threshold selection method from gray-level histograms. *IEEE Trans Syst Man Cybern.* 1979;9(1):62-6.
103. Camp RL, Chung GG, and Rimm DL. Automated subcellular localization and quantification of protein expression in tissue microarrays. *Nat Med.* 2002;8(11):1323-7.
104. Suryawanshi GW, Khamaikawin W, Wen J, Shimizu S, Arokium H, Xie Y, Wang E, Kim S, Choi H, Zhang C, et al. The clonal repopulation of HSPC gene modified with anti-HIV-1 RNAi is not affected by preexisting HIV-1 infection. *Sci Adv.* 2020;6(30):eaay9206.
105. Suryawanshi GW, Xu S, Xie Y, Chou T, Kim N, Chen ISY, and Kim S. Bidirectional retroviral integration site PCR methodology and quantitative data analysis workflow. *J Vis Exp.* 2017124).

FIGURES WITH LEGENDS

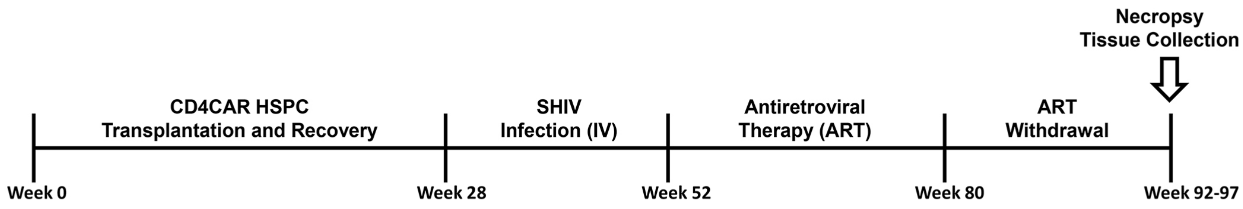

Figure 1. Study schematic for CAR 1, CAR 2, Control 1, and Control 2 animals. A total of 4 pigtail macaques were transplanted with autologous HSPCs modified to express CD4CAR (n = 2) or a control CD4CARΔζ (n = 2) which lacks intracellular signaling function, but retains the extracellular domain for immunolabeling. Following 28 weeks of post-transplant recovery, animals were infected with SHIV-1157ipd3N4 via the intravenous (IV) route. Approximately 6 months later, antiretroviral therapy (ART) was initiated, then withdrawn 28 weeks later, in order to compare the persistence of CD4CAR and control-modified cells in low and high antigen conditions, respectively. Following ART withdrawal, animals were monitored for approximately 15 weeks prior to necropsy.

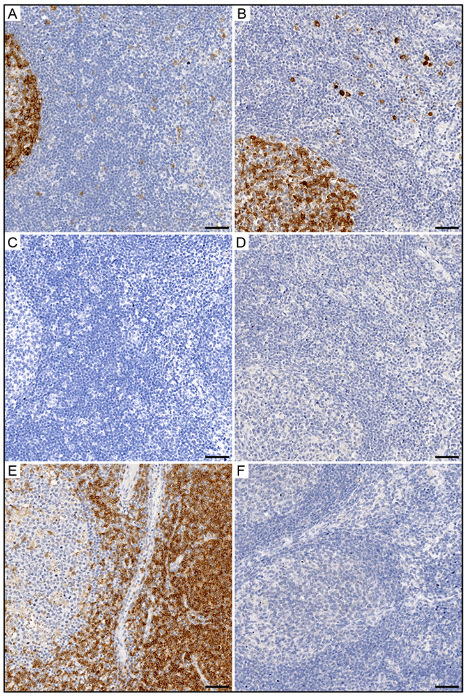

Figure 2. Anti-CD4 antibody clone SP35 specifically marks CAR$^+$ cells. Specific CD4 (SP35) immunoreactivity in germinal centers from mesenteric lymph node sections from macaques that received either CD4CAR **(A)** or CD4CARΔζ **(B)**; sparse marking in the parafollicular zone was also observed. No immunoreactivity was seen in paired adjacent CD4CAR **(C)** or CD4CARΔζ **(D)** tissue sections labeled with an isotype control. Positive control: Labeling of human tonsil shows specific immunoreactivity, which is predominately in the parafollicular zone and consistent with CD4$^+$ T-cell marking **(E)**. Negative control: no immunoreactivity is seen in a control mesenteric lymph node section from a macaque that did not receive either CD4CAR or CD4CARΔζ **(F)**, indicating that the CD4 (SP35) antibody clone does not cross-react with the endogenous pigtail macaque CD4 antigen. Brown: immunoreactivity for human CD4CAR; blue: hematoxylin counterstain. The experiment was repeated twice to confirm the specificity of the CD4 (SP35) antibody for the human-derived CDCAR or CD4CARΔζ. Scale bar: 50μm.

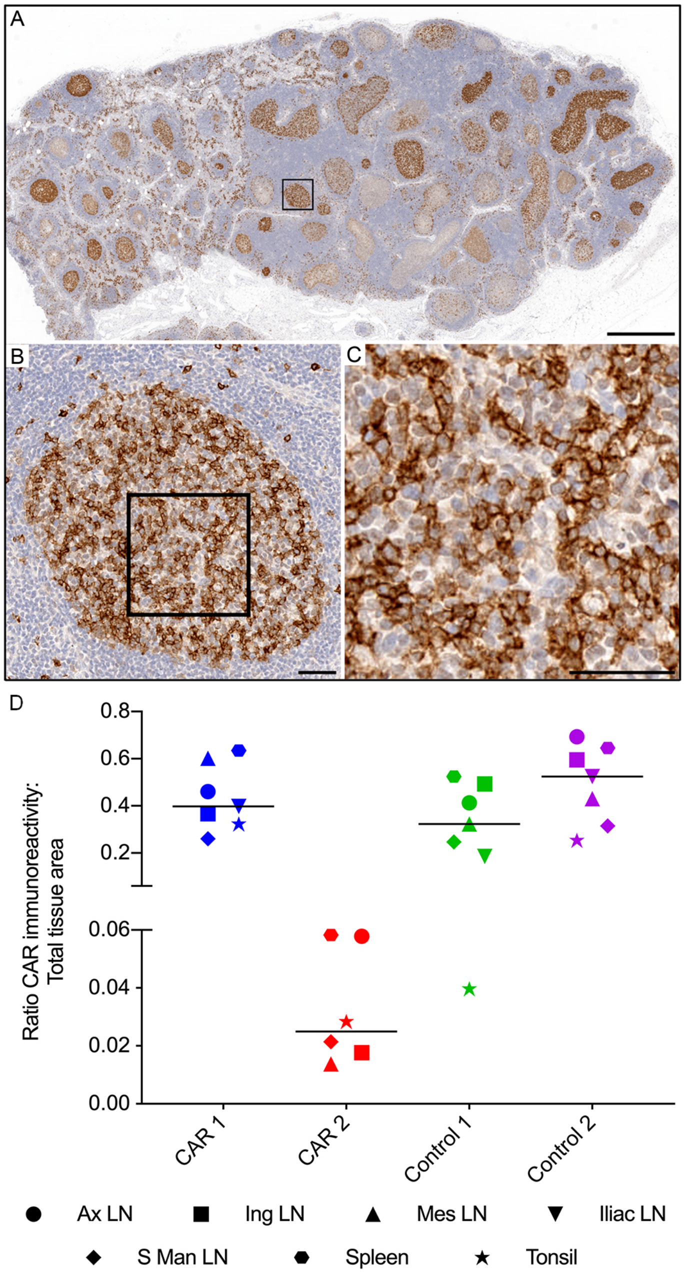

Figure 3. HSPC-derived CD4CAR expressing cells localize to central and peripheral lymphoid tissue germinal centers. Low **(A)**, medium **(B)**, and high **(C)** magnification photomicrographs of an iliac LN from CAR 1, illustrating CD4CAR$^+$ cells localizing in the germinal centers. Brown: immunoreactivity for human CD4CAR; blue: hematoxylin counterstain. **D)** Quantification of CD4CAR-labeled lymphoid GC's as a percentage of total lymphoid GC tissue area in both CD4CAR and CD4CARΔζ macaques (n=4 macaques; 6-7 lymphoid tissues per macaque). Thresholds for average % CD4CAR GC marking were set using representative lymphoid tissues from macaques that did not receive a CAR (0.0031%). The chart shows individual data points with the median. Scale bars: (A) 1mm; (B, C) 50μm.

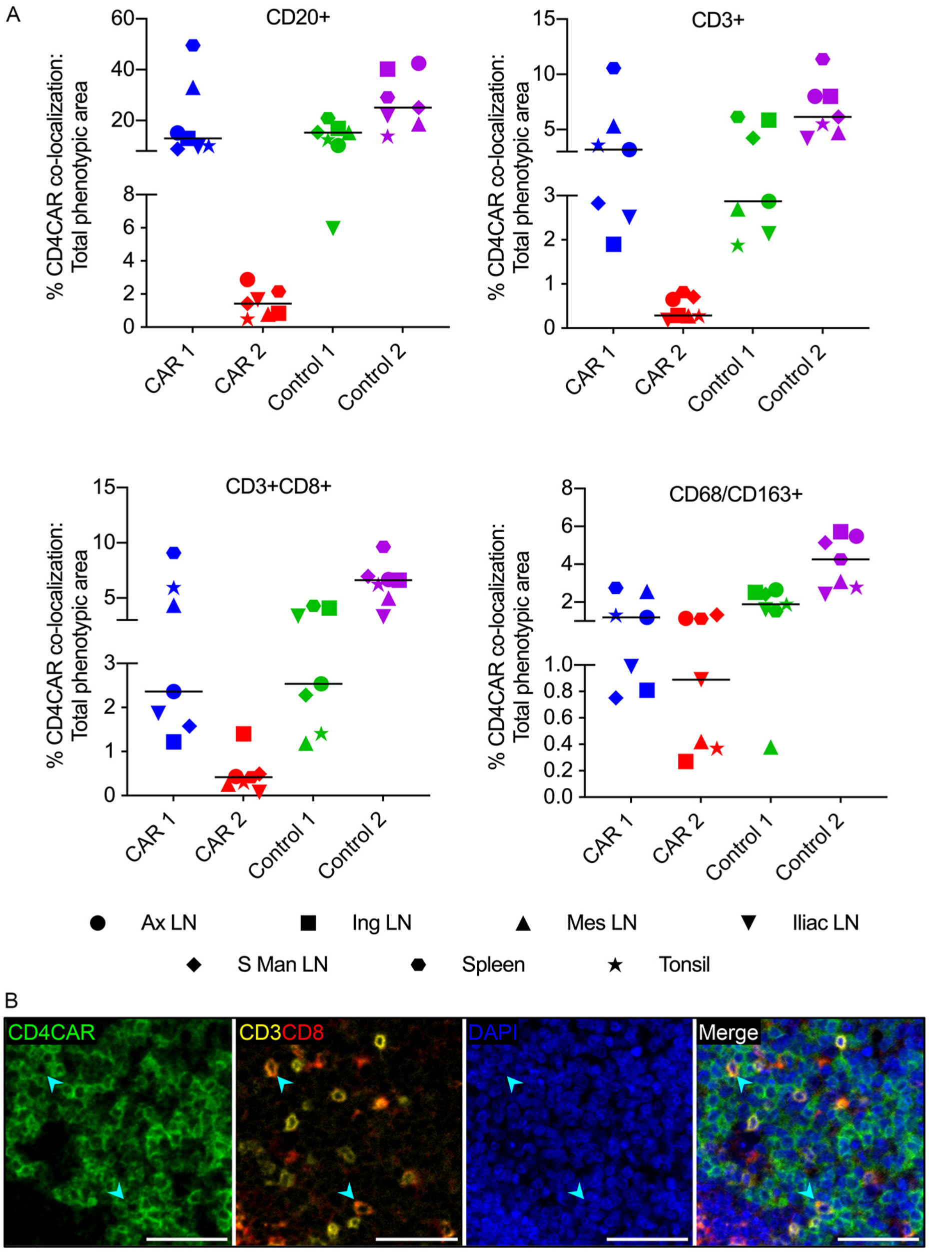

Figure 4. Multilineage engraftment of HSPC-derived CAR⁺ cells in lymphoid germinal centers. **A)** Percentages of total B-cell ($CD20^+$), T-cell ($CD3^+$), CTL ($CD3^+CD8^+$), and monocyte/macrophage ($CD68/CD163^+$) immunophenotypic area that colocalized with CD4CAR immunoreactivity in GCs (n=4 macaques; 6-7 lymphoid tissues per macaque). The charts show individual data points with medians. **B)** Representative fluorescent mIHC photomicrographs of CD4CAR (green), CD3 (yellow), CD8 (red), and DAPI nuclear counterstain (blue); from CAR 1 mesenteric LN. Arrowheads highlight colocalization between CD4CAR and CD3/CD8 markers, indicating the presence of $CD4CAR^+$ CTLs within the germinal center. Scale bar: 50μm.

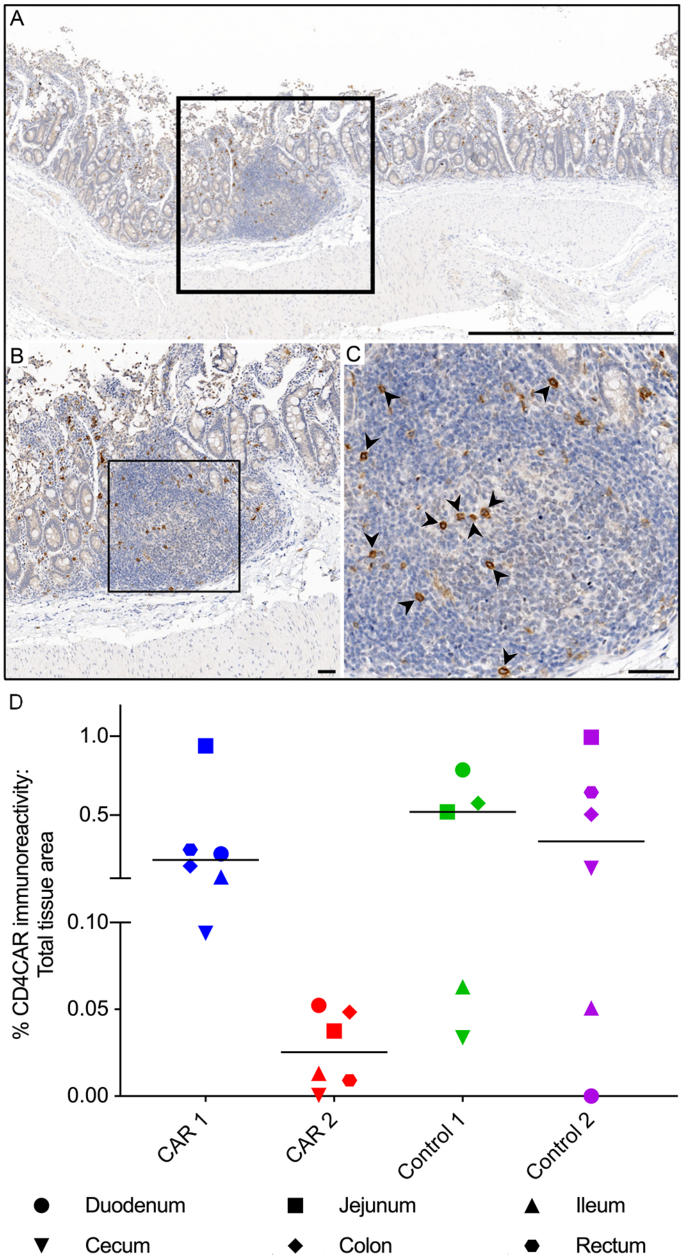

Figure 5. HSPC-derived CD4CAR⁺ cells localize to the GIT. Low **(A)**, medium **(B)**, and high **(C)** magnification photomicrographs of a jejunum section from CAR 2, illustrating localization of CD4CAR⁺ cells (arrowheads) within gastrointestinal lymphoid tissue. Brown: immunoreactivity for CD4CAR; blue: hematoxylin counterstain. **D)** The amount of CD4CAR-labeled gastrointestinal lymphoid tissue and adjacent submucosal tissue, as a percentage of the total tissue area in both CAR and Control macaques (n=4 macaques; 5-6 GIT per macaque). Threshold levels were set to average % CD4CAR GIT marking in representative tissues from macaques that did not receive a CAR (0.007%). Chart shows individual data points with the median. Scale bar: (*A*) 1mm; (*B, C*) 50μm.

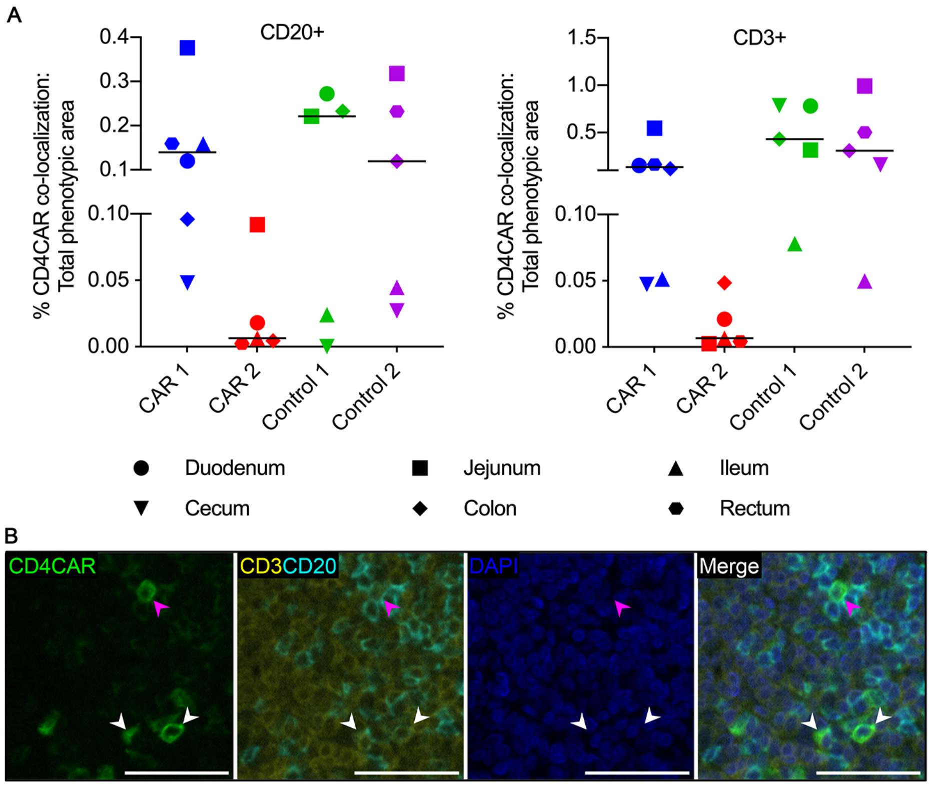

Figure 6. Enriched engraftment of HSPC-derived CAR^{+} T-cells in GIT. A) Percentages of total B-cell (CD20^{+}) and T-cell (CD3^{+}) immunoreactivity that colocalized with CD4CAR (n=4 macaques; 5-6 GIT tissues per macaque). Charts show individual data points with medians. **B)** Representative fluorescent mIHC photomicrographs of CD4CAR (green), CD3 (yellow), CD20 (cyan), and DAPI nuclear counterstain (blue); from Control 1 ileum. White arrowheads: CD4CAR^{+} T-cells. Magenta arrowhead: CD4CAR^{+} B-cell. Scale bar: 50µm.

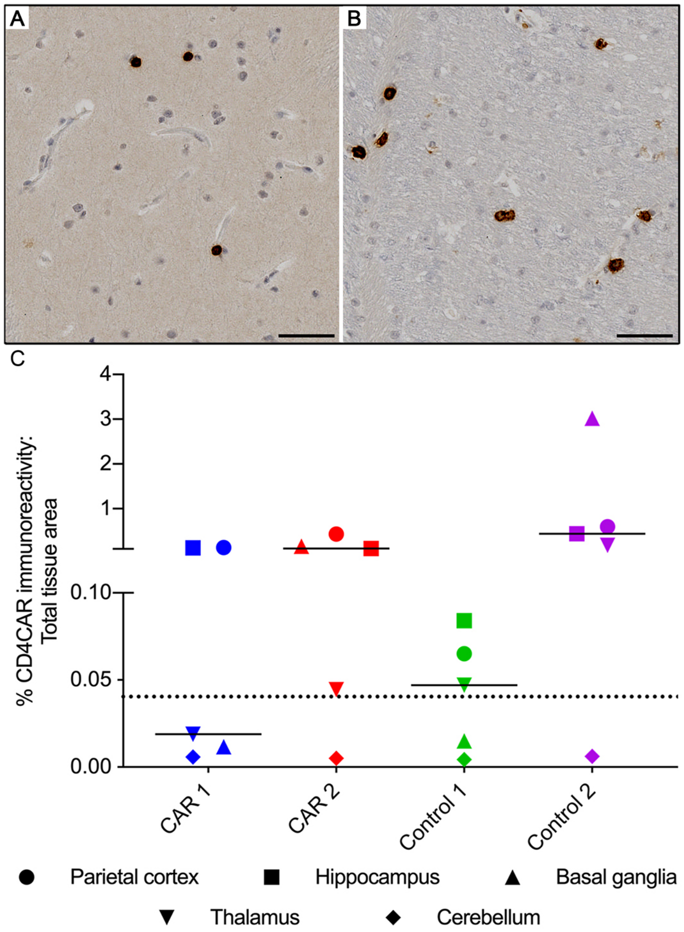

Figure 7. HSPC-derived CD4CAR^{+} cells engraft and persist in the CNS. Representative photomicrographs of a basal ganglia section from Control 2, illustrating CD4CAR^{+} cells localizing to gray **(A)** and white **(B)** matter in the CNS. Brown: immunoreactivity for human CD4CAR; blue: hematoxylin counterstain. **C)** Quantification of CD4CAR-labeled CNS tissue (gray and white matter) as a percentage of the total CNS tissue area in both CAR and Control macaques (n=4 macaques; 5 CNS tissues per macaque). Dotted line: threshold signal set based on average percent CD4CAR immunoreactivity in representative CNS sections from CAR^{-} animals (0.04%). Chart shows individual data points with the median. Scale bar: 50µm.

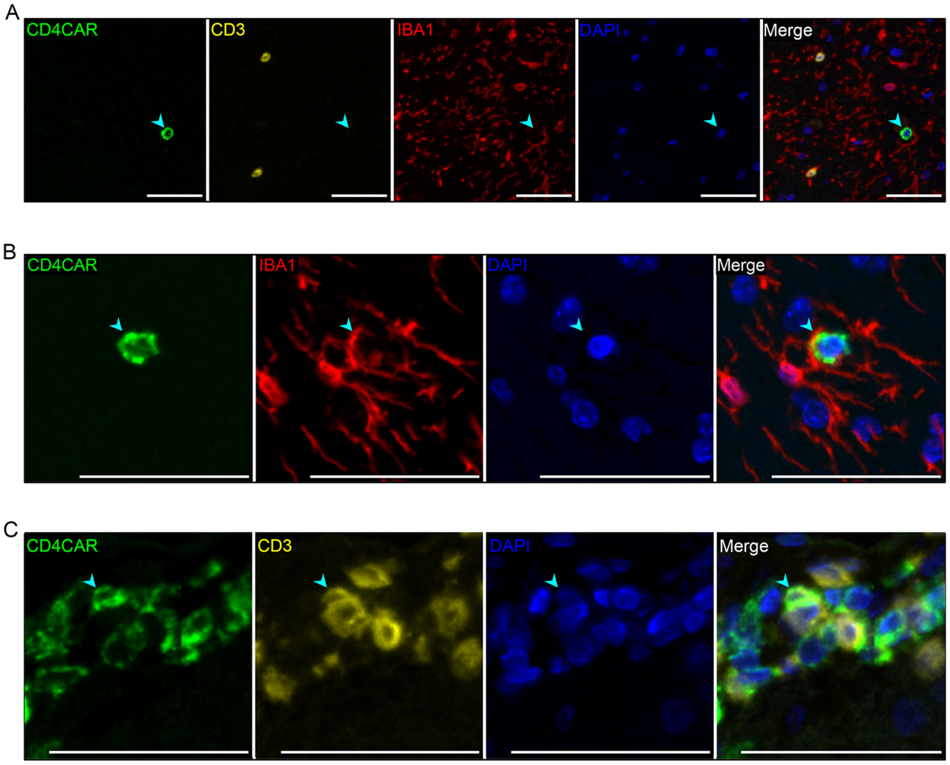

Figure 8. **Characterization of HSPC-derived CAR^{+} cells in the CNS.** Representative fluorescent mIHC photomicrographs from Control 2, illustrating the presence of HSPC-derived CD4CAR^{+} cells (arrowheads) (n=4 macaques; 5 CNS tissues per macaque). **A)** Basal ganglia: Lack of colocalization between CD4CAR (green) and CD3 (yellow) or IBA-1 (red). **B)** Thalamus: Colocalization of CD4CAR with IBA-1, indicating a CD4CAR^{+} myeloid cell. **C)** Hippocampus: Colocalization of CD4CAR with CD3, indicating a CD4CAR^{+} T-cell. Scale bar: 50μm.

SUPPLEMENTAL DATA FOR

Stem cell-derived CAR-T cells traffic to HIV reservoirs in macaques

Isaac Barber-Axthelm[1,2], Valerie Barber-Axthelm[1], Kai Yin Sze[1], Anjie Zhen[3,4], Gajendra W Suryawanshi[4,5], Irvin SY Chen[3,4,5], Jerome A. Zack[3,4,5], Scott G. Kitchen[3,4], Hans-Peter Kiem[1,6,7*], Christopher Peterson[1,6*]

List of Supplemental Items

1. **Supplemental Table 1.** Infused HSPC Product Gene Marking and Engraftment.
2. **Supplemental Table 2.** SHIV Plasma Viral Load and CD4CAR Gene Marking at Necropsy.
3. **Supplemental Table 3.** Overview of fluorescent mIHC staining approach by tissue type.
4. **Supplemental Table 4.** Summary of IHC-based tissue analysis.
5. **Supplemental Figure 1.** Polyclonal repopulation of CD4CAR-modified, HSPC-derived PBMC in vivo.
6. **Supplemental Figure 2.** PCR-based quantification of CD4CAR gene marking in necropsy tissues from CAR1/CAR2 and Control 1/Control 2.
7. **Supplemental Figure 3.** PCR-based quantification of CD4CAR gene marking in necropsy tissues from CAR1/CAR2 and Control 1/Control 2.
8. **Supplemental Figure 4.** Statistical comparison of total CD4CAR immunoreactive tissue area for CAR 1/CAR2 vs. Control 1/Control 2.
9. **Supplemental Figure 5.** Statistical comparison of subset-specific CD4CAR immunoreactive lymphoid GC area for CAR 1/CAR2 vs. Control 1/Control 2.
10. **Supplemental Figure 6.** Enriched engraftment of HSPC-derived CAR$^+$ B-cells in lymphoid germinal centers.

11. **Supplemental Figure 7.** HSPC-derived CAR^{+} cells in lymphoid GC express the proliferation marker Ki-67.
12. **Supplemental Figure 8.** Statistical comparison of subset-specific CD4CAR immunoreactive GIT area for CAR 1/CAR2 vs. Control 1/Control 2.
13. **Supplemental Figure 9.** RNAscope and DNAscope detection of SHIV nucleic acids *in situ.*
14. **Supplemental Figure 10.** Conceptual schematic for the binarized pixel colocalization algorithm performed by the Multiplex Pixel Colocalization plugin.

Supplemental Table 1. Infused HSPC product gene marking and engraftment

ID	HSPC Infusion Product Gene Marking (Colony PCR)	Days to Neutrophil Recovery (CBC)*	Days to Platelet Recovery (CBC)*	%CD4CAR^{+} PBMC Pre-SHIV Infection	
				Flow Cytometry[†]	PCR[‡]
CAR 1	4.7%	13	61	0.10	3.26
CAR 2	26.7%	11	40	0.02	8.79
Control 1	40.0%	9	26	0.20	3.11
Control 2	14.8%	12	22	0.80	1.55

*Healthy minimum values as defined in Zhen, Peterson et al PLoS Pathogens 2017: 1,800 neutrophils/uL whole blood, 262,000 platelets/uL whole blood
†Measured 4-5 weeks prior to SHIV infection
‡Measured 4-10 weeks prior to SHIV infection

Supplemental Table 2. SHIV plasma viral load and CD4CAR gene marking at necropsy.

ID	Plasma Viral Load (copies/mL)	%CD4CAR$^+$ PBMC	
		Flow Cytometry	PCR
CAR 1	492,133	2.71	6.79
CAR 2	999	4.30	15.63
Control 1	187,708	0.68	3.84
Control 2	884,846	0.64	2.72

Supplemental Table 3. Overview of fluorescent mIHC staining approach by tissue type.

Position	Primary Antibody				Secondary	
	Target	Clone (Host)	Manufacturer (Product number)	Dilution	Antibody	OPAL Fluorophore
**Lymphoid germinal centers (phenotype)**						
1	CD20	Polyclonal (Rabbit)	Thermo Fisher (PA5-16701)	1:2,000	Opal Ms/Rbt Secondary	540
2	CD3	Polyclonal (Rabbit)	Dako (A0452)	1:800	Opal Ms/Rbt Secondary	570
3	CD68	Polyclonal (Rabbit)	Thermo Fisher (PA5-32330)	1:200	Opal Ms/Rbt Secondary	690†
	CD163	EP324 (Rabbit)	BioSB (BSB3276)	1:2,000	Opal Ms/Rbt Secondary	
4	CD4	SP35 (Rabbit)	Cell Marque (104R-16)	1:100	Opal Ms/Rbt Secondary	520
5	CD8	EP334 (Rabbit)	BioSB (BSB2849)	1:2,000	Opal Ms/Rbt Secondary	650
6	CD35	Polyclonal (Rabbit)	Sigma (HPA049348)	1:1,000	Opal Ms/Rbt Secondary	620
**Central nervous system**						
1	IBA-1	Polyclonal (Goat)	Abcam (AB5076)	1:1,000	Vector Immpress Goat Polymer	520
2	CD20	L26 (Mouse)	Dako (M0755)	1:10,000	Opal Ms/Rbt Secondary	540
3	CD4	SP35 (Rabbit)	Cell Marque (104R-16)	1:200	Opal Ms/Rbt Secondary	620
4	CD3	SP7 (Rabbit)	Thermo Fisher (RM-9107)	1:200	Opal Ms/Rbt Secondary	690
**Gastrointestinal tract (GIT)**						
1	CD20	L26 (Mouse)	Dako (M0755)	1:10,000	Opal Ms/Rbt Secondary	540
2	CD4	SP35 (Rabbit)	Cell Marque (104R-16)	1:200	Opal Ms/Rbt Secondary	620
3	CD3	SP7 (Rabbit)	Thermo Fisher (RM-9107)	1:200	Opal Ms/Rbt Secondary	690

Position	Primary Antibody				Secondary	
	**Target**	**Clone (Host)**	**Manufacturer (Product number)**	**Dilution**	**Antibody**	**OPAL Fluorophore**
**Lymphoid germinal centers (proliferation)**						
1	CD20	L26 (Mouse)	Dako (M0755)	1:10,000	Opal Ms/Rbt Secondary	520
2	Ki-67	D3B5 (Rabbit)	Cell Signaling (12202S)	1:500	Opal Ms/Rbt Secondary	620
3	CD4	SP35 (Rabbit)	Cell Marque (104R-16)	1:200	Opal Ms/Rbt Secondary	540
4	CD3	SP7 (Rabbit)	Thermo (RM-9107)	1:200	Opal Ms/Rbt Secondary	690

Supplemental Table 4. Summary of IHC-based tissue analysis.

	CAR 1	CAR 2	Control 1	Control 2
% CD4CAR marking in PBMC at tissue time point	6.79	9.67	5.13	1.13
Lymphoid tissue sites evaluated	7	7	7	7
Germinal centers analyzed per tissue site: Average (range)	57 (24–78)	56 (19–162)	41 (15–84)	107 (54–146)
Germinal center area evaluated (μm^2): average (range)	4.66×10^{6} (2.45×10^{6}–1.06×10^{7})	2.54×10^{6} (6.71×10^{5}–6.33×10^{6})	1.99×10^{6} (7.94×10^{5}–3.64×10^{6})	8.12×10^{6} (5.72×10^{6}–1.04×10^{7})
Average germinal center size (μm^2/GC)	80461	50839	57673	81079
CNS tissue sites evaluated	5	5	5	5
CNS Area evaluated (μm^2): Average (range)	7.41×10^{7} (6.07×10^{7}–8.50×10^{8})	$7.26E^{7}$ (4.59×10^{7}–8.50×10^{7})	$1.01E^{8}$ (6.07×10^{7}–1.52×10^{8})	$7.39E^{7}$ (6.81×10^{7}–7.89×10^{7})
GIT tissue sites evaluated	6	6	5	6
GIT Area evaluated (μm^2): Average (range)	1.49×10^{8} (2.45×10^{6}–2.86×10^{8})	3.09×10^{7} (6.30×10^{6}–6.01×10^{7})	4.01×10^{7} (2.82×10^{6}–1.21×10^{8})	3.12×10^{7} (4.73×10^{6}–8.04×10^{7})

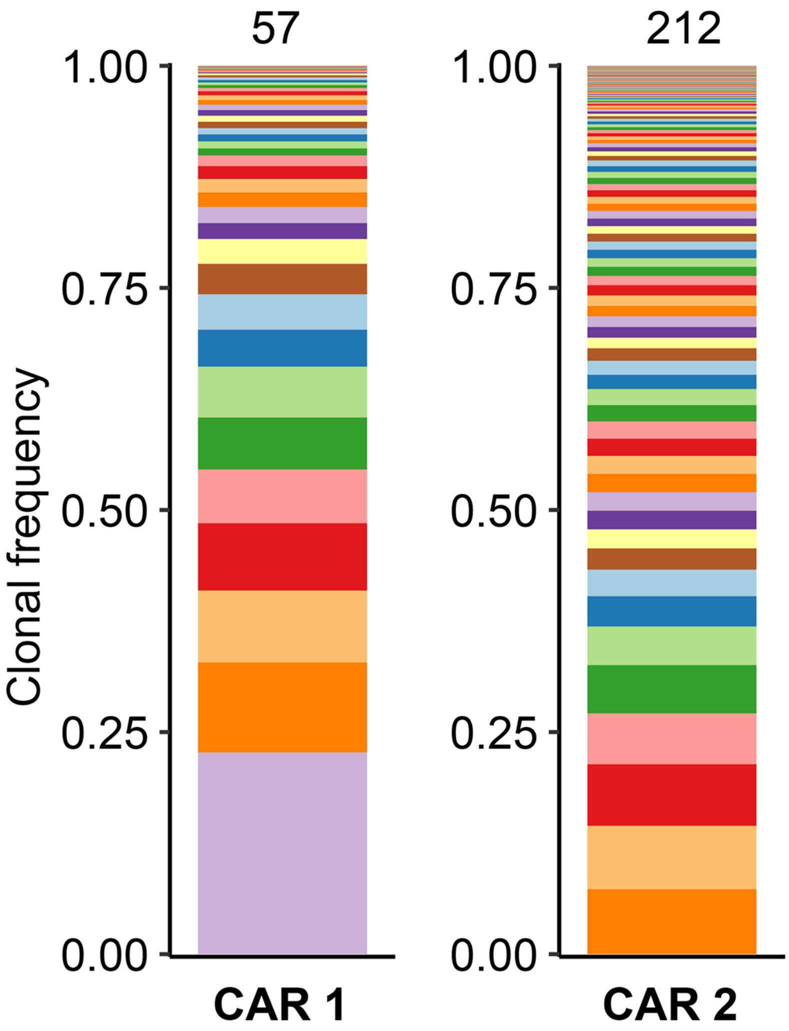

Supplemental Figure 1. Polyclonal repopulation of CD4CAR-modified, HSPC-derived PBMC in vivo. Clonal frequencies of unique vector integration site (VIS) clones in PBMC prior to SHIV challenge in NHP IDs CAR 1 (left) and CAR 2 (right). In the stacked bar plot, each color represents a unique HSPC-derived CD4CAR VIS clone, and thickness of the band corresponds to the clonal frequency or abundance of that CAR-modified HSPC clone within the CAR$^+$ PBMC. Number on top of the stacked-bar plot indicate number of VIS clones detected by the VIS assay.

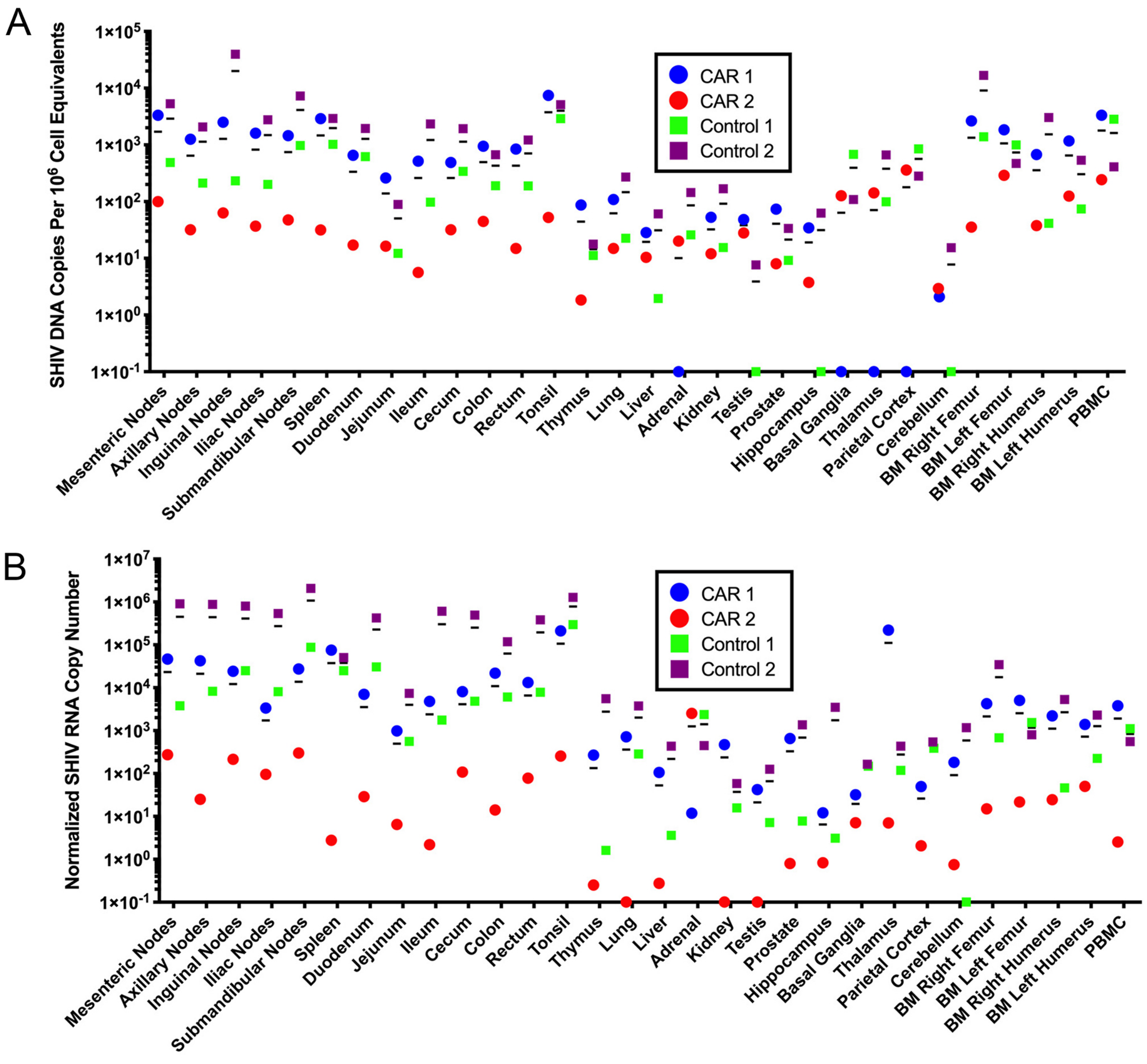

Supplemental Figure 2. PCR-based quantification of CD4CAR gene marking in necropsy tissues from CAR1/CAR2 and Control 1/Control 2. At necropsy, tissues from the indicated sites were collected and stored in nucleic acid preservative prior to homogenization and separate isolation of total DNA and RNA. Quantitative PCR assays were then used to measure the number of SHIV DNA copies per million cell equivalents **(A)** or normalized SHIV RNA copy number **(B)**. Macaque RNase P, p30 subunit (MRPP30) was used to calculate cell equivalents in (A), and MRPP30 crossing threshold (cT) values were used to normalize SHIV RNA data in (B).

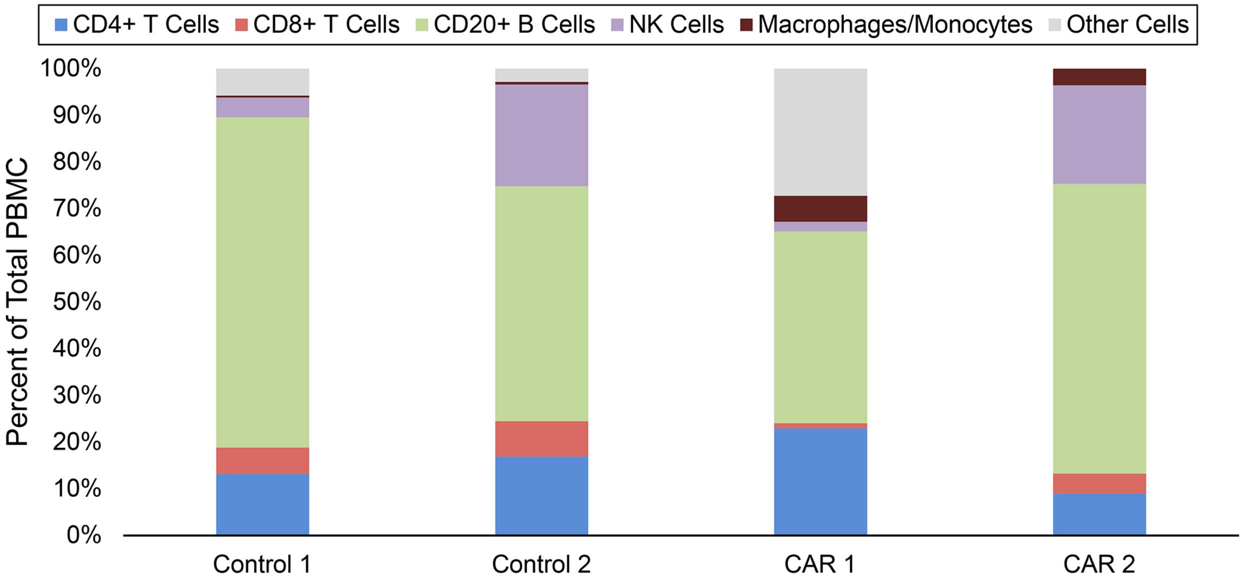

Supplemental Figure 3. PCR-based quantification of CD4CAR gene marking in necropsy tissues from CAR1/CAR2 and Control 1/Control 2. At necropsy, PBMC from the indicated animals were stained with cell surface markers to delineate CD4CAR$^+$ cells that were CD4$^+$ T cells (blue), CD8$^+$ T cells (red), CD20$^+$ B cells (green), CD2$^+$NKG2a$^+$ NK cells (purple) CD14$^+$ monocytes (brown) or lineage negative (gray) (n=4 macaques).

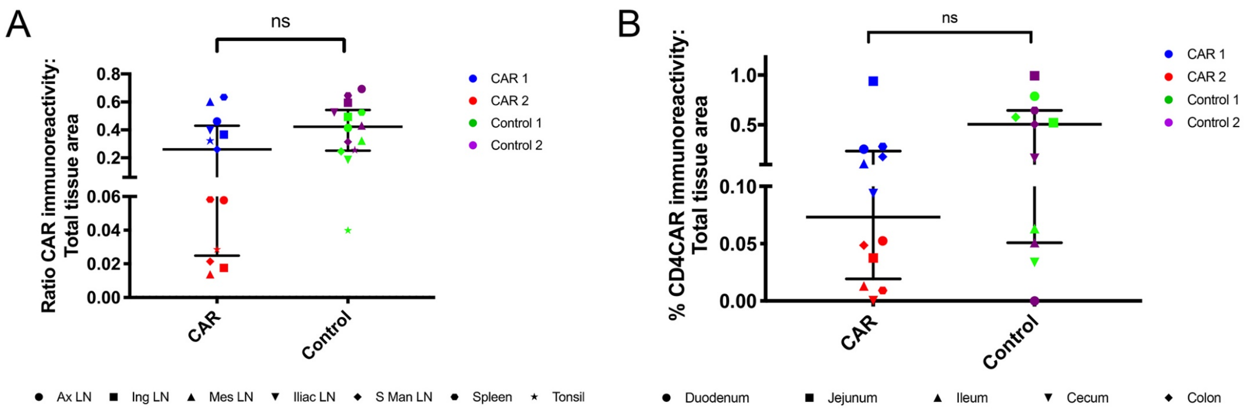

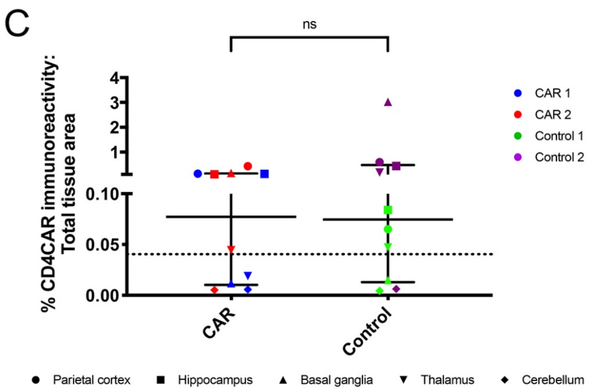

D

Mann Whitney test	Adjusted P Value	Significance
Lymphoid GC's	0.0945	ns
GIT	0.1693	ns
CNS	0.5228	ns

Supplemental Figure 4. Statistical comparison of total CD4CAR immunoreactive tissue area for CAR 1/CAR2 vs. Control 1/Control 2. Shown are merged data from CAR 1/CAR 2 and Control 1/Control (n= 4 macaques) corresponding to Figures 3 **(A)** (6-7 lymphoid tissues per macaque), 5 **(B)** (5-6 GIT tissues per macaque), and 7 **(C)** (5 CNS tissues per macaque). Statistical significance was calculated between pooled tissue sites from each pair of animals via Mann-Whitney test (unpaired, two-tailed) **(D)**, consistent with our previous statistical methods (10). Charts show individual data points, with bars illustrating the median + IQR.

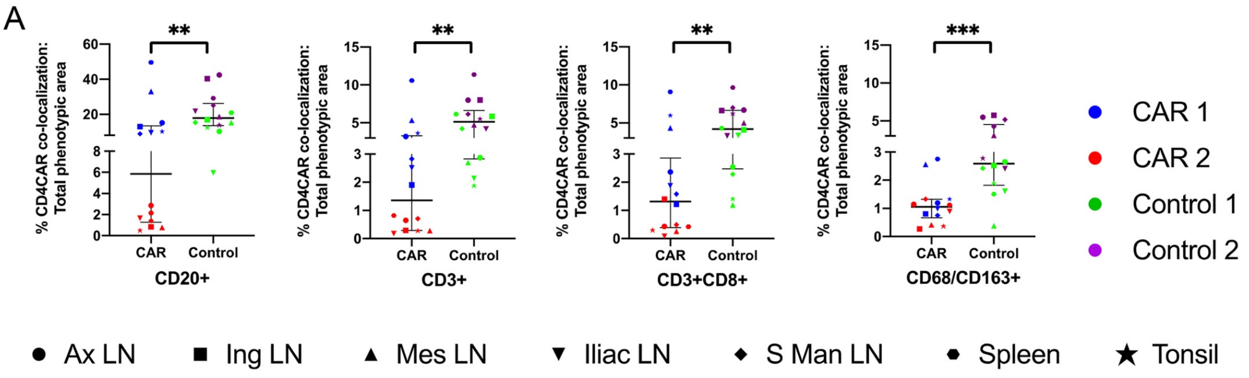

B

Mann Whitney test	Adjusted P Value	Significance
CD20⁺ B cells	0.0035	**
CD3⁺ T cells	0.0024	**
CD3⁺CD8⁺ T cells	0.0058	**
CD68/CD163⁺ Myeloid Cells	0.0004	***

Supplemental Figure 5. Statistical comparison of subset-specific CD4CAR immunoreactive lymphoid GC area for CAR 1/CAR2 vs. Control 1/Control 2. Shown are merged data from CAR 1/CAR 2 and Control 1/Control corresponding to Figure 4 **(A)**. Statistical significance was calculated between pooled CD20$^+$ B cell, CD3$^+$ and CD3$^+$CD8$^+$, T cell, and CD68/CD163$^+$ myeloid cell subset data from each pair of animals via Mann-Whitney test (unpaired, two-tailed) **(B)** (n=4 macaques; 6-7 lymphoid tissues per macaque), consistent with our previous statistical methods (10). Charts show individual data points, with bars illustrating the median + IQR.

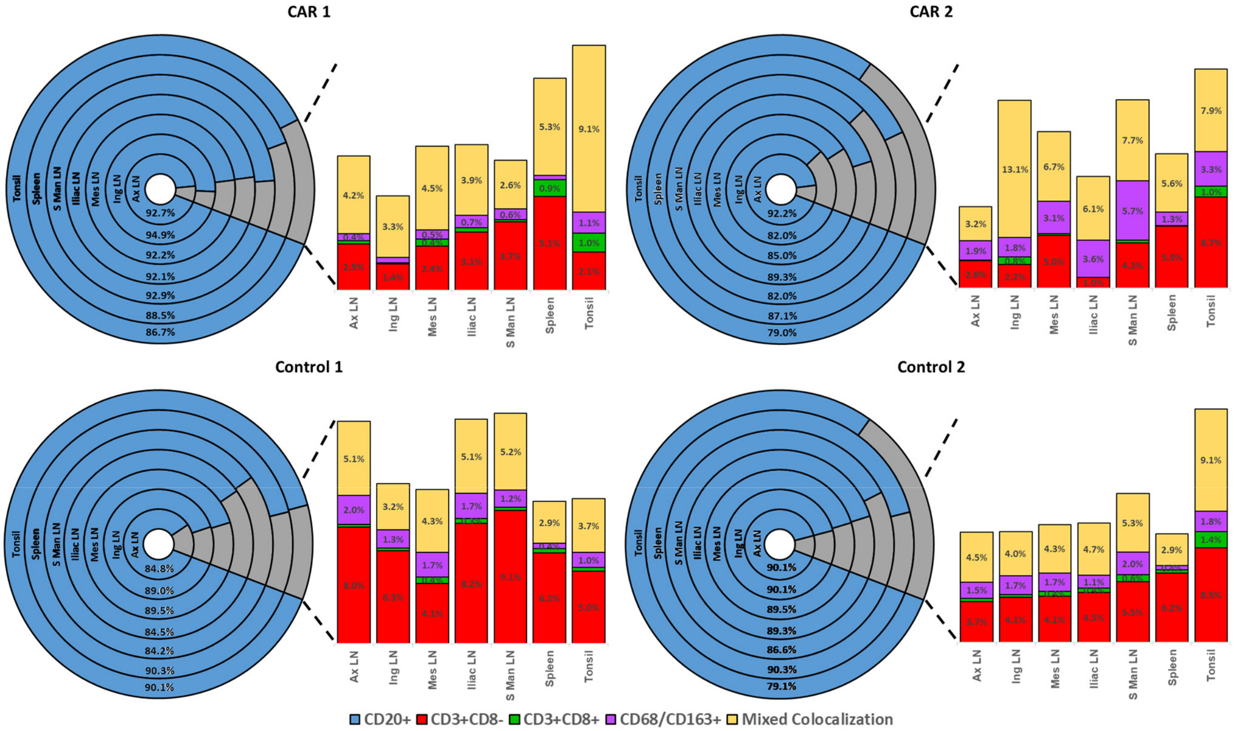

Supplemental Figure 6. Enriched engraftment of HSPC-derived CAR^{+} B-cells in lymphoid germinal centers. Distribution of CD4CAR colocalization in lymphoid germinal centers with markers of B-cells (CD20^{+}, blue), CD4 and double negative T-cells (CD3^{+}CD8^{-}, red), CTL (CD3^{+}CD8^{+}, green), and monocytes/macrophages (CD68/CD163^{+}, purple). Yellow: mixed colocalization, indicating CD4CAR pixel area that colocalized with more than one set of phenotypic markers (n=4 macaques; 6-7 lymphoid tissues per macaque).

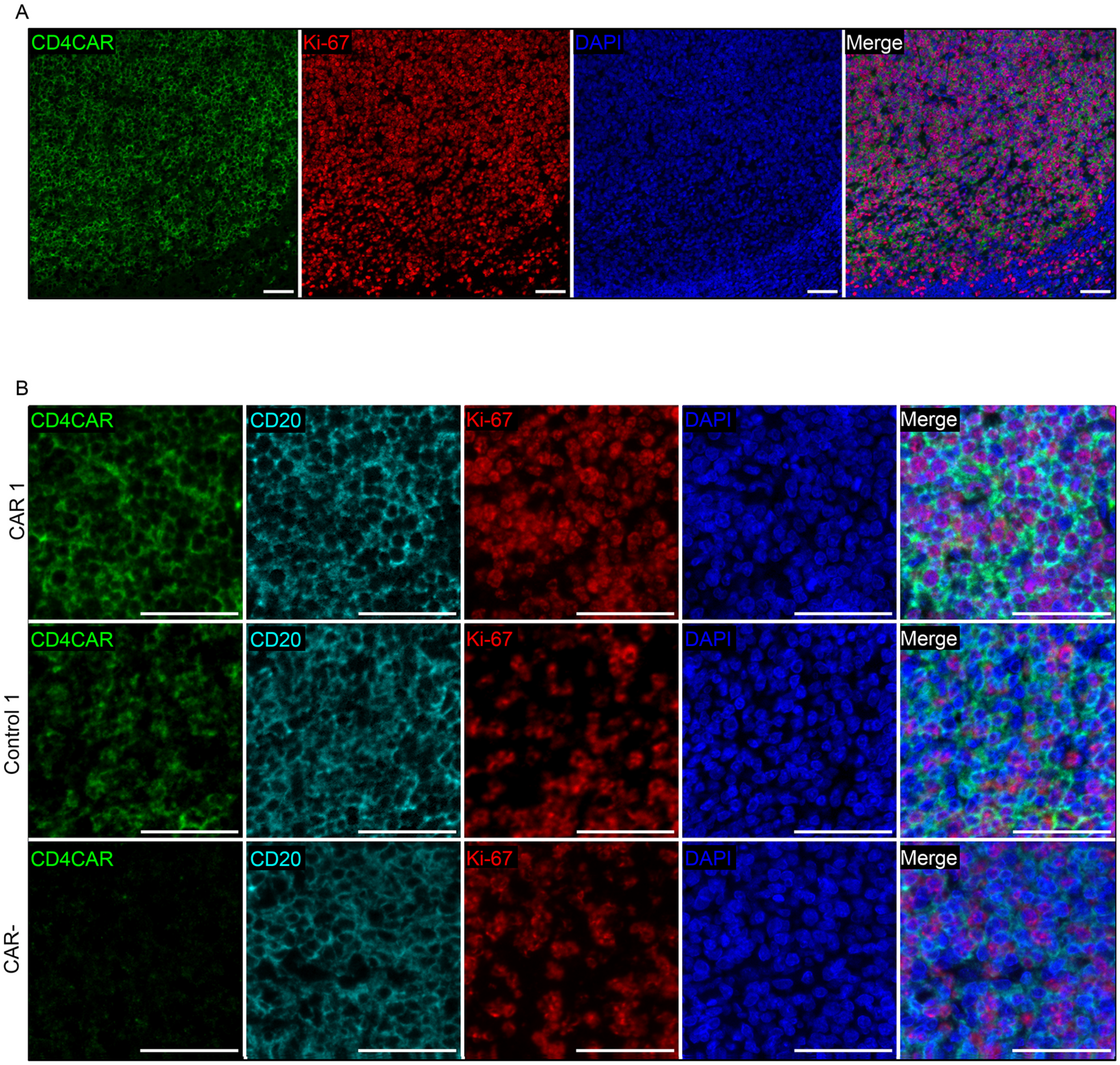

Supplemental Figure 7. HSPC-derived CAR$^+$ cells in lymphoid GC express the proliferation marker Ki-67. **A)** Fluorescent mIHC photomicrographs of CD4CAR (green), Ki-67 (red), and DAPI nuclear counterstain (blue) from CAR 1 spleen. **B)** Fluorescent mIHC photomicrographs of CD4CAR (green), CD20 (cyan), Ki-67 (red), and DAPI nuclear counterstain (blue); from CAR 1 and Control 1 spleen sections, as well as from a control animal that expressed neither CD4CAR nor CD4CARΔζ (CAR$^-$) (n=4 macaques, 7 lymphoid tissues per macaque). Scale bar: 50µm.

A

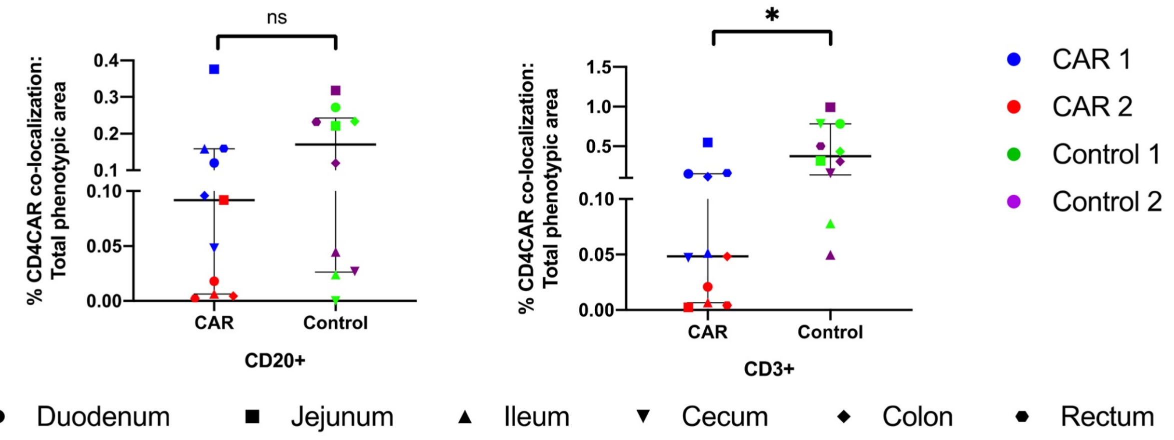

B

Mann Whitney test	Adjusted P Value	Significance
$CD20^+$ B cell	0.3494	ns
$CD3^+$ T cell	0.0037	**

Supplemental Figure 8. Statistical comparison of subset-specific CD4CAR immunoreactive GIT area for CAR 1/CAR2 vs. Control 1/Control 2. Shown are merged data from CAR 1/CAR 2 and Control 1/Control corresponding to Figure 4 **(A)**. Statistical significance was calculated between pooled $CD20^+$ B cell and $CD3^+$ T cell subset data from each pair of animals via Mann-Whitney test (unpaired, two-tailed) **(B)** (n= 4 macaques; 5-6 GIT tissues per macaque), consistent with our previous statistical methods (10). Charts show individual data points, with bars illustrating the median + IQR.

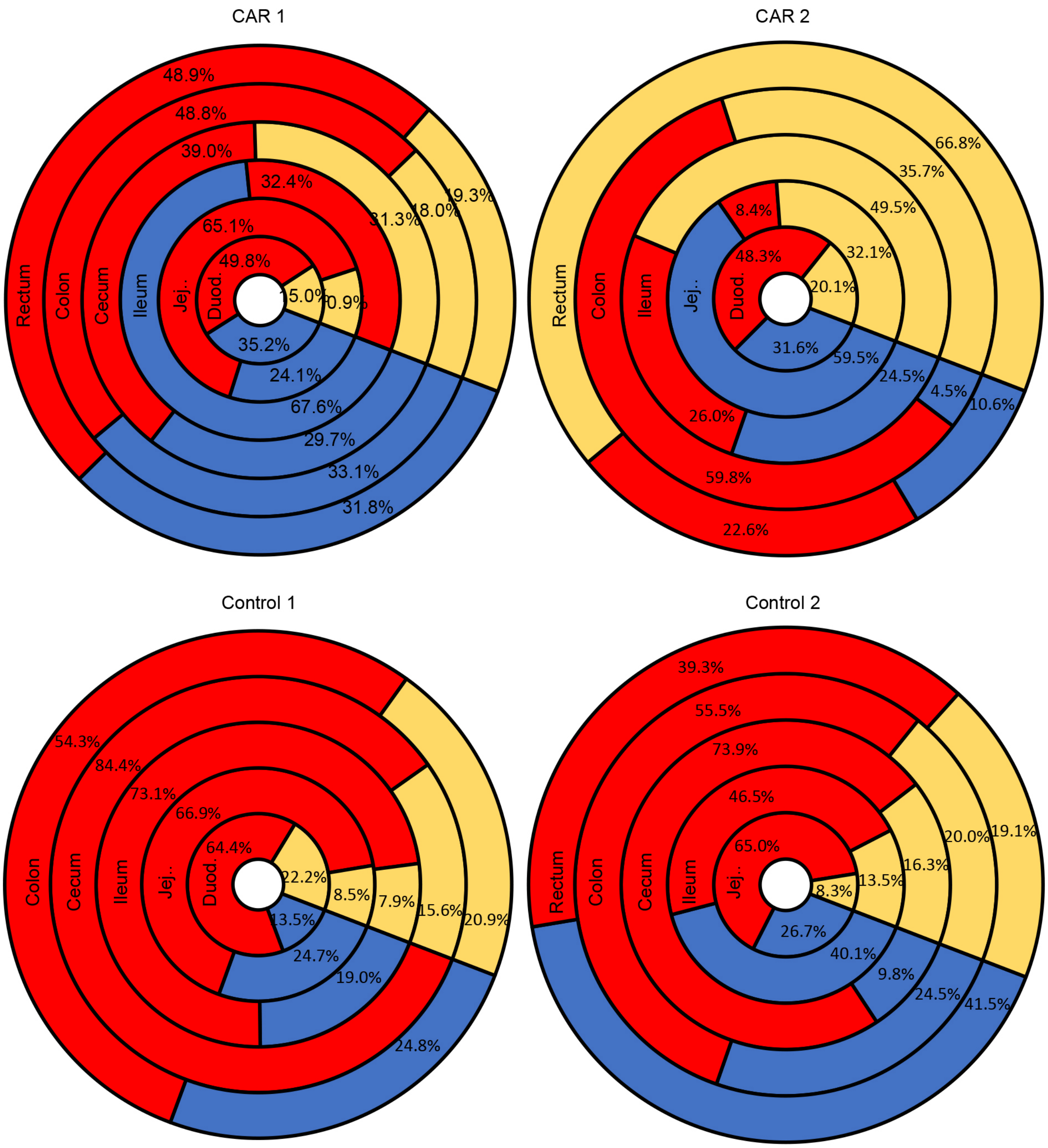

Supplemental Figure 9. Enriched engraftment of HSPC-derived CAR$^+$ T-cells in gut associate lymphoid tissues. Distribution of CD4CAR colocalization in gut associated lymphoid tissues with markers of B-cells (CD20$^+$, blue), and T-cells (CD3$^+$, red). Yellow: mixed colocalization, indicating CD4CAR pixel area that colocalized with both CD20 and CD3 phenotypic markers (n=4 macaques; 5-6 gastrointestinal tract tissues per macaque).

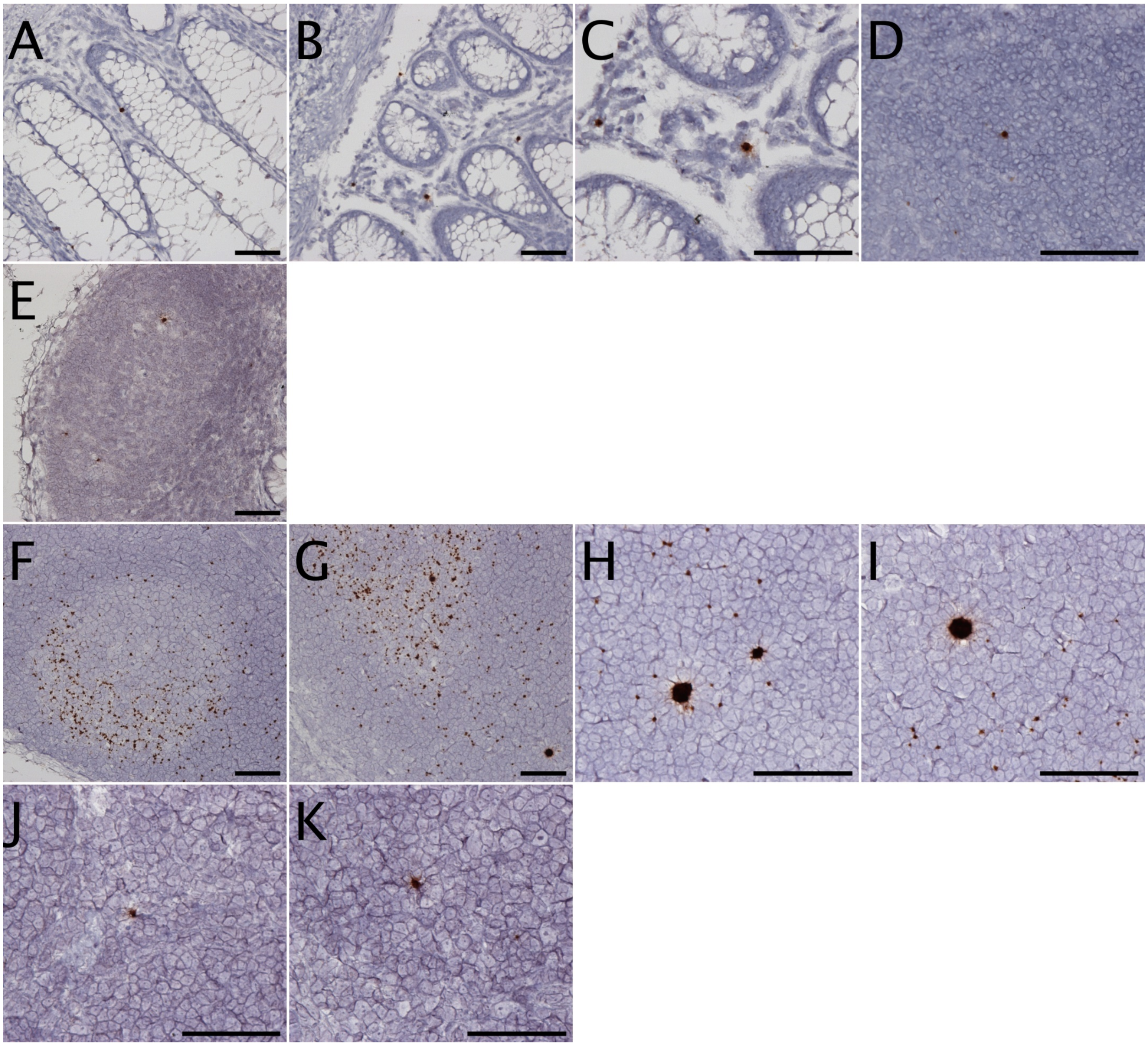

Supplemental Figure 10. RNAscope and DNAscope detection of SHIV nucleic acids *in situ.* At necropsy, tissues from the same sites assayed for CD4CAR expression were stained in parallel for SHIV RNA and DNA using RNAscope and DNAscope methods, respectively. Shown are representative images from CAR 2 colon SHIV RNAscope (**A-D**) and DNAscope (**E**), and Control 1 mesenteric lymph node RNAscope (**F-I**) and DNAscope (**J-K**). Scale bar: 50μm.

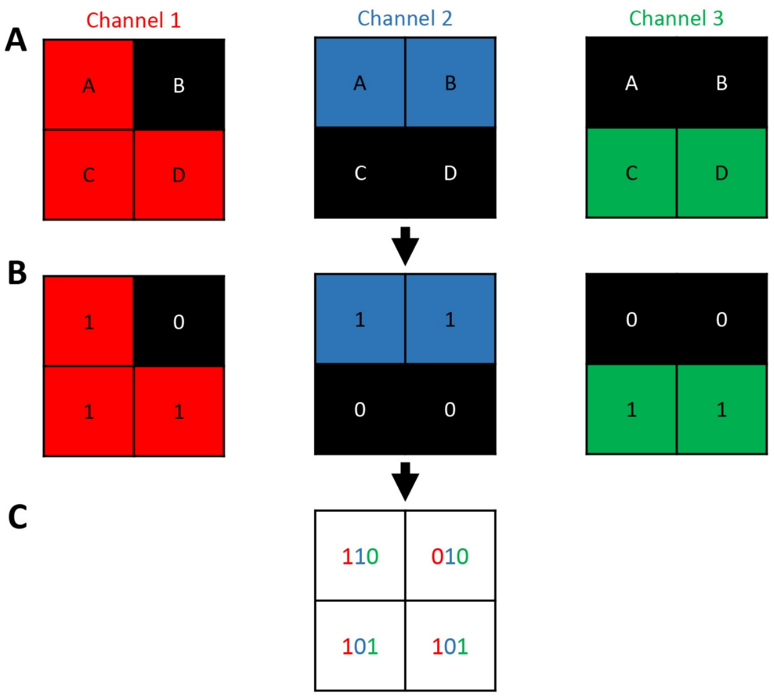

D

Binary Number (Array Index)	Colocalization Pattern (Channels)	Pixel Count (Array Contents)
000	None	0
001	3	0
010	2	1
011	3,2	0
100	1	0
101	1,3	2
110	1,2	1
111	1,2,3	0

Supplemental Figure 11. Conceptual schematic for the binarized pixel colocalization algorithm performed by the Multiplex Pixel Colocalization plugin. **A)** Example analysis for a hypothetical, binarized, 3 channel photomicrograph. Pixels in each channel are assigned either a “1” or a “0” based on the presence (1) or absence (0) of fluorescent signal in that pixel **(B)**. Binary numbers are generated from each pixel position based on assigned number in each channel **(C)**, which are used to increment the count in an array of 2^n potential outcomes; where *n* is the number of channels being evaluated for colocalization **(D)**.
